# Supplementary material for: Electrochemical Radical Tandem Difluoroethylation/Cyclization of Unsaturated Amides to Access MeCF2-Featured Indolo/Benzoimidazo [2,1-a]Isoquinolin-6(5H)-ones
Source: Molecules. 2024 Feb 22;29(5):973. doi: 10.3390/molecules29050973 (PMC10934937; doi:10.3390/molecules29050973)

Supporting Information

Electrochemical Radical Tandem Difluoroethylation/ Cyclization of Unsaturated Amides to Access MeCF2-Featured Indolo/Benzoimidazo [2,1-a]Isoquinolin-6(5H)-ones

Yunfei Tian 1,*, Dongyu Guo 2, Luping Zheng 1, Shaolu Yang 1, Ningning Zhang 1, Weijun Fu 1 and Zejiang Li 2

[1]Key Laboratory of Fuction-Oriented Porous Materials of Henan Province, College of Chemistry and Chemical Engineering, Luoyang Normal University, Luoyang 471934, China; zhen-glp0213@163.com (L.Z.); 13525275309@163.com (S.Y.); n18736243401@126.com (N.Z.); wjfu@lynu.edu.cn (W.F.)

[2]
Key Laboratory of Medicinal Chemistry and Molecular Diagnosis of the Ministry of Education, State Key Laboratory of New Pharmaceutical Preparations and Excipients, Key Laboratory of Chemical Biology of Hebei Province, College of Chemistry & Materials Science, Hebei University, Baoding 071002, China; 18712762730@163.com (D.G.); lizejiang898@126.com (Z.L.)

Table of Contents

1. Radical capturing experiments…..S2

2. Cyclic voltammetry experiments…..S3

3.Photo of of the electrochemical setup…..S4

4. Crystallographic details of product **3m**…..S4

5.Crystallographic details of product **5i**…...S6

6. Copies of the ^{1}H NMR, ^{19}F NMR, ^{13}C NMR...S9-S55

1. Radical capturing experiments

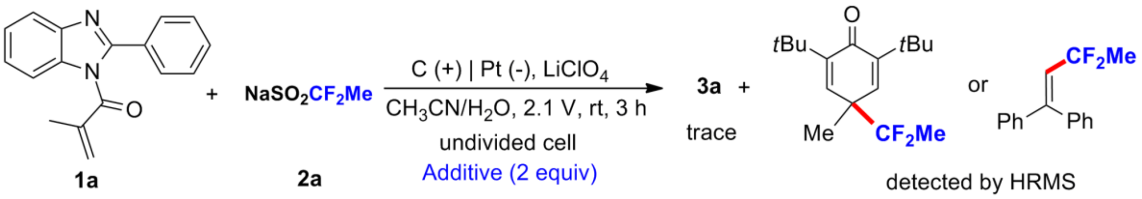

To a 20 mL test tube with a stir bar was charged with, 2-arylbenzimidazoles (1 equiv., 0.2 mmol), $MeCF_2SO_2Na$, or sodium cyclopropyldifluoromethylsulfinate (3 equiv., 0.6 mmol), $LiClO_4$ (0.3 M), MeCN (4.5 mL), H_2O (1.5 mL). The tube was equipped with a carbon plate (10 mm * 10 mm * 3 mm) as the anode and a platinum plate (10 mm × 10 mm × 0.2 mm) as the cathode. The reaction mixture was electrolyzed in an undivided cell at room temperature under a constant voltage of 2.1 V for 3 h. Upon completion, the mixture was extracted with EtOAc (10 mL × 3). The combined organic phases were dried over Na_2SO_4 and condensed under vacuum. The difluoroethylation of 2-arylbenzimidazoles was almost prohibited and the adduct products were detected by HRMS analysis (Figure S1)

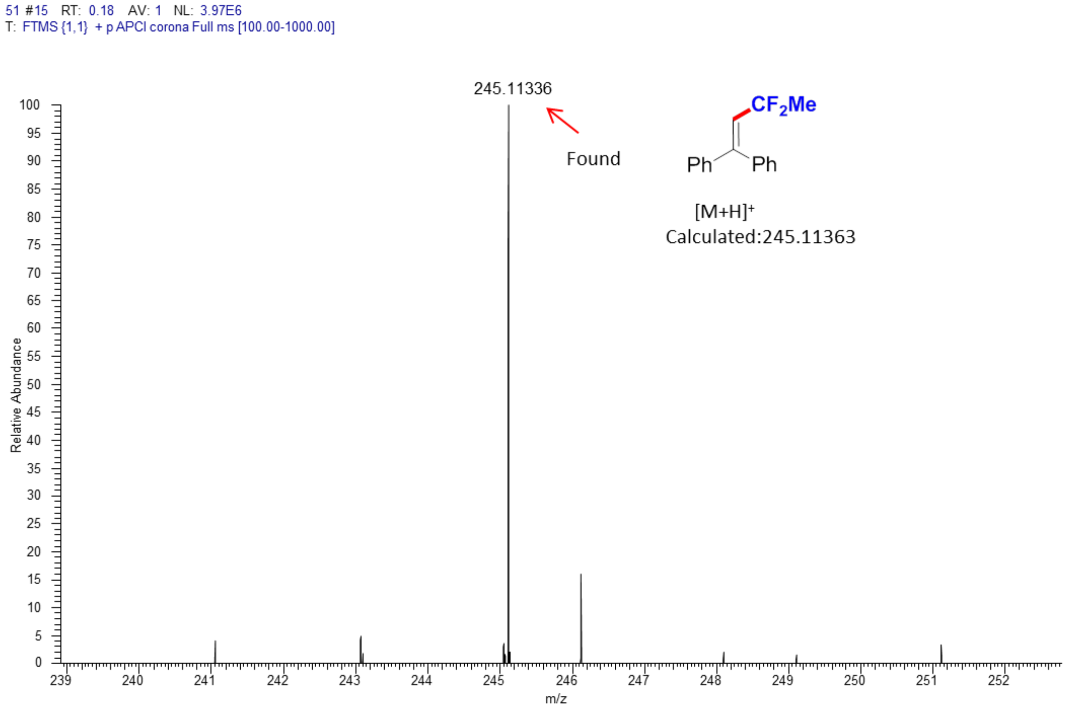

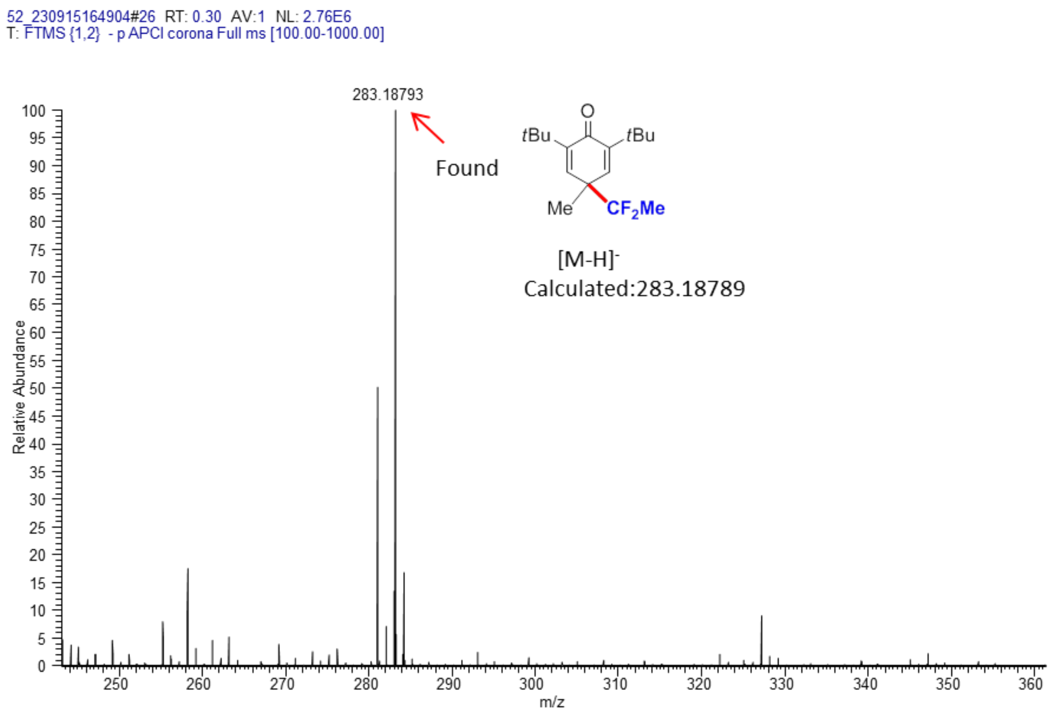

Figure S1. HRMS analysis of adduct products

2. Cyclic voltammetry experiments

The cyclic voltammograms experiments were performed in a three-electrode system with an electrochemical workstation (CHI660E, Shanghai, China) under air at room temperature. A glassy carbon electrode working electrode, a platinum wire counter electrode, and an Ag/$AgNO_3$ reference electrode were used. The scan rate was 50 mV/s, from 0 V to 2 V. 5 mM analyte and 0.1 M $LiClO_4$ was dissolved in the mixture of acetonitrile and H_2O.

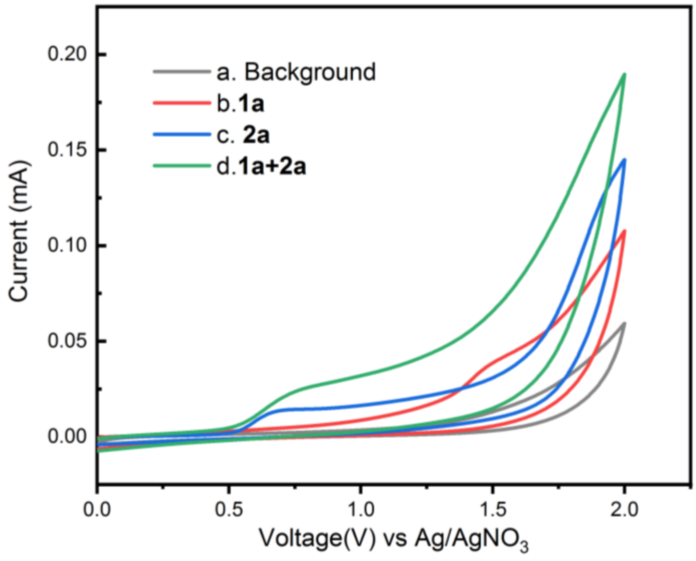

Figure S2. Cyclic voltammograms of substrates: (a) Background (0.1 M $LiClO_4$ in CH_3CN/H_2O (10/1,

V/V)), (b) **1a** (5 mM), (c) **2a** (5 mM), (d) **1a** (5 mM)+**2a** (5 mM).

3. Photo of of the electrochemical setup

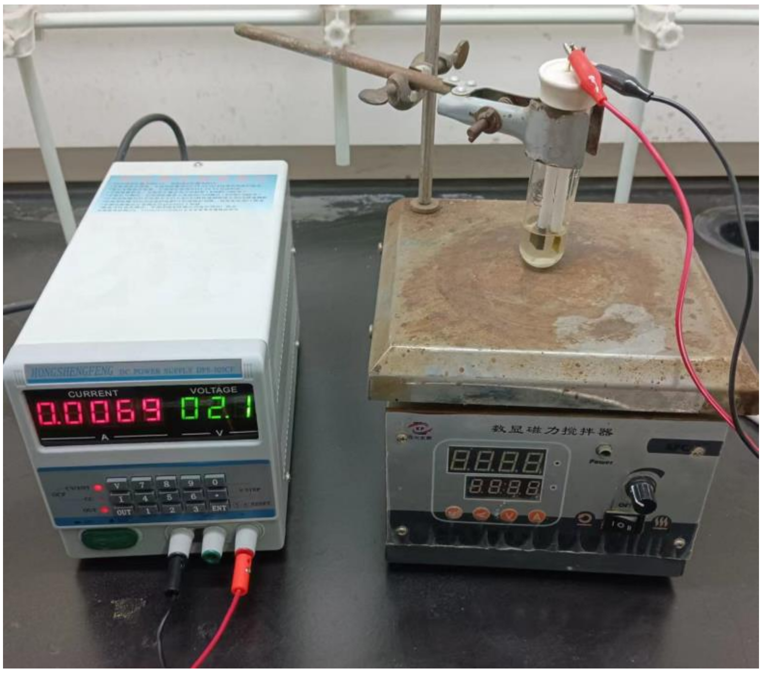

4. Crystallographic details of product 3m

(1) Product **3m** was solved with 1 mL dichloromethane in a sample bottle. Then 2 mL petroleum ether was added to the above mixture. Finally, the sample bottle was sealed and placed in a dark cabinet. The crystal was precipitated via volatilizing after several days.

(2) A single crystal of $C_{19}H_{15}BrF_2N_2O$ [Product **3m**] was offered as follows: A proper crystal was selected and detected on an Oxford Diffraction Gemini. The crystal stayed at 293.0 K during data collection. With the assistance of Shelxtl, the structure was solved with the XShell structure solution program using Charge Flipping, and it was refined with the SHELXL [1] refinement package using Least Squares minimization. Finally, crystal data and structure refinement parameters of product **3m** are described as depicted in Table S1. CCDC No. 2303467. This material is available free of charge via the Internet at http://www.ccdc.cam.ac.uk/.

Table S1. Crystal data of compound **3m**.

Empirical formula	$C_{19}H_{15}BrF_2N_2O$
Formula weight	405.24
Temperature/K	293(2)

Crystal system	orthorhombic
Space group	Pbca
a/Å	13.5964(9)
b/Å	13.6099(7)
c/Å	18.1837(8)
α/ °	90
β/ °	90
γ/ °	90
Volume/Å^3	3364.8(3)
Z	8
ρ_{calc} (g cm^{-3})	1.600
μ (mm^{-1})	2.473
F (000)	1632.0
Crystal size/mm^3	0.29 × 0.28 × 0.23
Radiation	Mo Kα (λ = 0.71073)
2Θ range for data collection/ °	6.7 to 58.334
Index ranges	-17 ≤ h ≤ 17, -15 ≤ k ≤ 17, -24 ≤ l ≤ 23
Reflections collected	17858
Independent reflections	4072 [Rint = 0.0650, Rsigma = 0.0880]
Data/restraints/parameters	4072/0/228
Goodness-of-fit on F^2	1.016
Final R indexes [$I \geq 2\sigma(I)$]	R_1 = 0.0590, wR_2 = 0.1029
Final R indexes [all data]	R_1 = 0.1513, wR_2 = 0.1314
Largest diff. peak/hole / e Å^{-3}	0.40/-0.39
CCDC	2303467

Displacement ellipsoids are drawn at the 50% probability level

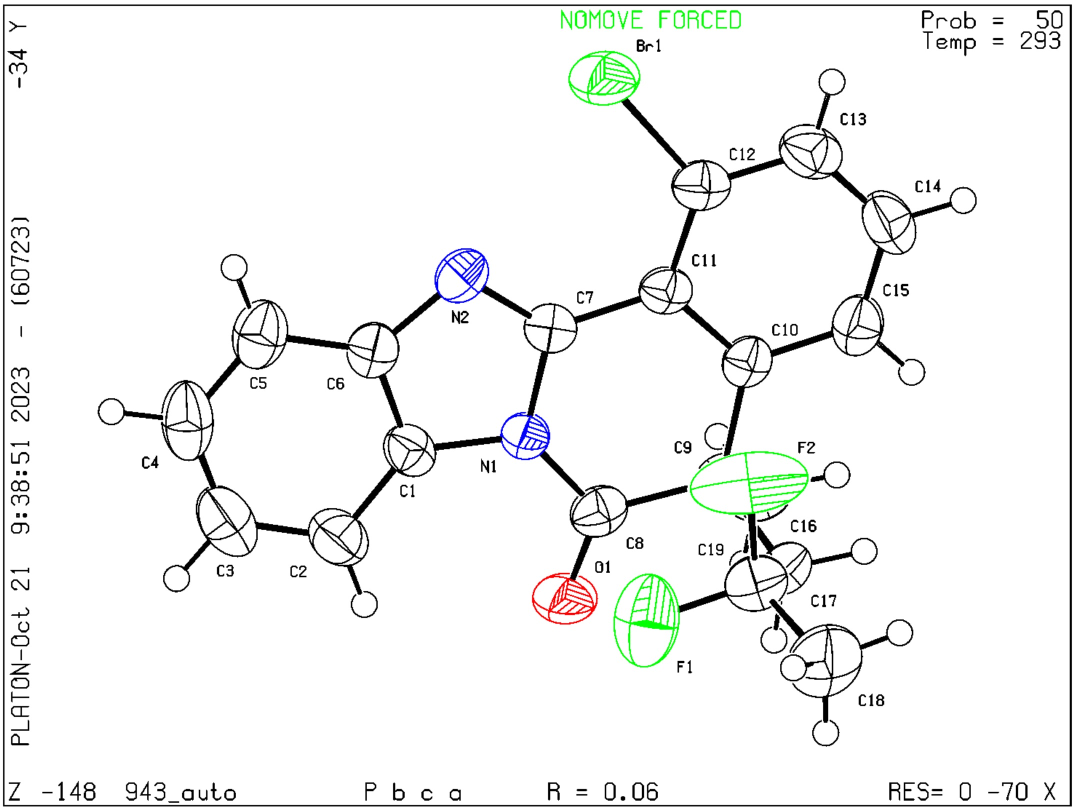

Fig. S3 Structure of product **3m**

5. Crystallographic details of product 5i

(1) Product **5i** was solved with 1 mL of dichloromethane in a sample bottle. Then 2 mL petroleum ether was added to the above mixture. Finally, the sample bottle was sealed and placed in a dark cabinet. The crystal was precipitated via volatilizing after several days.

(2) A single crystal of $C_{23}H_{20}ClF_2NO$ [Product **5i**] was offered as follows: A proper crystal was selected and detected on a 'D8 VENTURE ' diffractometer. The crystal stayed at 150.0 K during data collection. With the assistance of Shelxtl, the structure was solved with the XShell structure solution program using Charge Flipping, and it was refined with the SHELXL [1] refinement package using Least Squares minimization. Finally, crystal data and structure refinement parameters of product **5i** are described as depicted in Table S2. CCDC No. 2307007. This material is available free of charge via the Internet at http://www.ccdc.cam.ac.uk/.

Table S2. Crystal data of compound **5i**.

Empirical formula	$C_{23}H_{20}ClF_2NO$
Formula weight	399.85

Temperature/K	150
Crystal system	monoclinic
Space group	$P2_1/c$
a/Å	9.1778(4)
b/Å	10.0556(5)
c/Å	20.7235(11)
α/ °	90
β/ °	94.132(2)
γ/ °	90
Volume/Å^3	1907.57(16)
Z	4
ρ_{calc} (g cm^{-3})	1.392
μ (mm^{-1})	0.233
F (000)	832.0
Crystal size/mm^3	0.15 × 0.08 × 0.06
Radiation	Mo Kα (λ = 0.71073)
2Θ range for data collection/ °	3.942 to 52.848
Index ranges	$-11 \leq h \leq 11$, $-12 \leq k \leq 12$, $-25 \leq l \leq 22$
Reflections collected	15768
Independent reflections	3873 [Rint = 0.0667, Rsigma = 0.0561]
Data/restraints/parameters	3873/0/255
Goodness-of-fit on F^2	1.037
Final *R* indexes [$I \geq 2\sigma(I)$]	R1 = 0.0460, wR2 = 0.1122
Final *R* indexes [all data]	R1 = 0.0596, wR2 = 0.1214
Largest diff. peak/hole / e Å^{-3}	0.30/-0.31
CCDC	2307007

Displacement ellipsoids are drawn at the 50% probability level

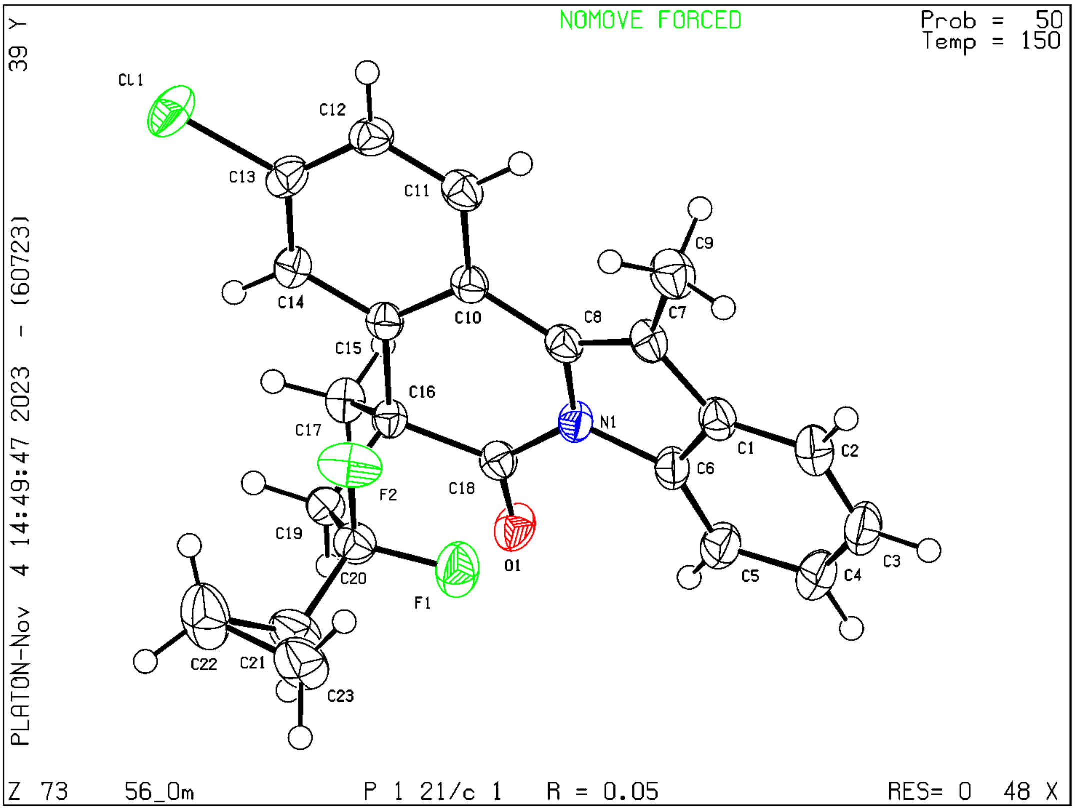

Fig. S4 Structure of product **5i**

6. Copies of the ^{1}H NMR, ^{13}C NMR and ^{19}F NMR

3a-^{1}H NMR (400 MHz, $CDCl_3$)

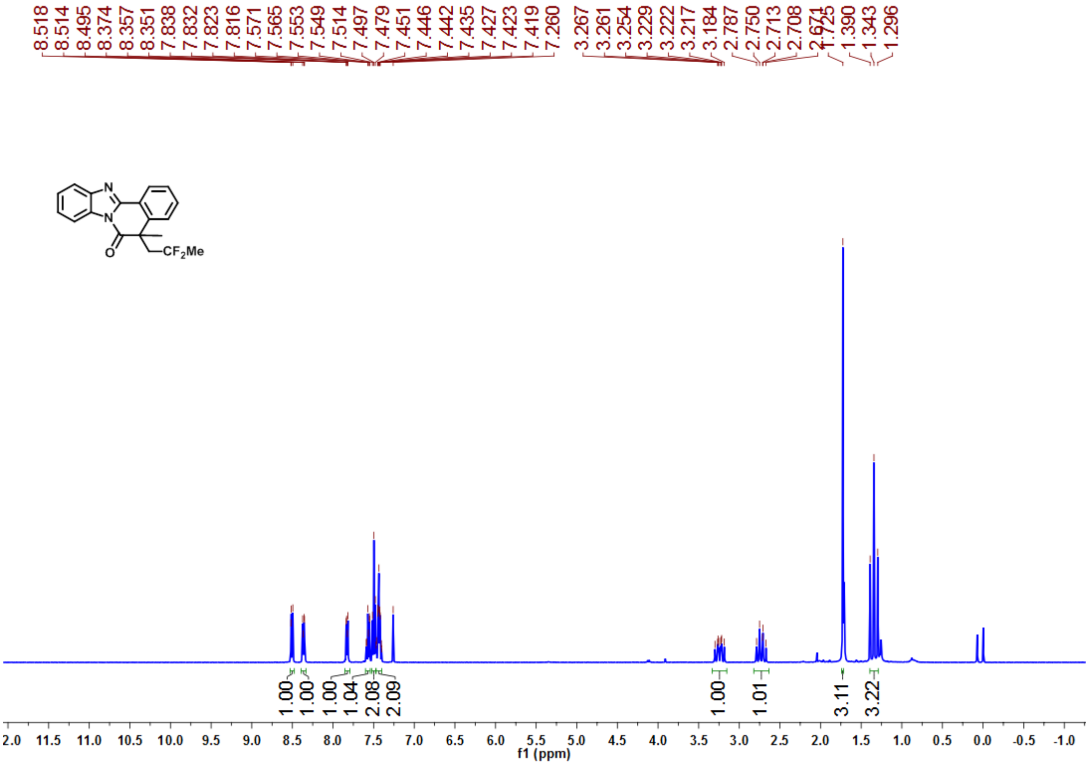

3a-$^{13}C\{^{1}H\}$ NMR (100 MHz, $CDCl_3$)

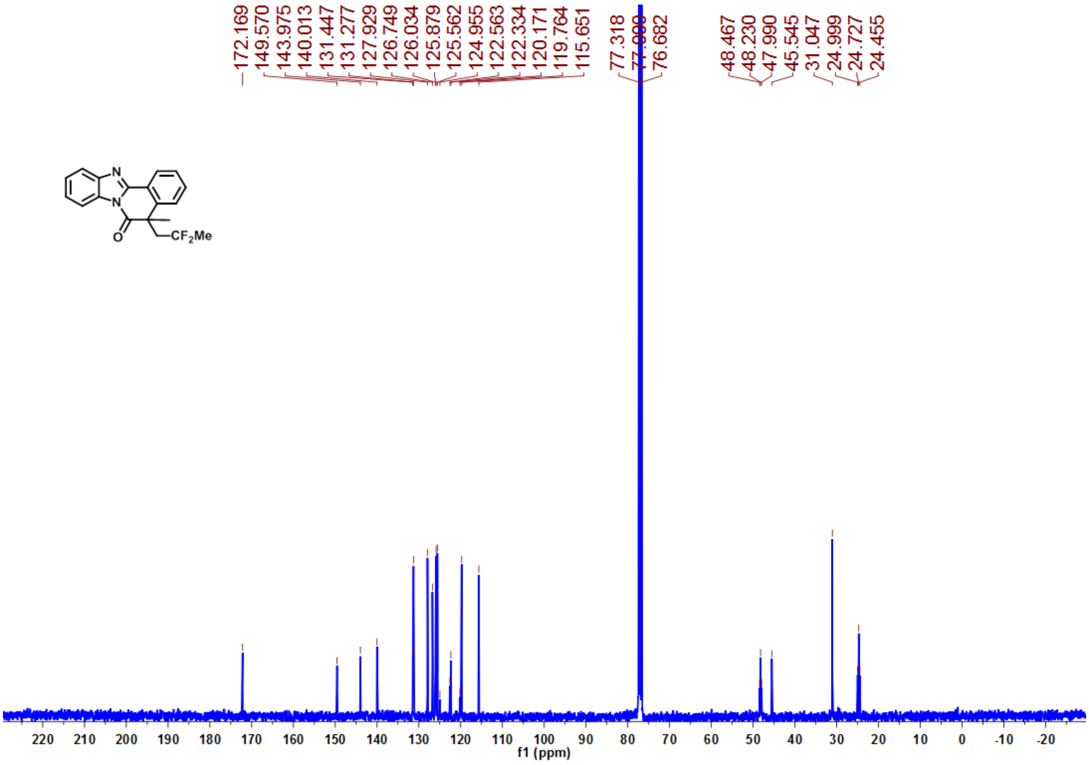

3a-^{19}F NMR (471 MHz, $CDCl_3$)

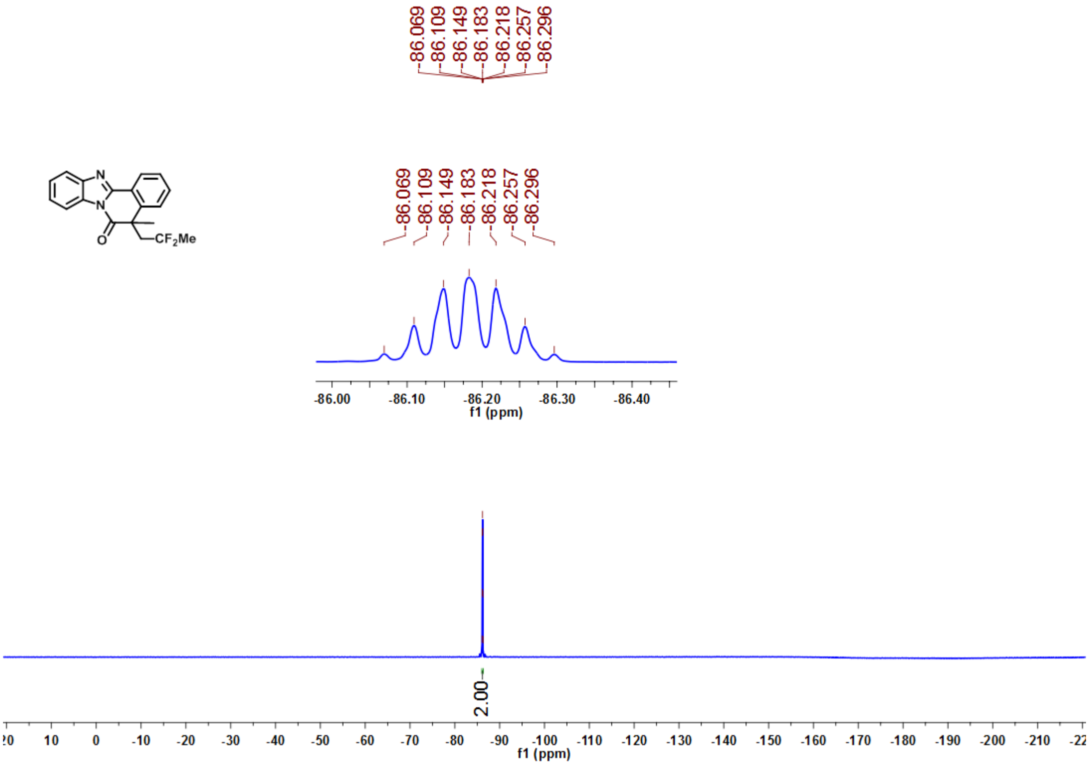

3b-^{1}H NMR (500 MHz, $CDCl_3$)

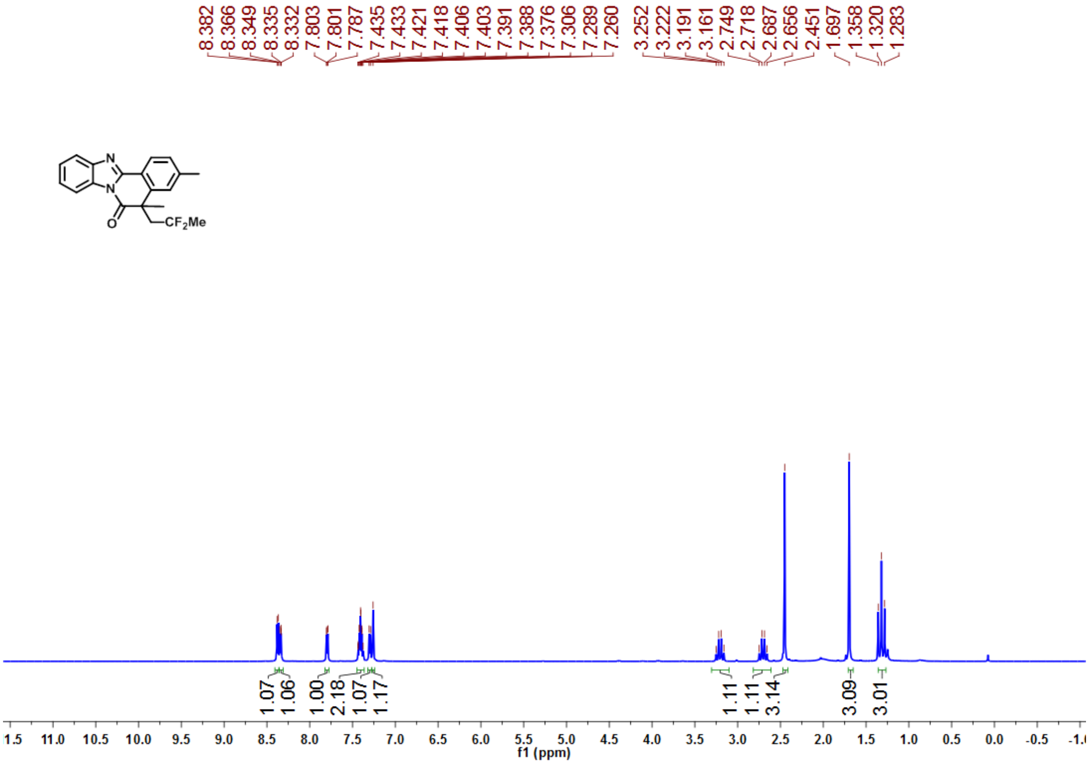

3b-^{13}C{^{1}H} NMR (125 MHz, $CDCl_3$)

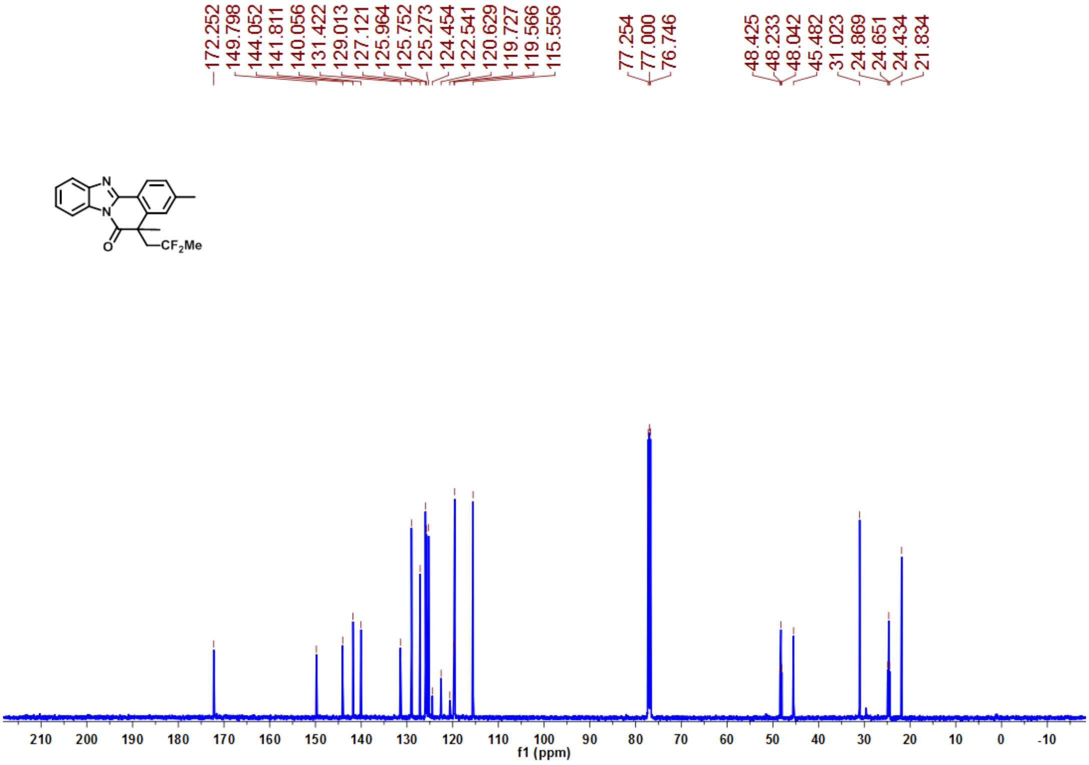

3b-^{19}F NMR (471 MHz, $CDCl_3$)

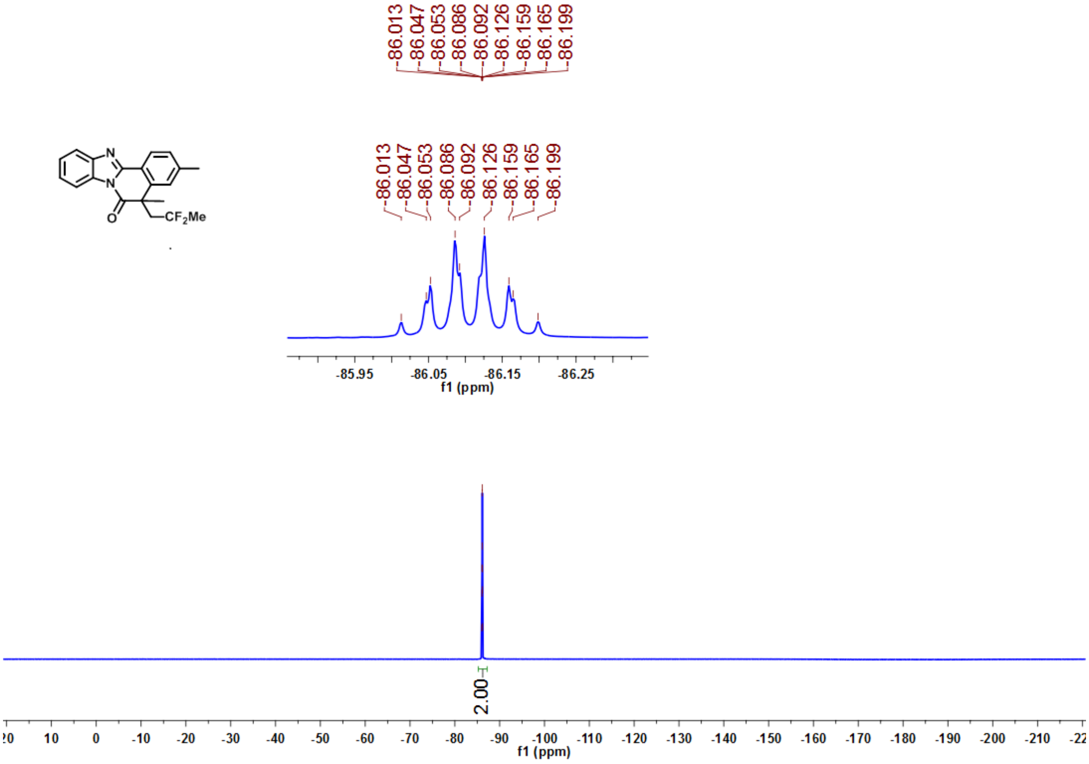

3c-^{1}H NMR (400 MHz, $CDCl_3$)

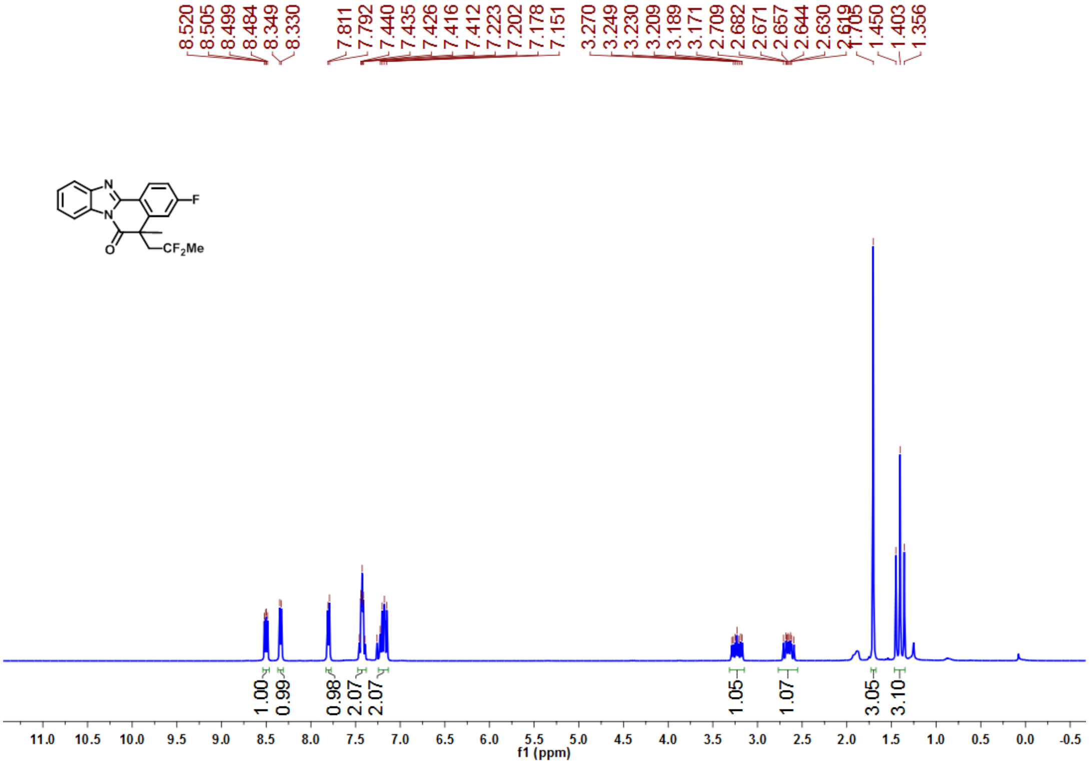

3c-^{13}C{^{1}H} NMR (100 MHz, $CDCl_3$)

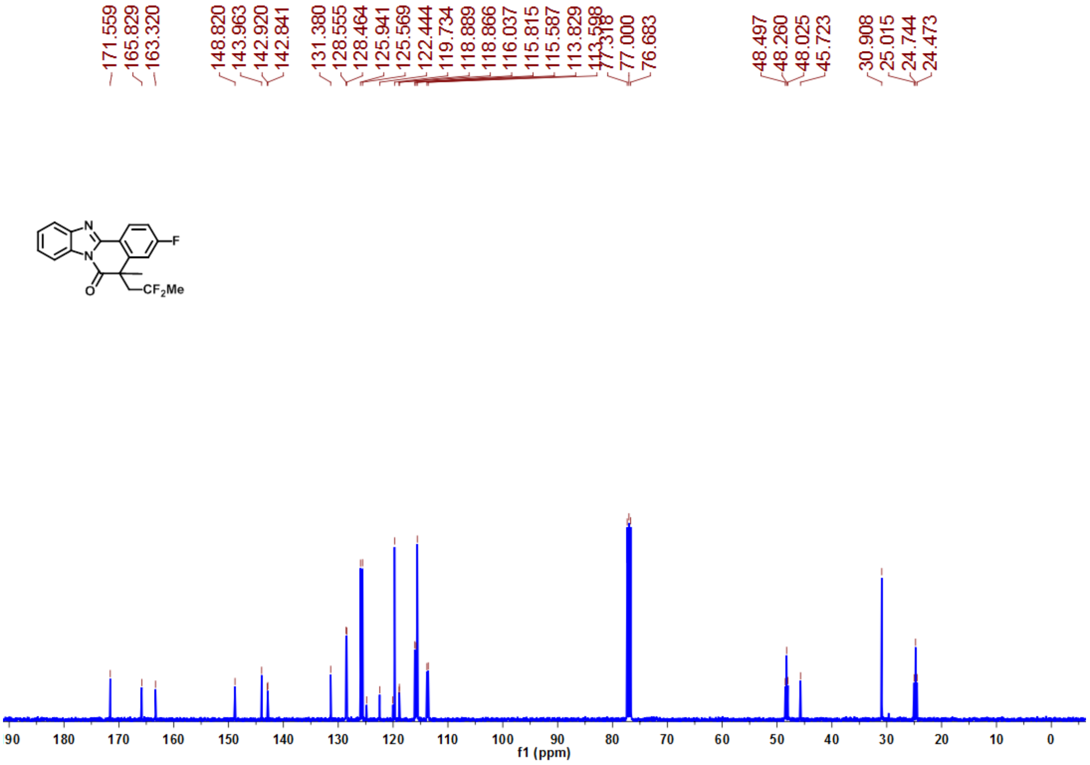

3c-^{19}F NMR (471 MHz, $CDCl_3$)

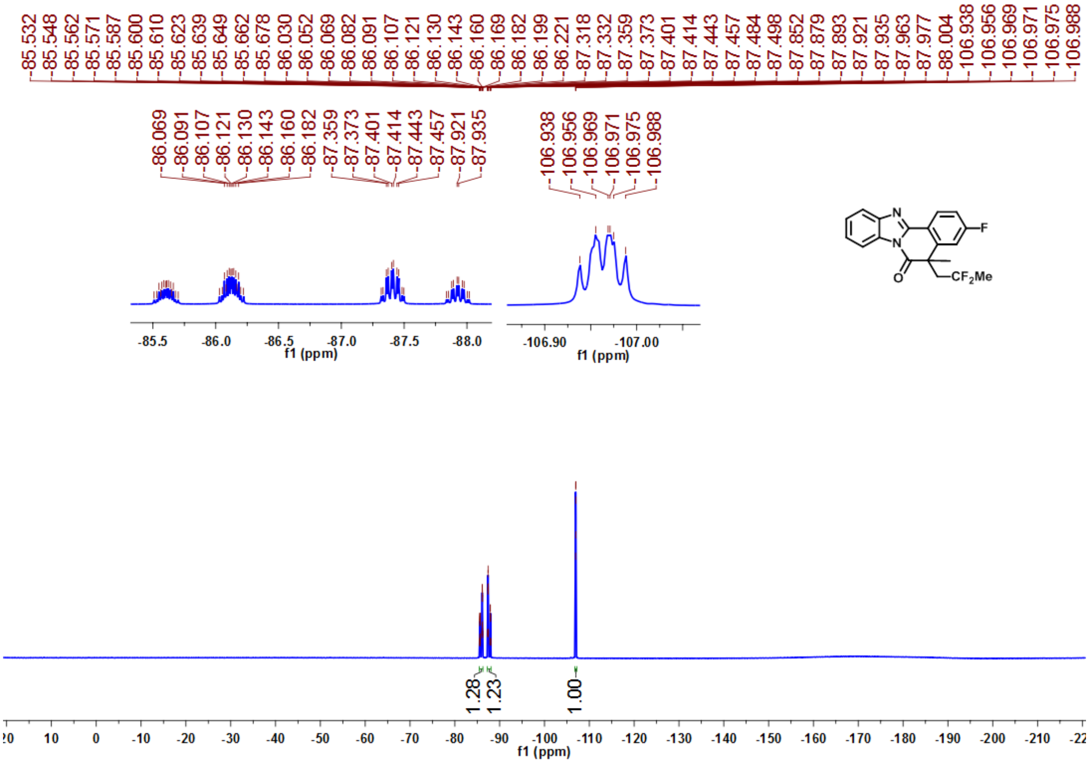

3d-^{1}H NMR (400 MHz, $CDCl_3$)

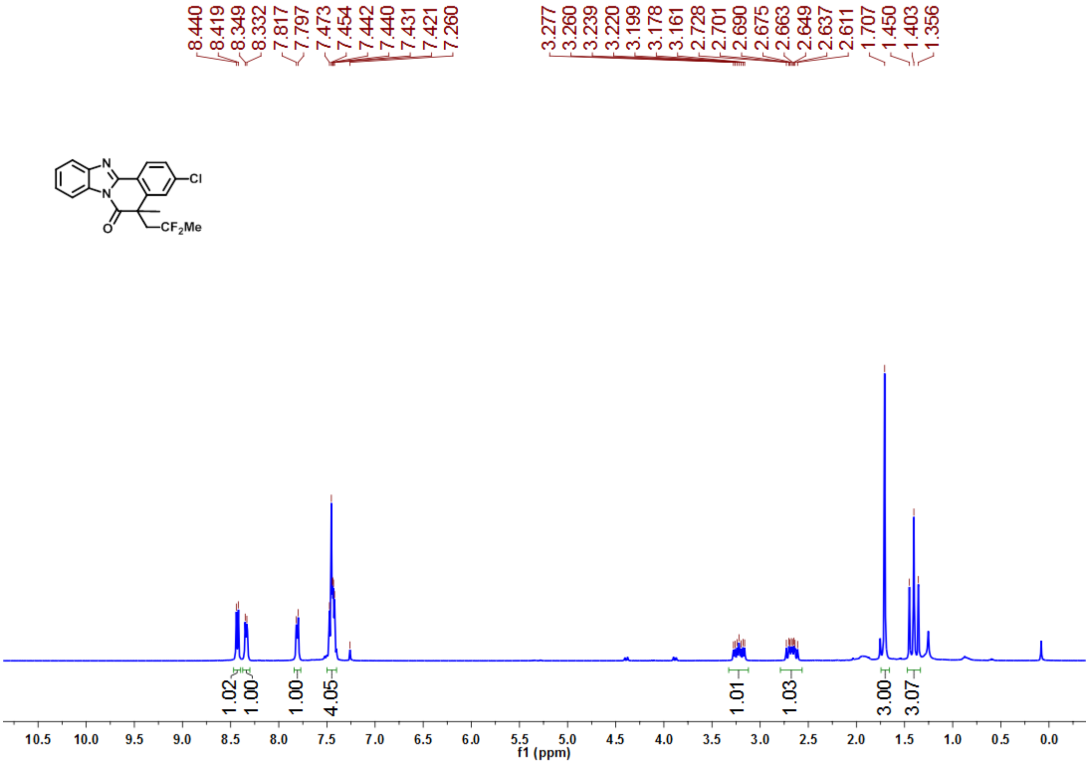

3d-$^{13}C\{^{1}H\}$ NMR (100 MHz, $CDCl_3$)

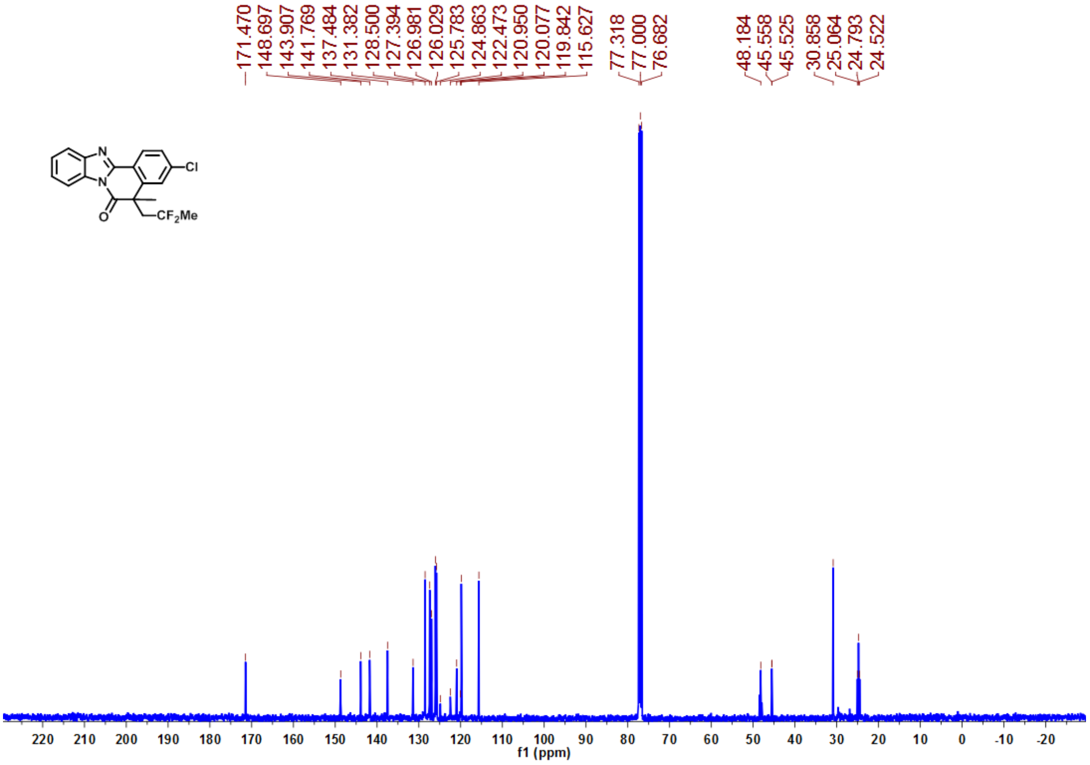

3d-^{19}F NMR (471 MHz, $CDCl_3$)

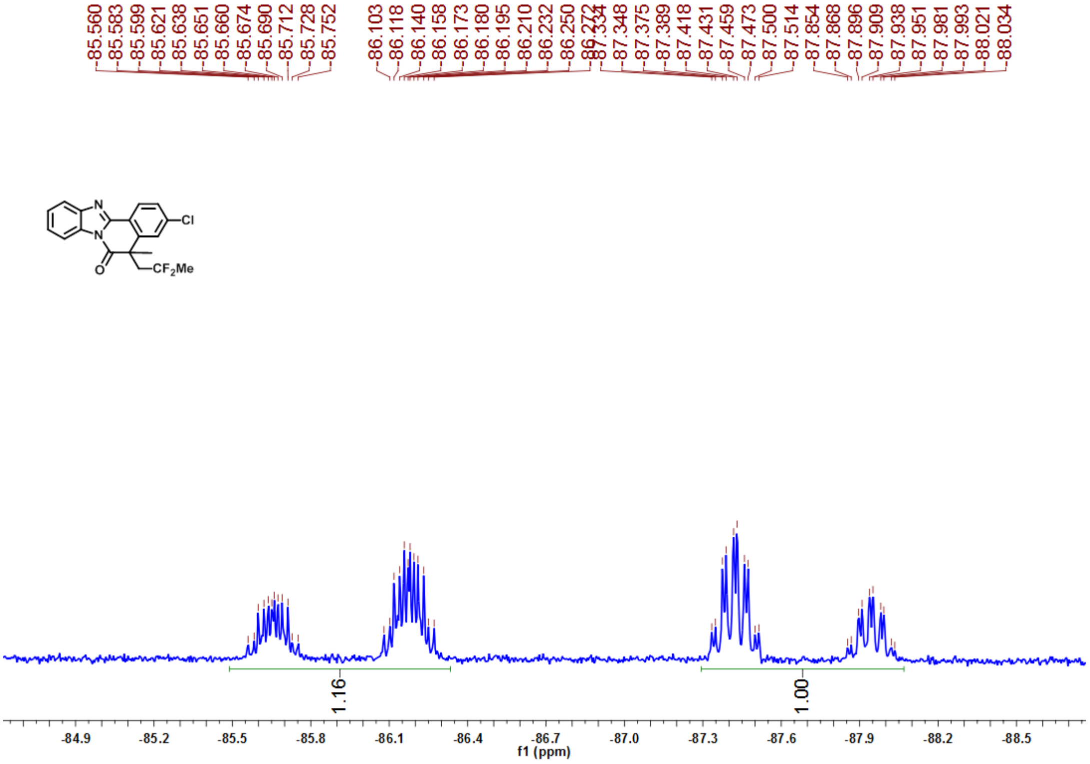

3e-^{1}H NMR (500 MHz, $CDCl_3$)

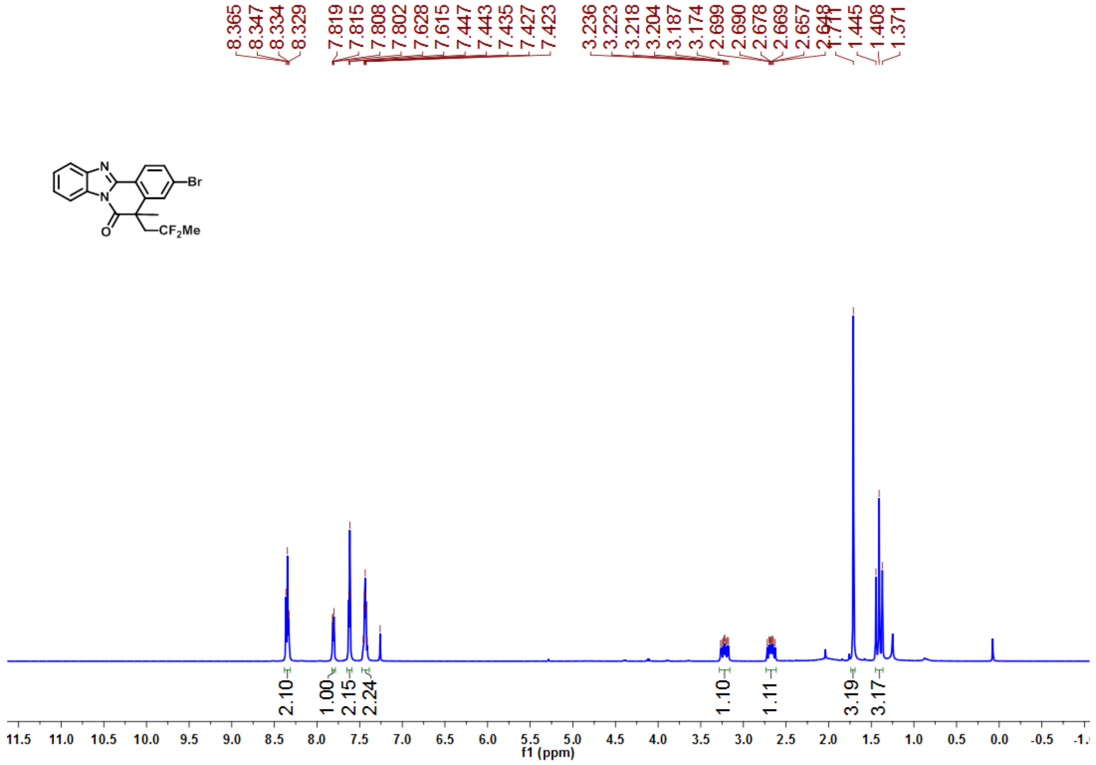

3e-^{13}C{^{1}H} NMR (125 MHz, $CDCl_3$)

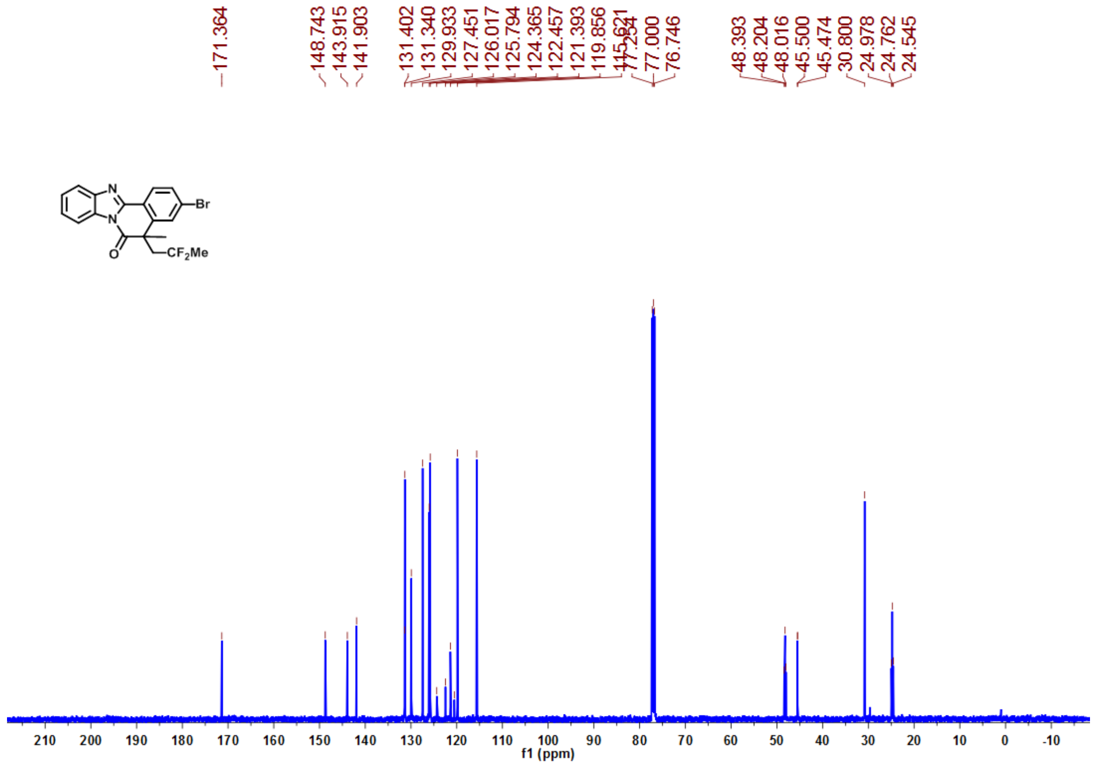

3e-^{19}F NMR (471 MHz, $CDCl_3$)

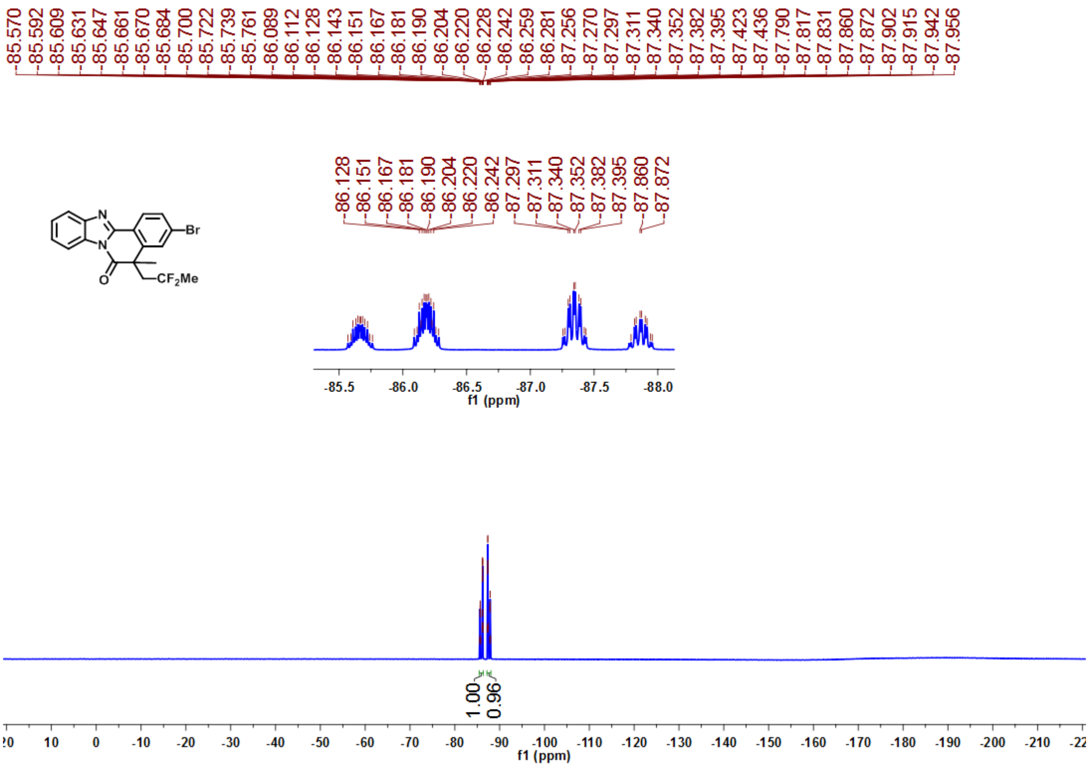

3f-^{1}H NMR (500 MHz, $CDCl_3$)

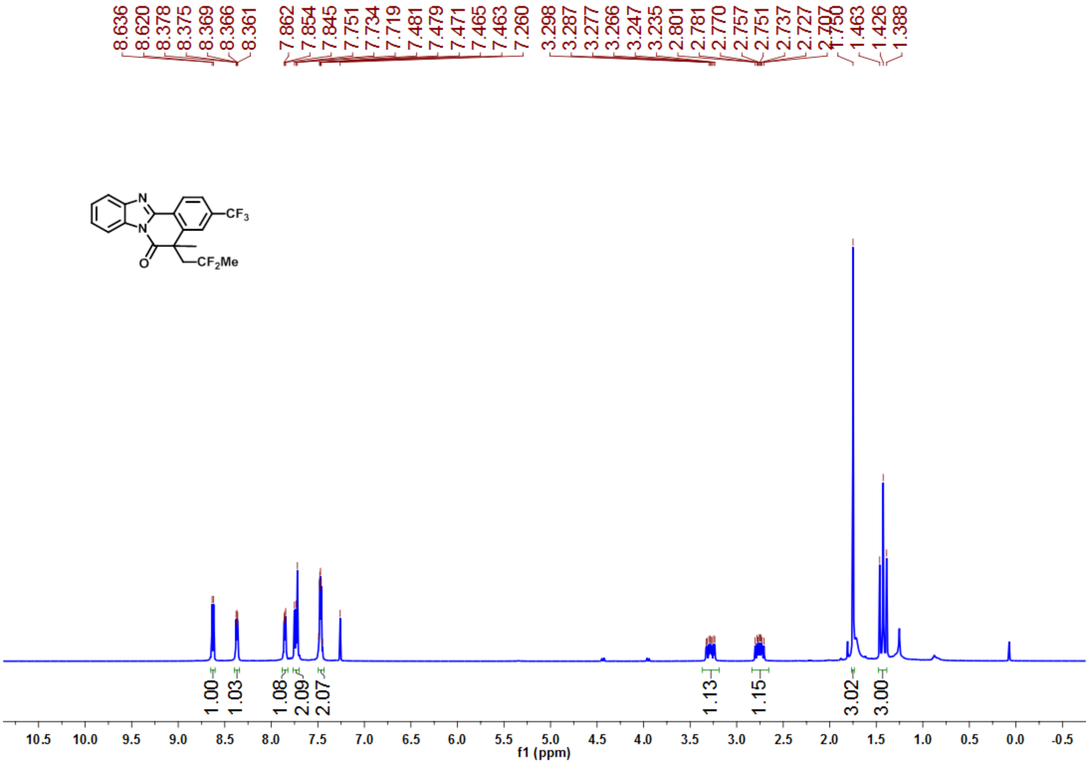

3f-$^{13}C\{^{1}H\}$ NMR (100 MHz, $CDCl_3$)

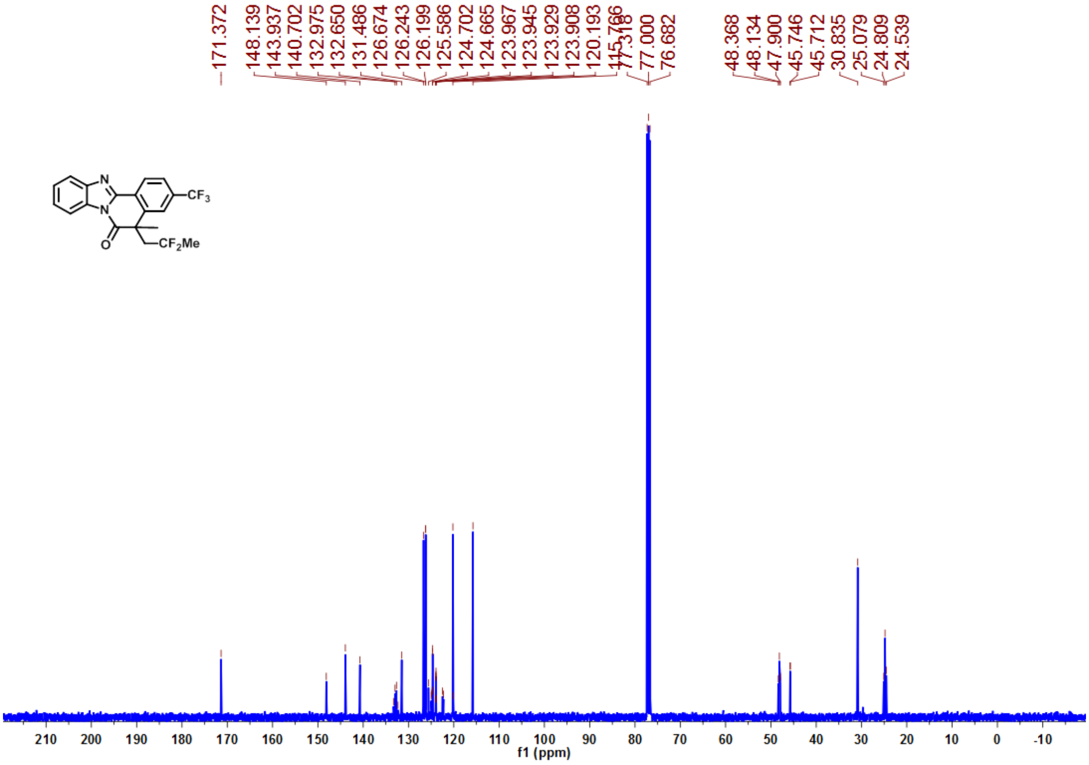

3f-^{19}F NMR (471 MHz, $CDCl_3$)

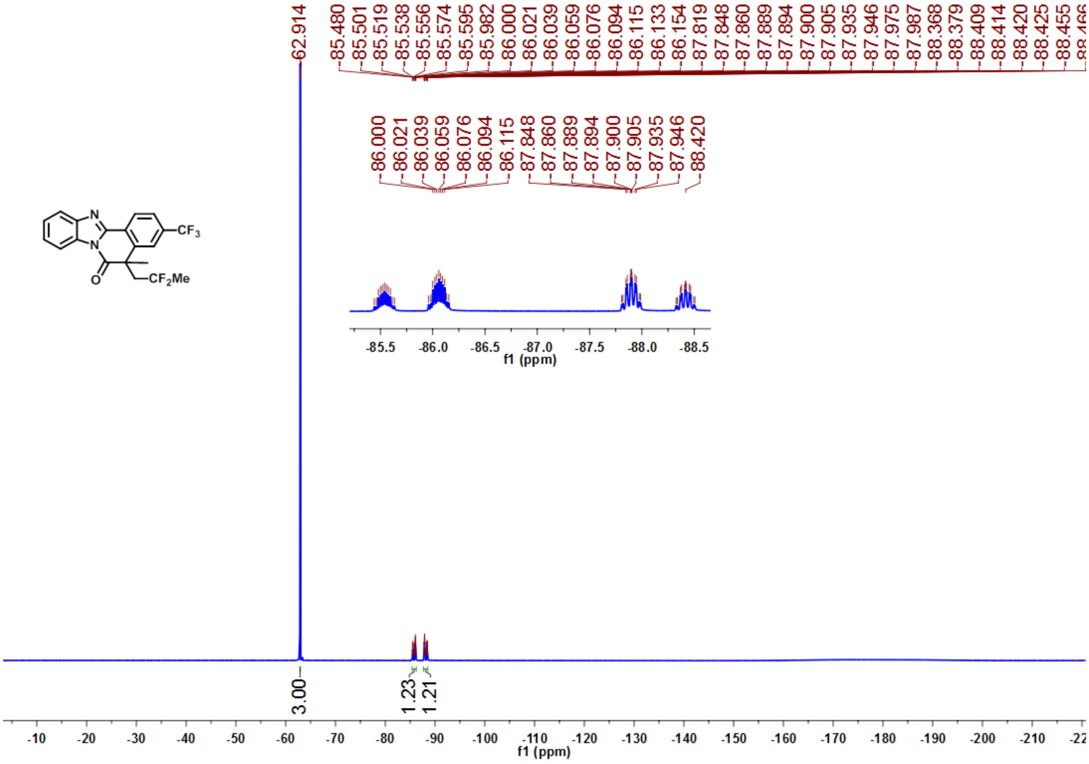

3g-^{1}H NMR (400 MHz, $CDCl_3$)

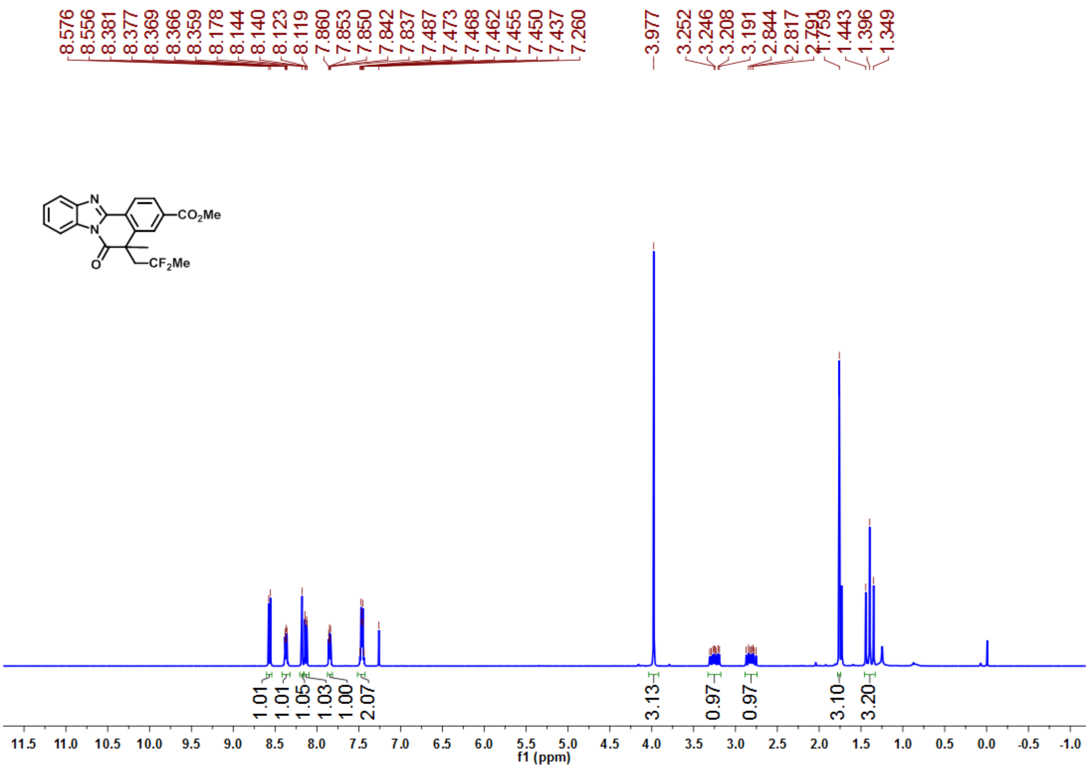

3g-^{13}C{^{1}H} NMR (125 MHz, $CDCl_3$)

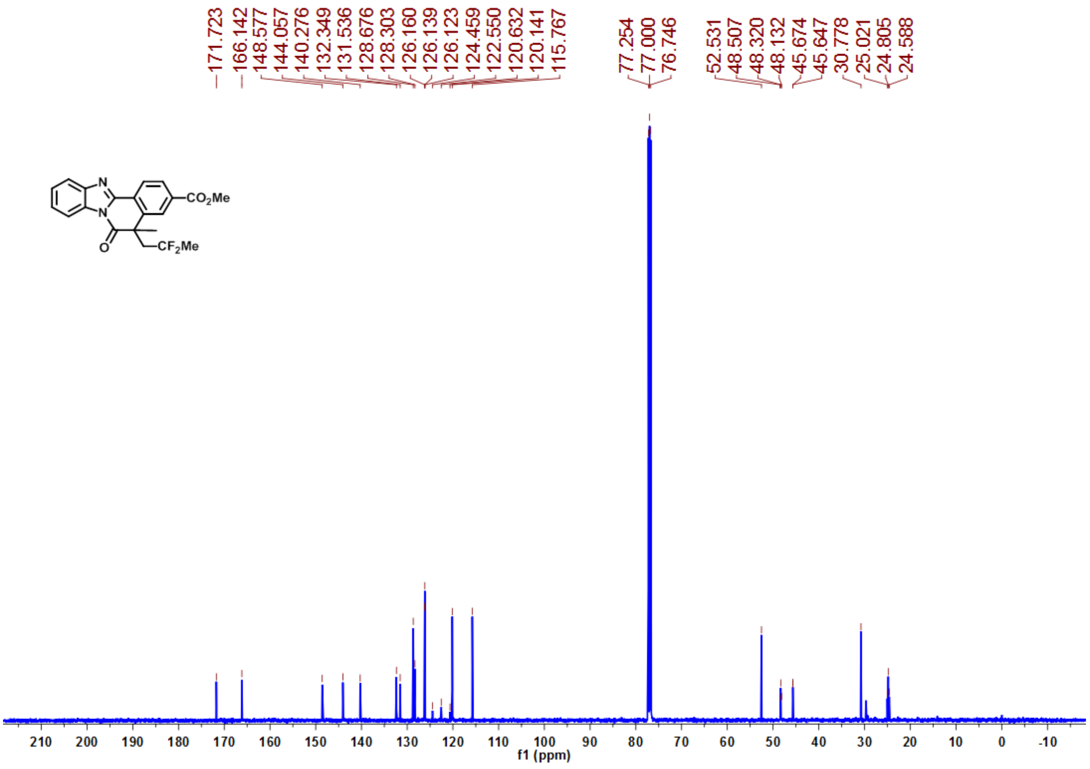

3g-^{19}F NMR (471 MHz, $CDCl_3$)

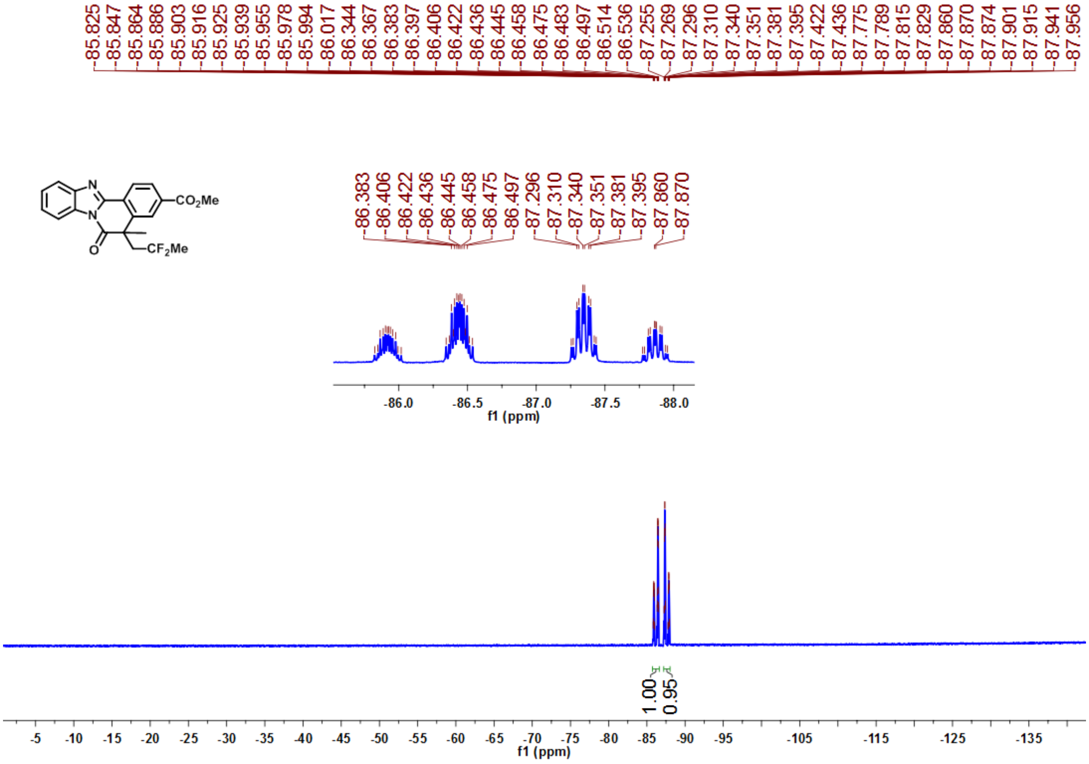

3h-^{1}H NMR (400 MHz, $CDCl_3$)

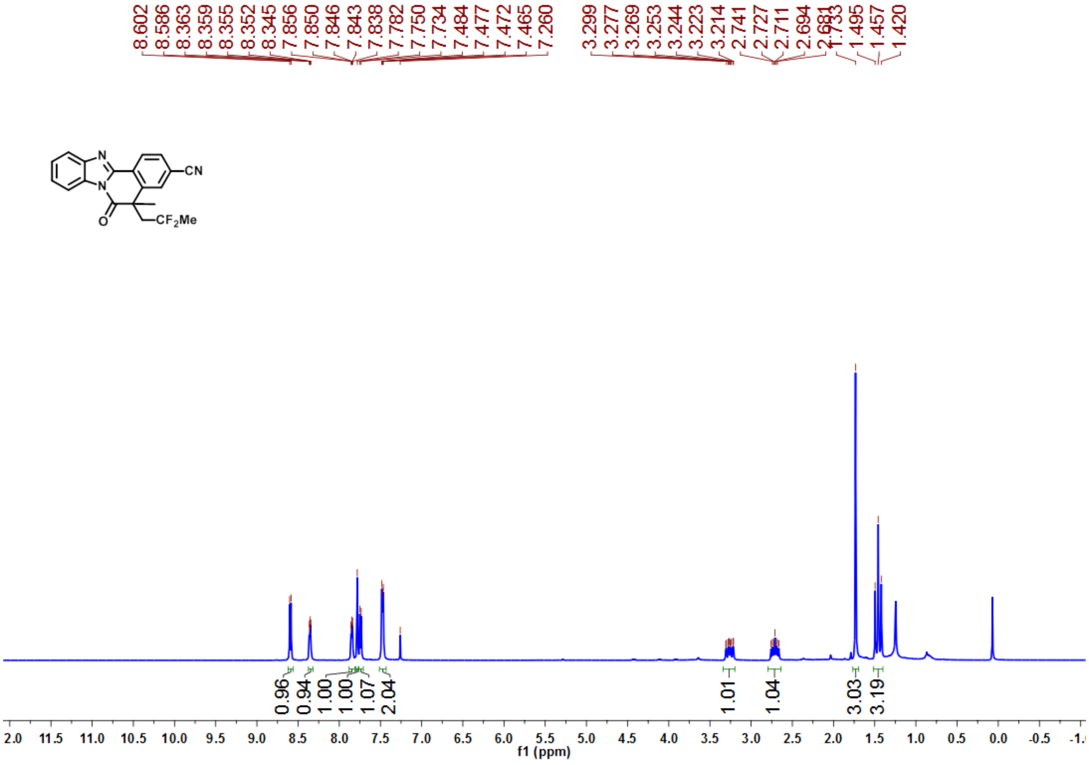

3h-$^{13}C\{^{1}H\}$ NMR (100 MHz, $CDCl_3$)

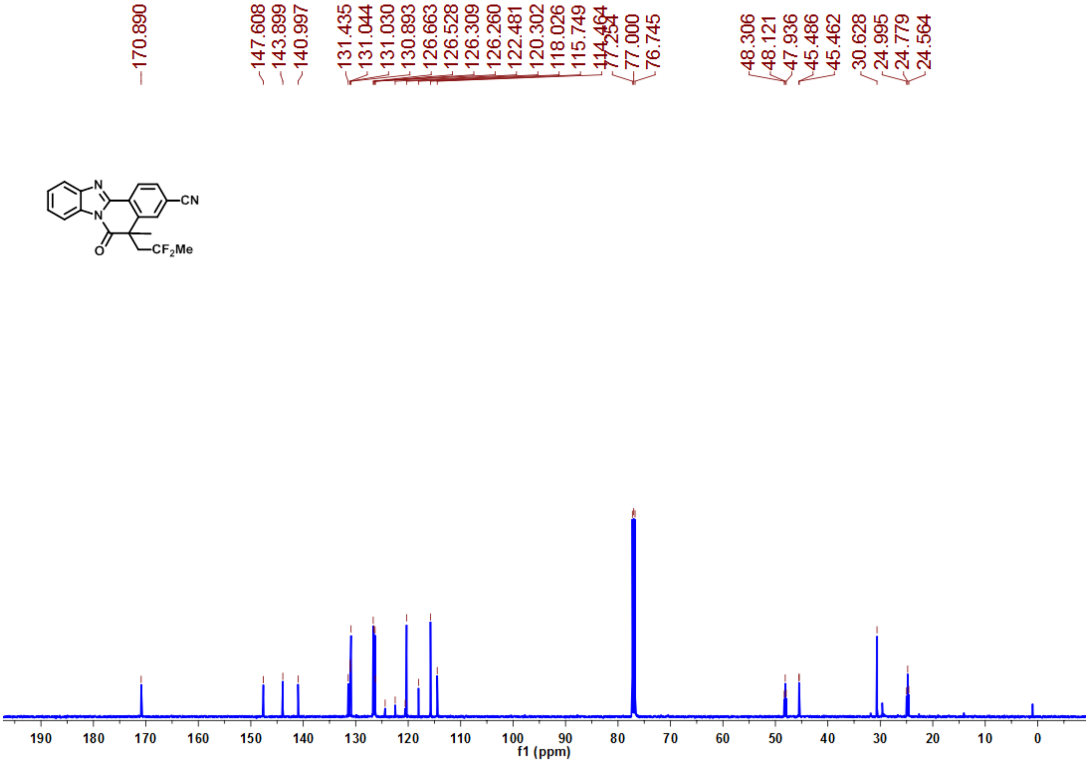

3h-^{19}F NMR (471 MHz, $CDCl_3$)

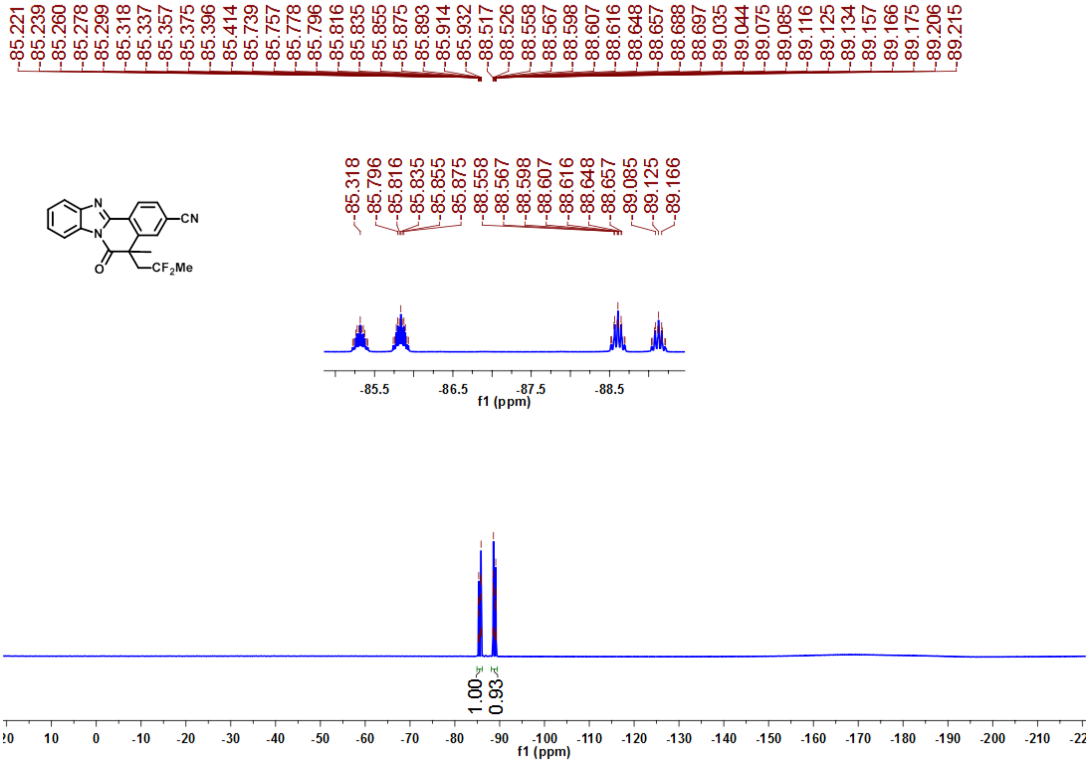

3i-^{1}H NMR (400 MHz, $CDCl_3$)

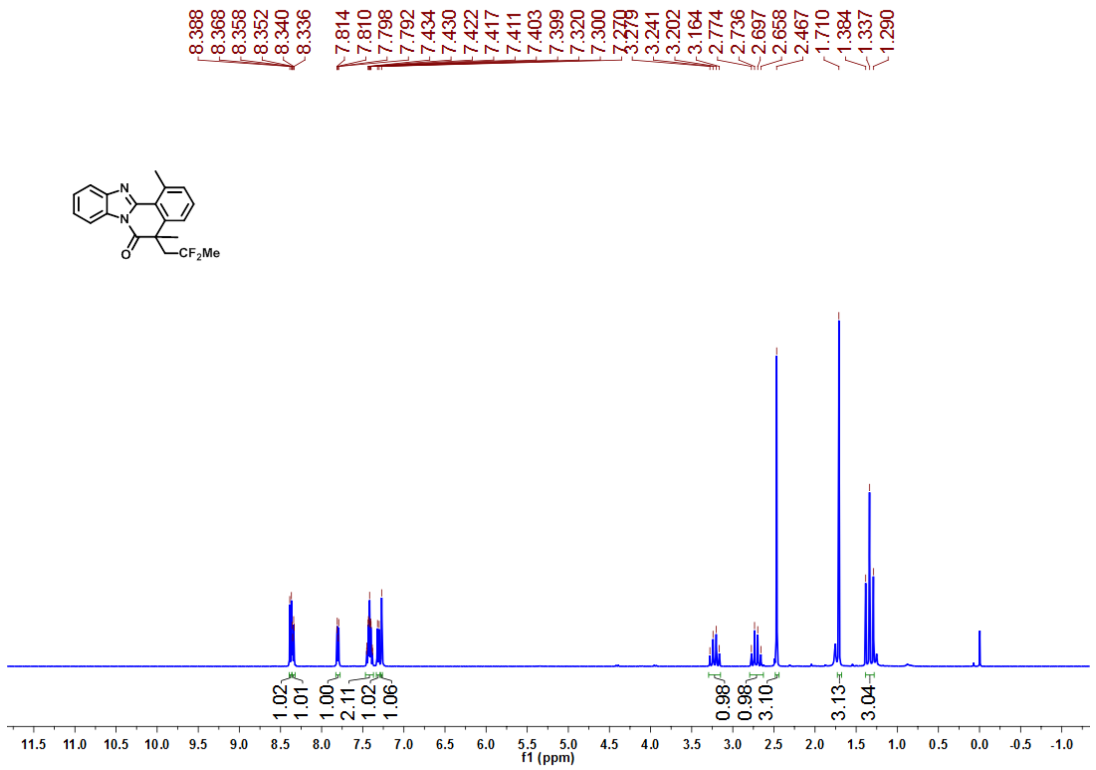

3i-^{13}C{^{1}H} NMR (100 MHz, $CDCl_3$)

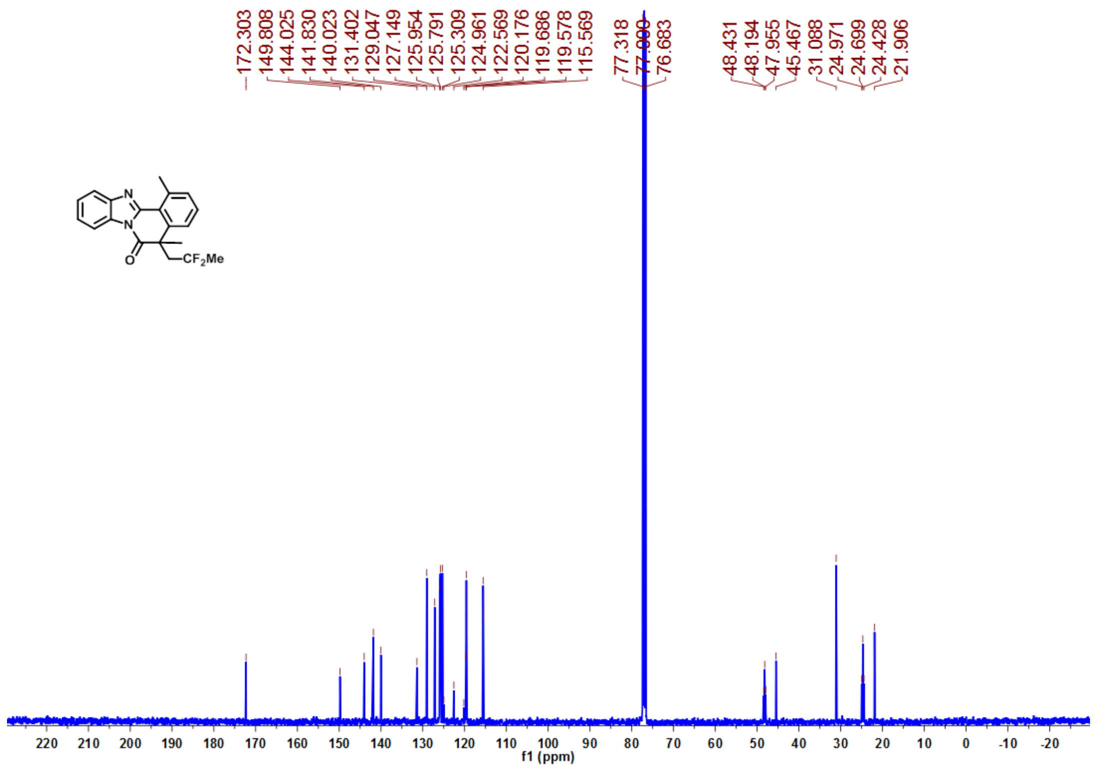

3i-^{19}F NMR (471 MHz, $CDCl_3$)

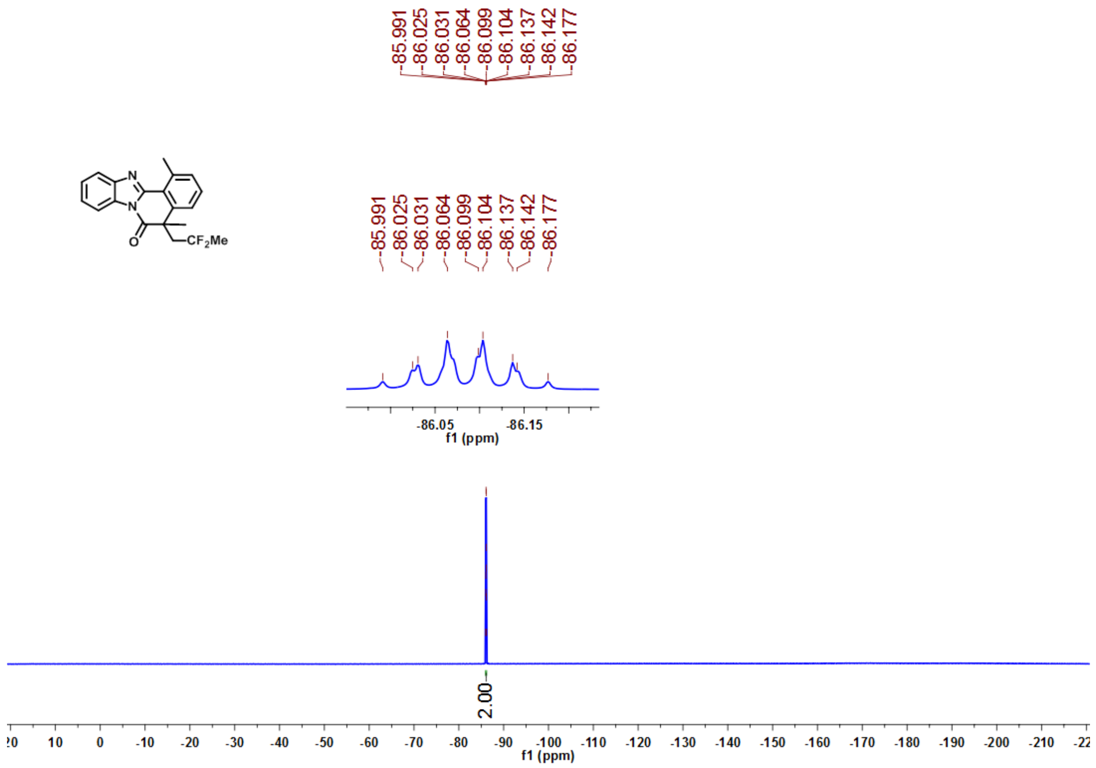

3j-^{1}H NMR (500 MHz, $CDCl_3$)

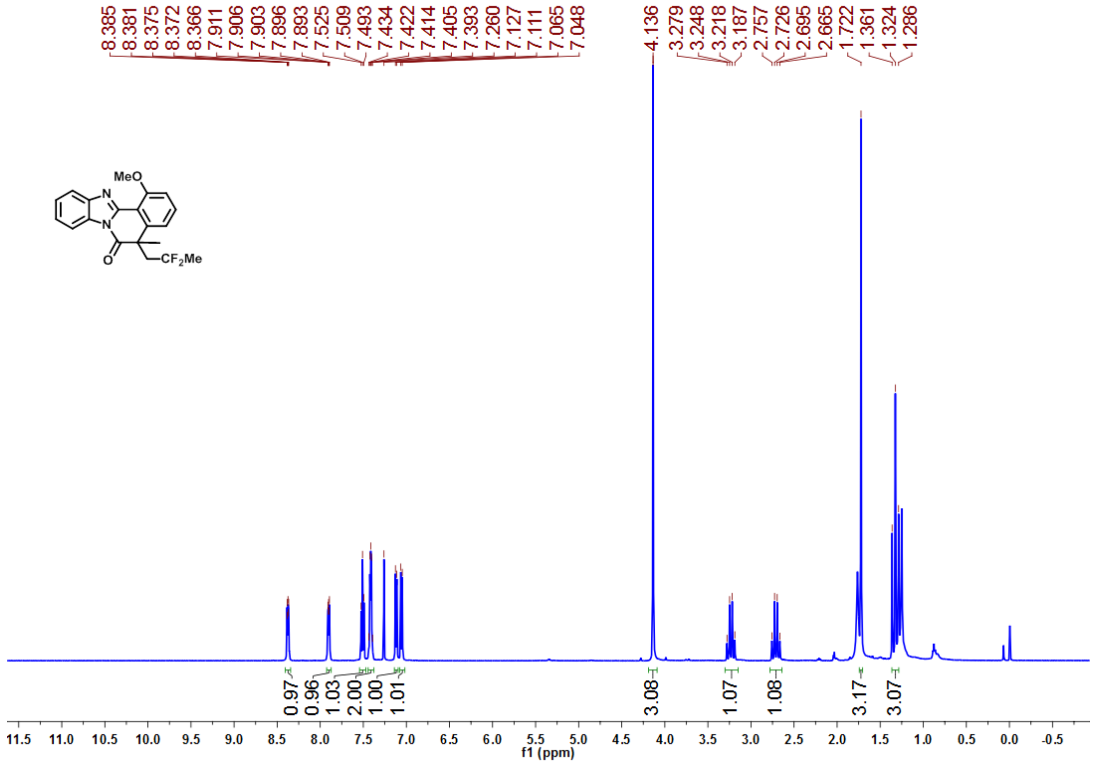

3j-$^{13}C\{^{1}H\}$ NMR (100 MHz, $CDCl_3$)

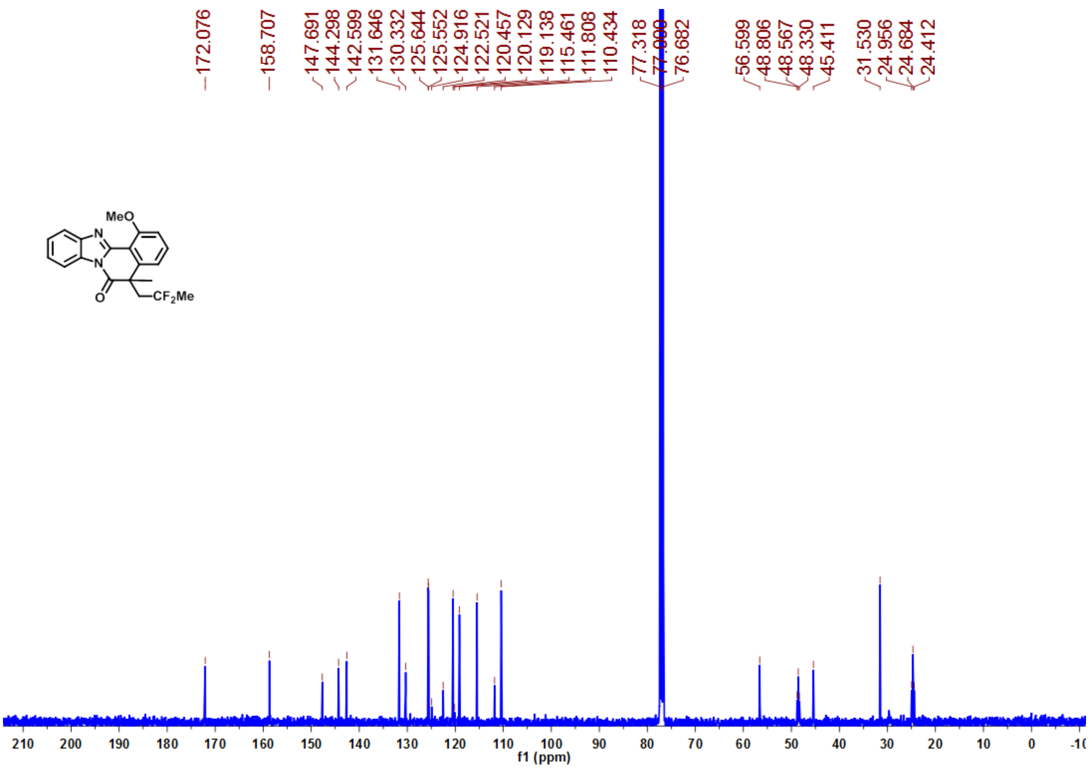

3j-^{19}F NMR (471 MHz, $CDCl_3$)

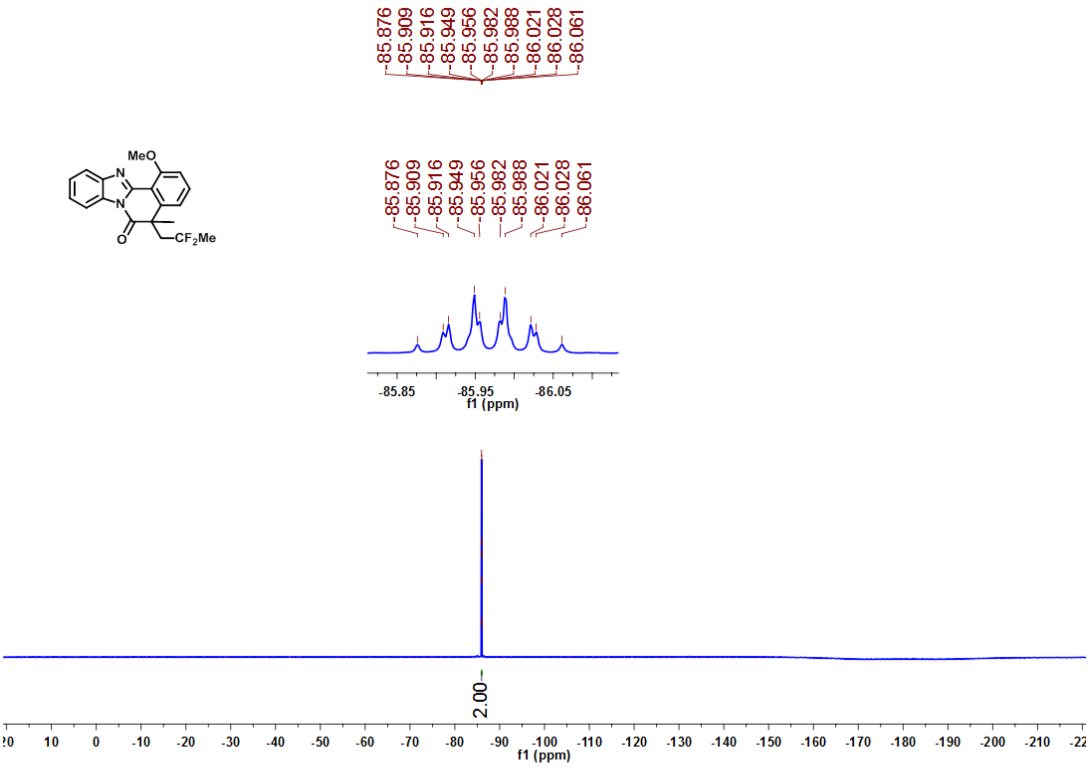

3k-^{1}H NMR (500 MHz, $CDCl_3$)

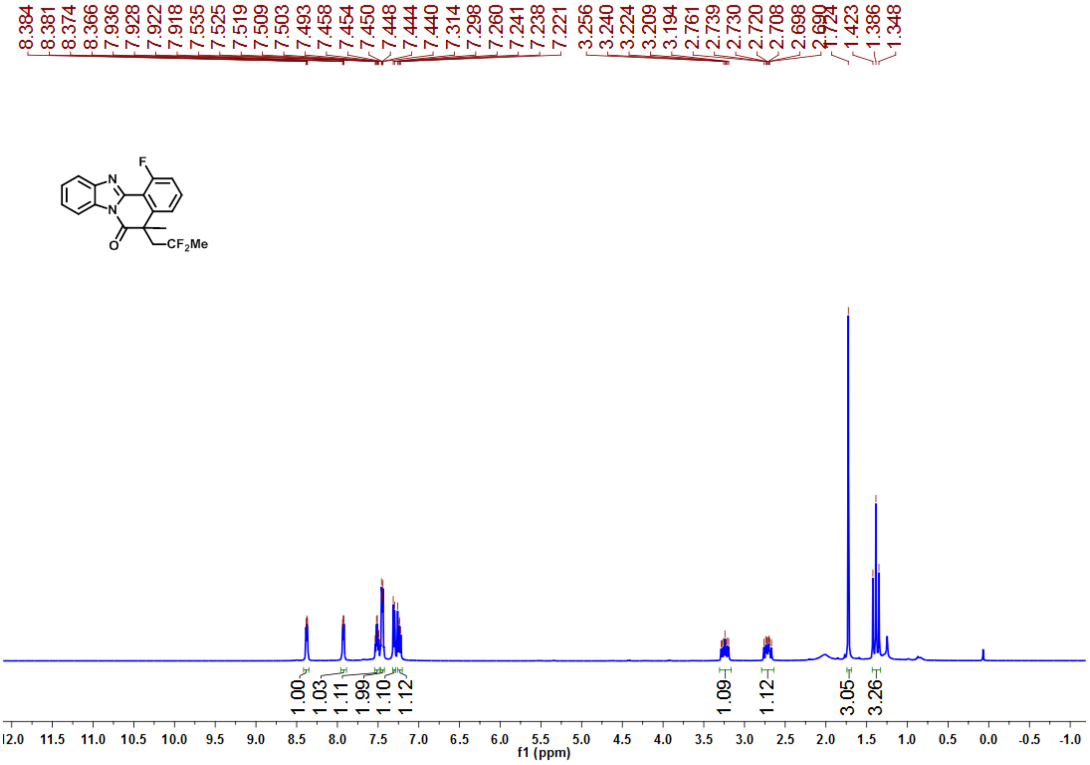

3k-$^{13}C\{^{1}H\}$ NMR (100 MHz, $CDCl_3$)

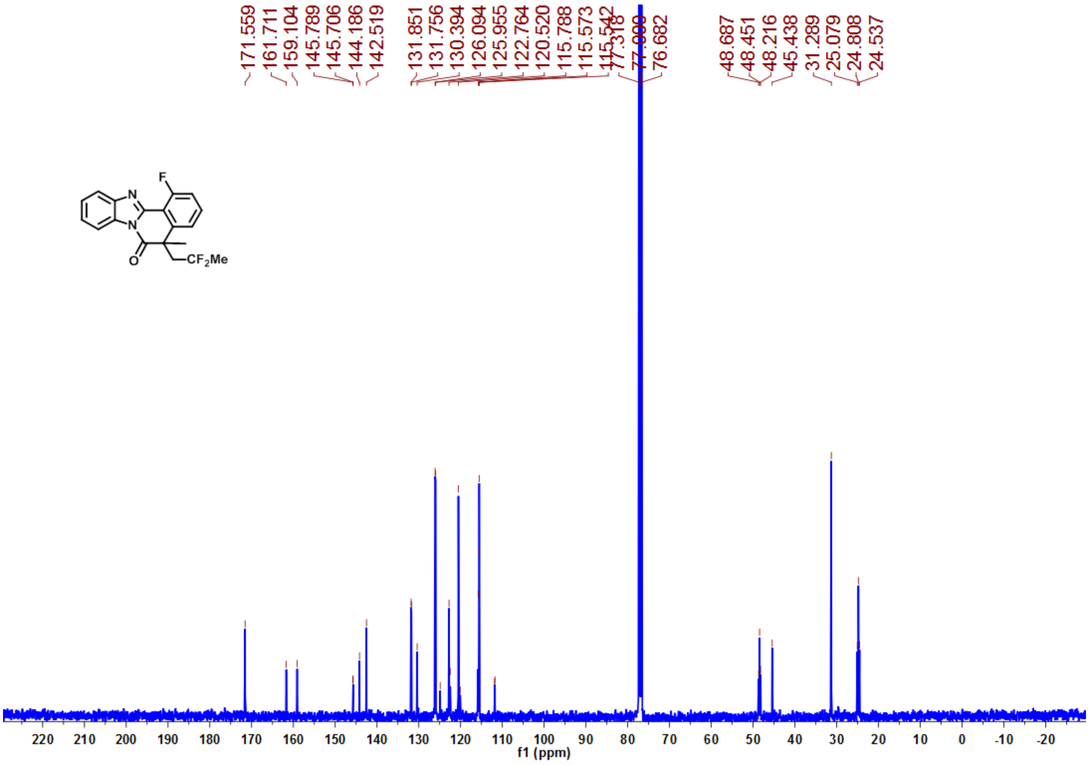

3k-^{19}F NMR (471 MHz, $CDCl_3$)

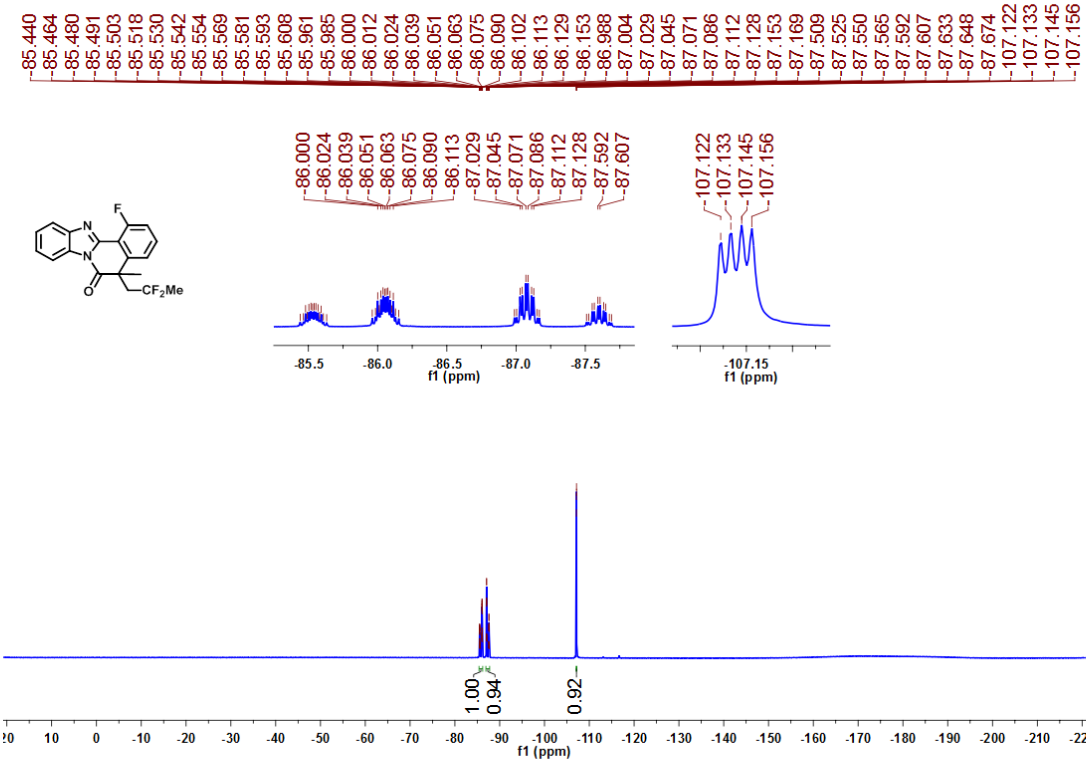

3l-^{1}H NMR (400 MHz, $CDCl_3$)

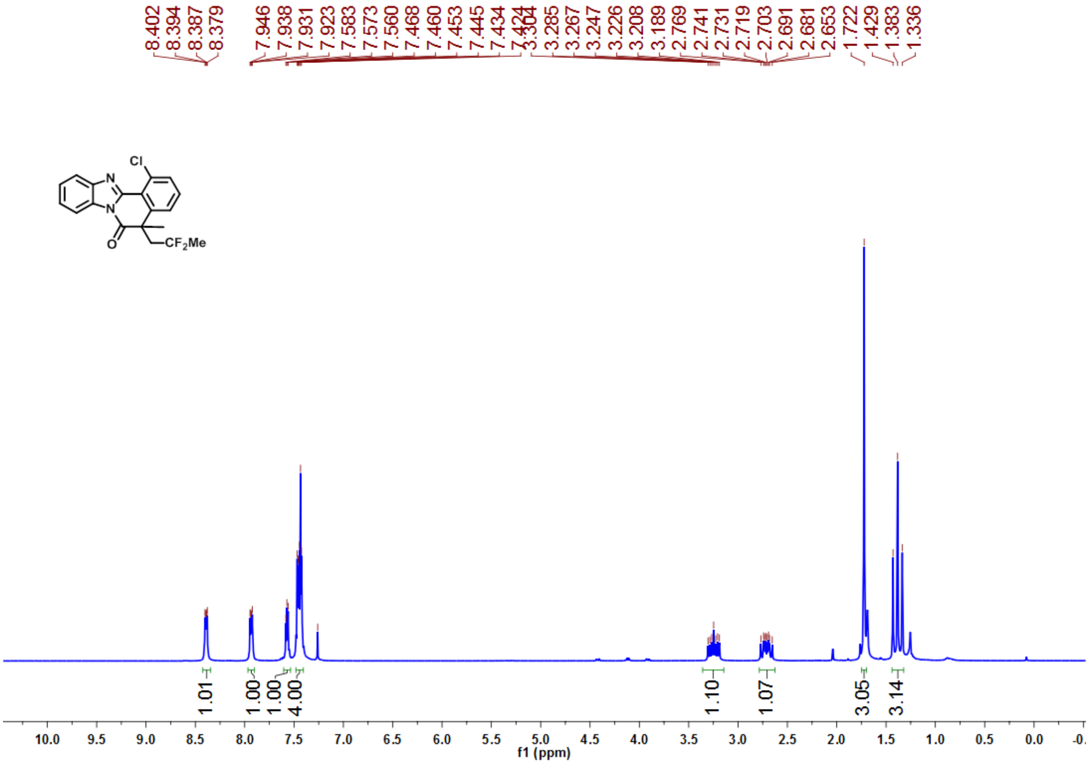

3l-$^{13}C\{^1H\}$ NMR (100 MHz, $CDCl_3$)

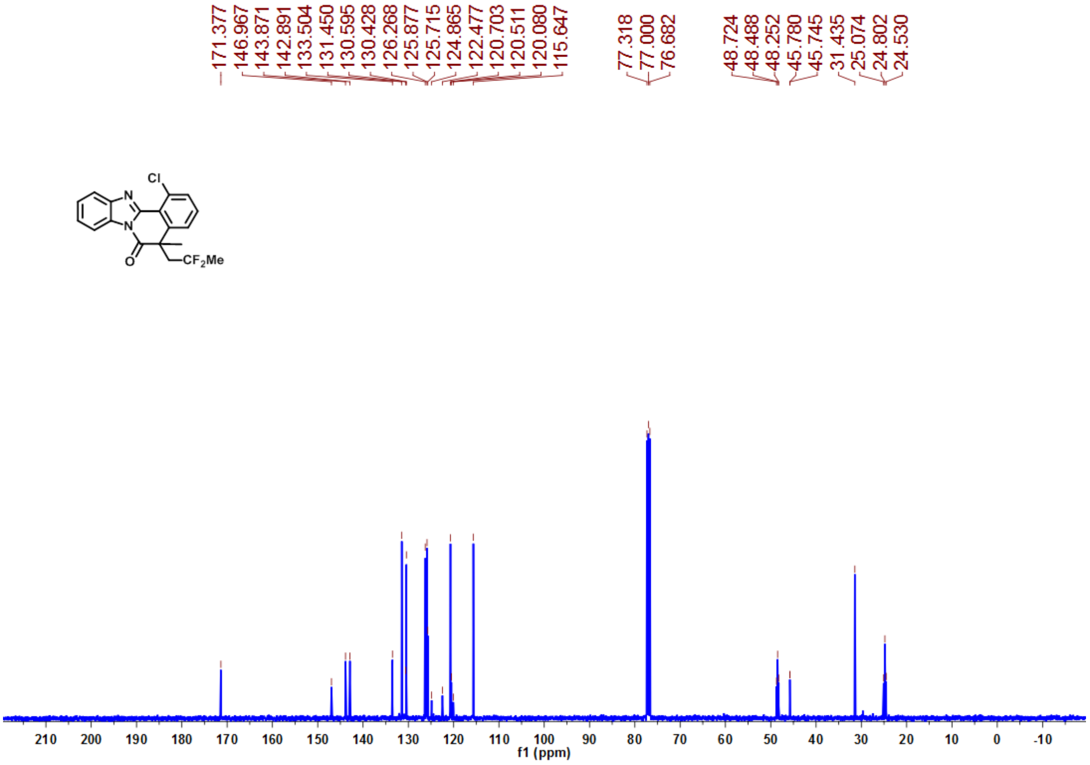

3l-^{19}F NMR (471 MHz, $CDCl_3$)

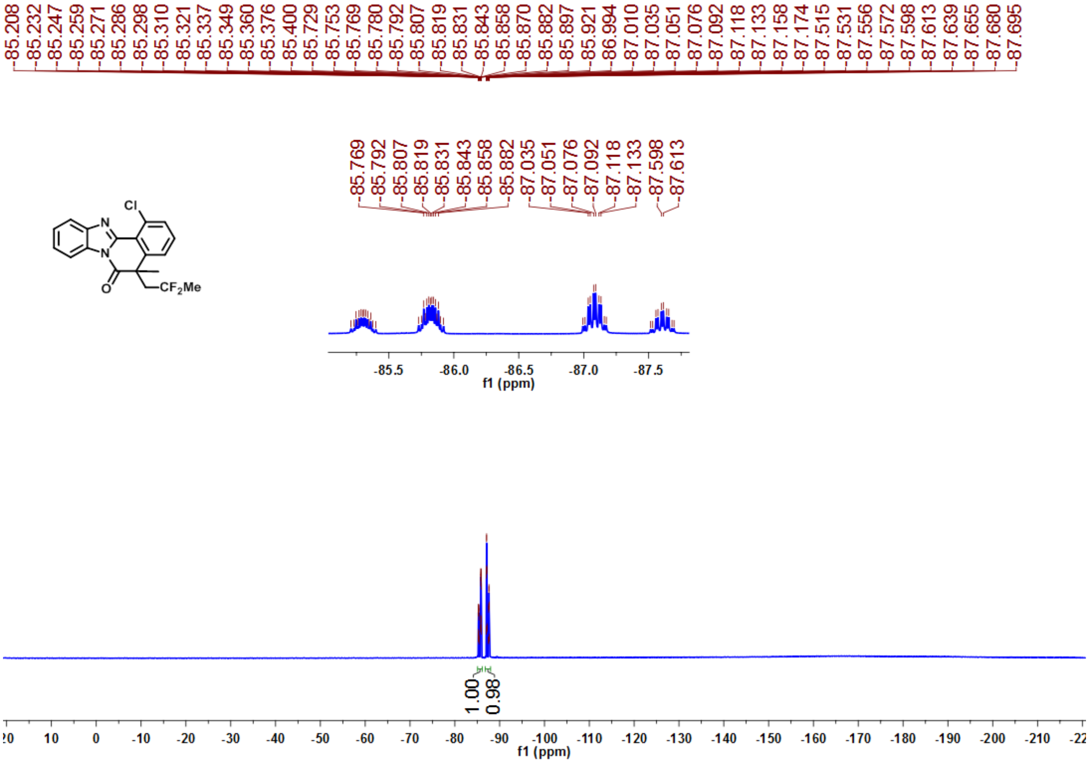

3m-^{1}H NMR (500 MHz, $CDCl_3$)

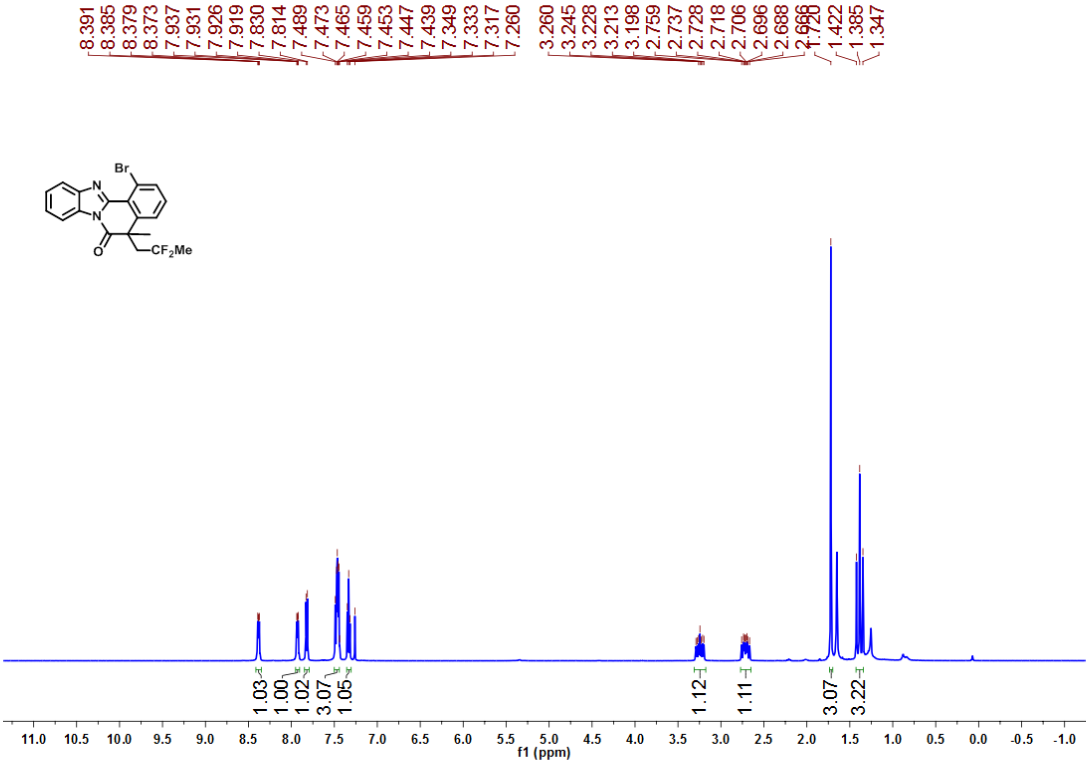

3m-^{13}C{^{1}H} NMR (100 MHz, $CDCl_3$)

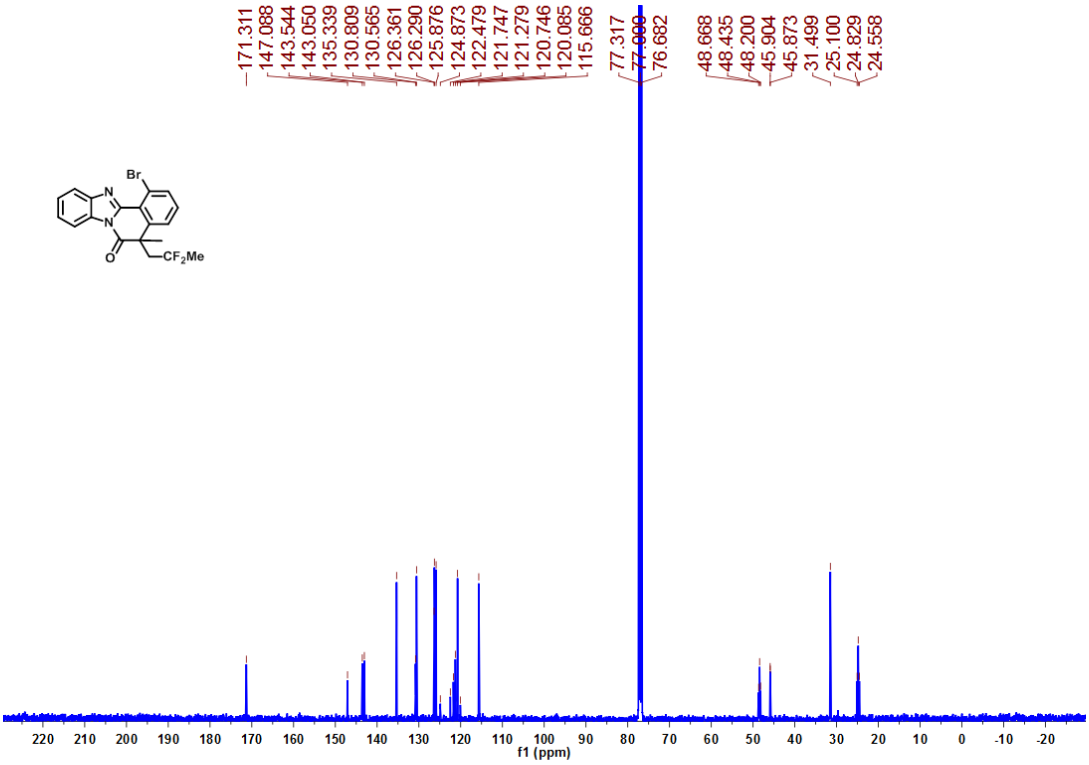

3m-^{19}F NMR (471 MHz, $CDCl_3$)

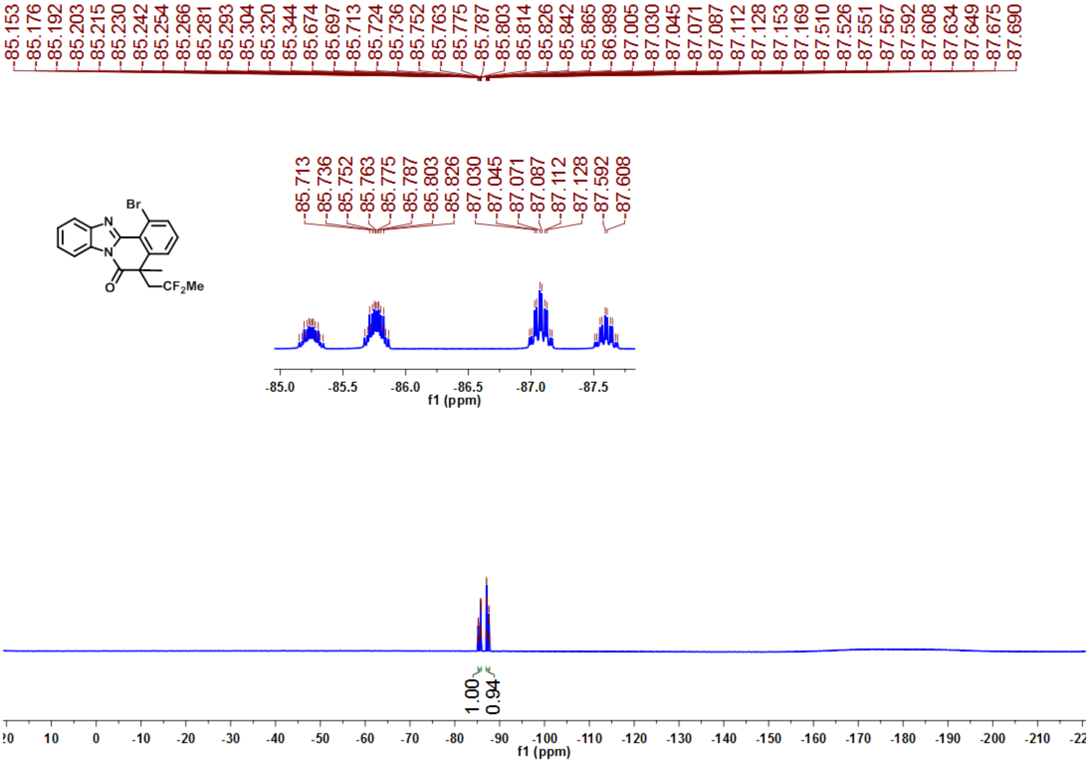

3n-^{1}H NMR (500 MHz, $CDCl_3$)

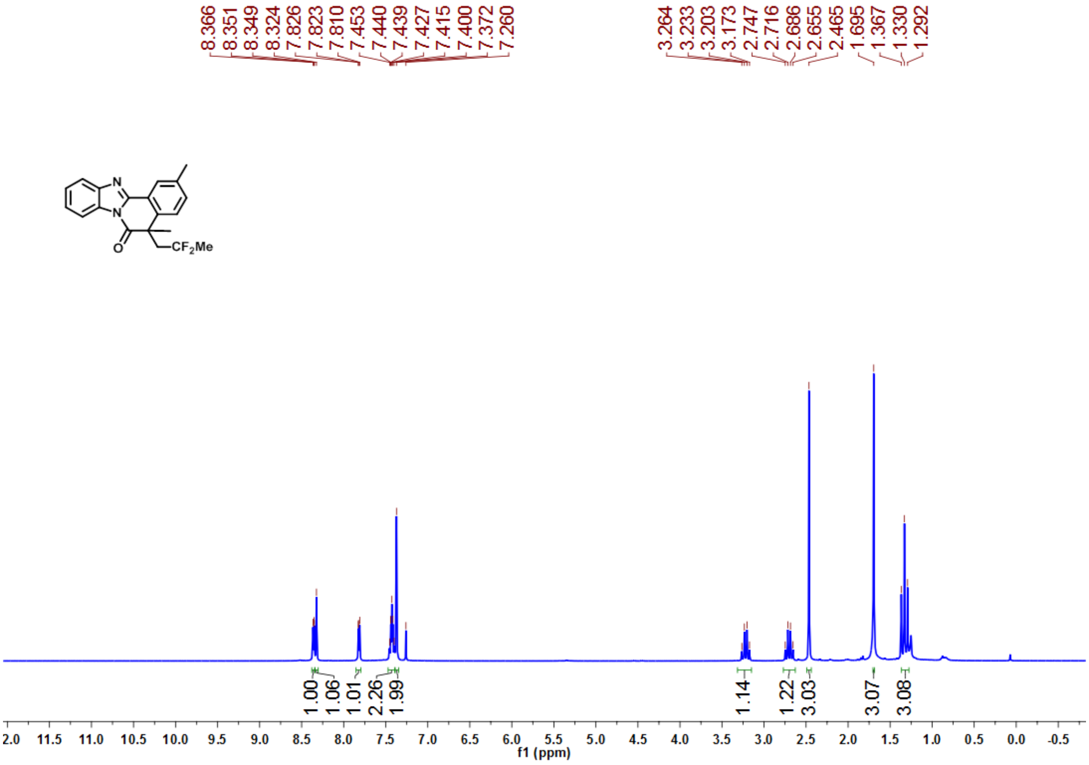

3n-$^{13}C\{^{1}H\}$ NMR (100 MHz, $CDCl_3$)

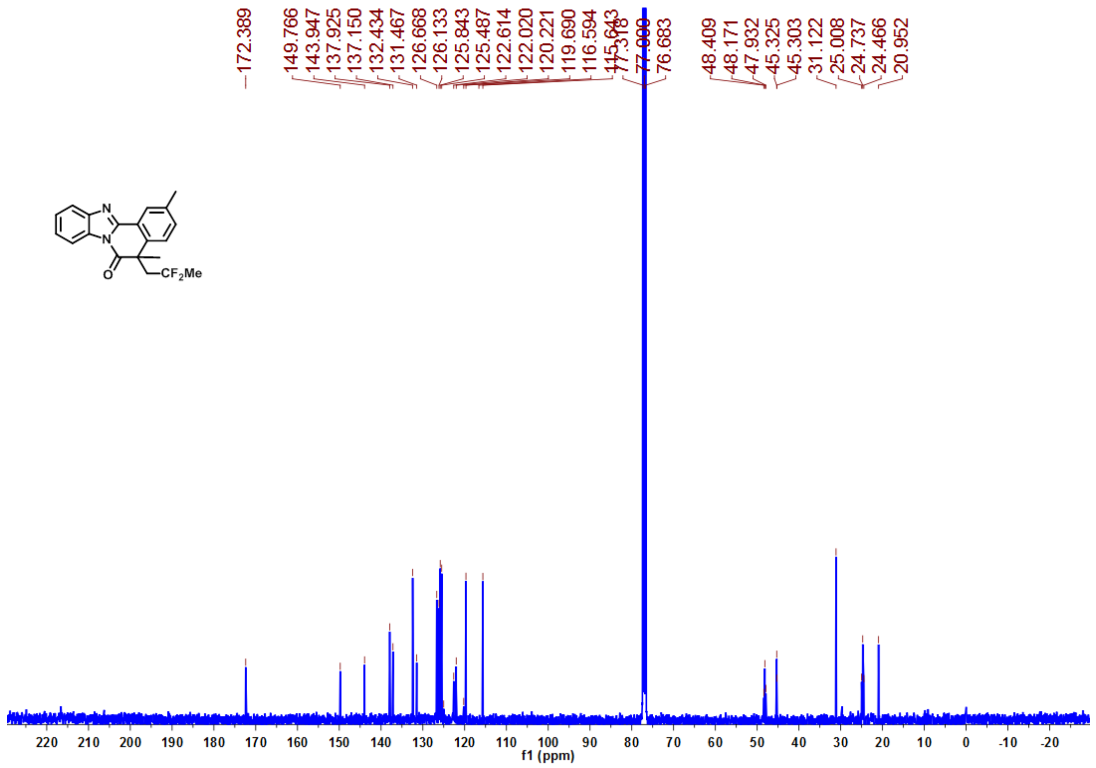

3n-^{19}F NMR (471 MHz, $CDCl_3$)

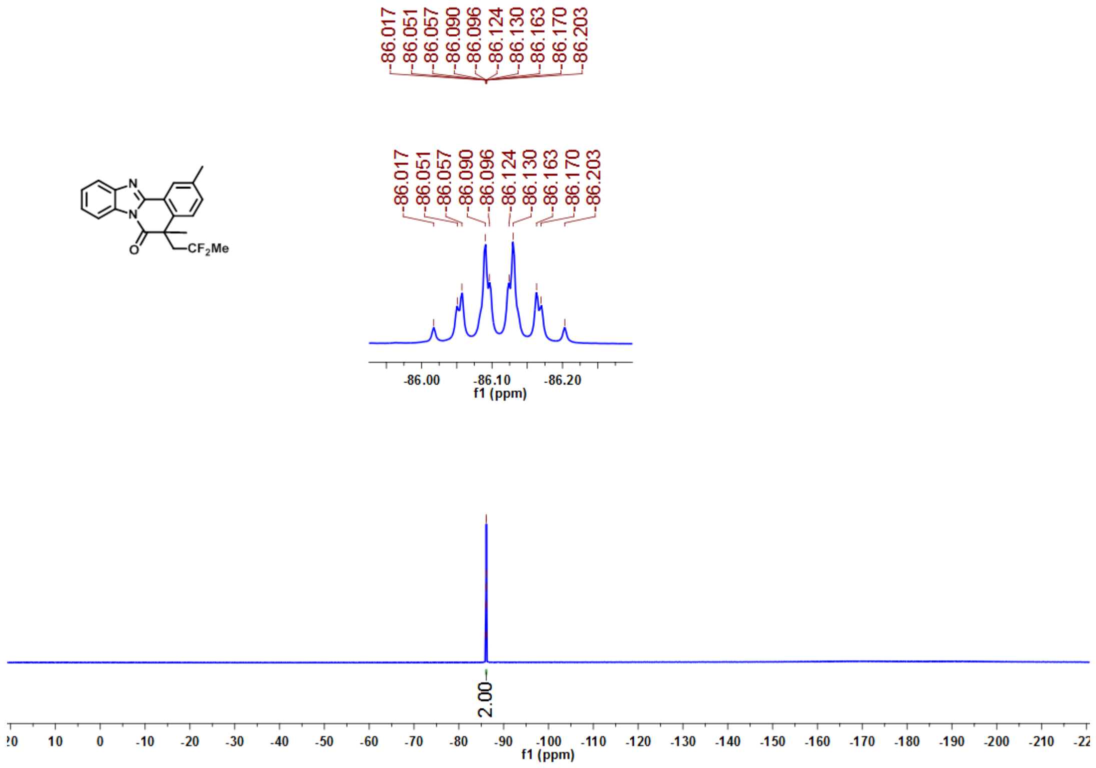

3o-^{1}H NMR (500 MHz, $CDCl_3$)

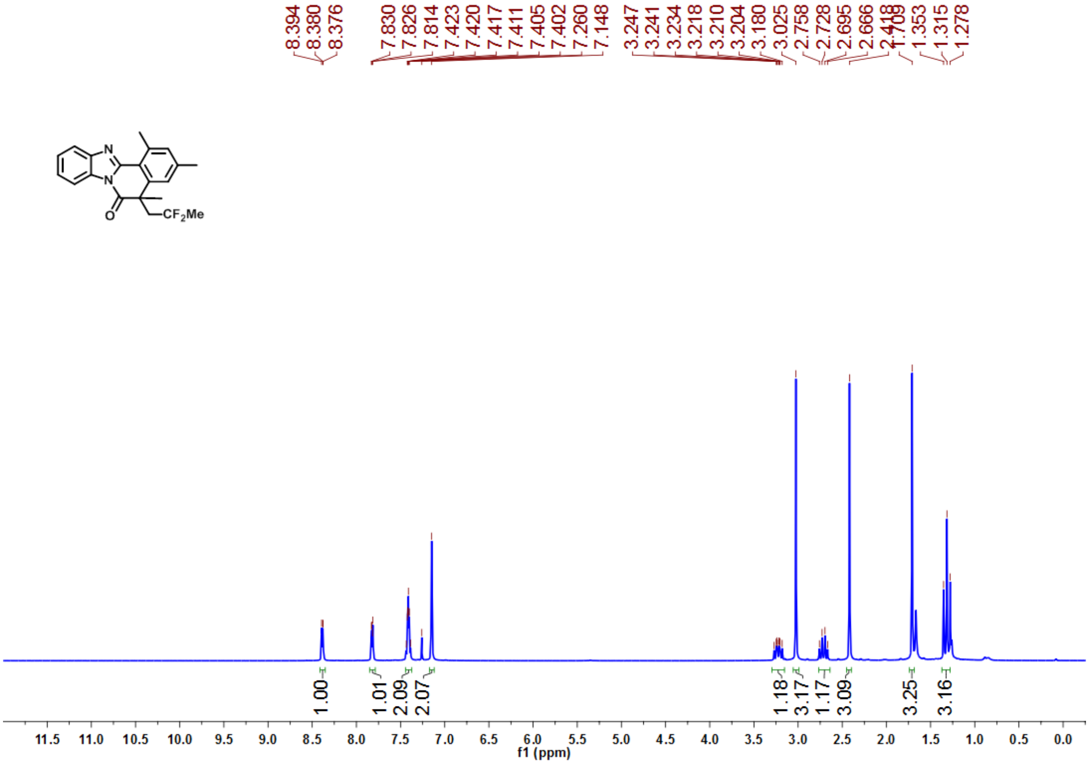

3o-^{13}C{^{1}H} NMR (100 MHz, $CDCl_3$)

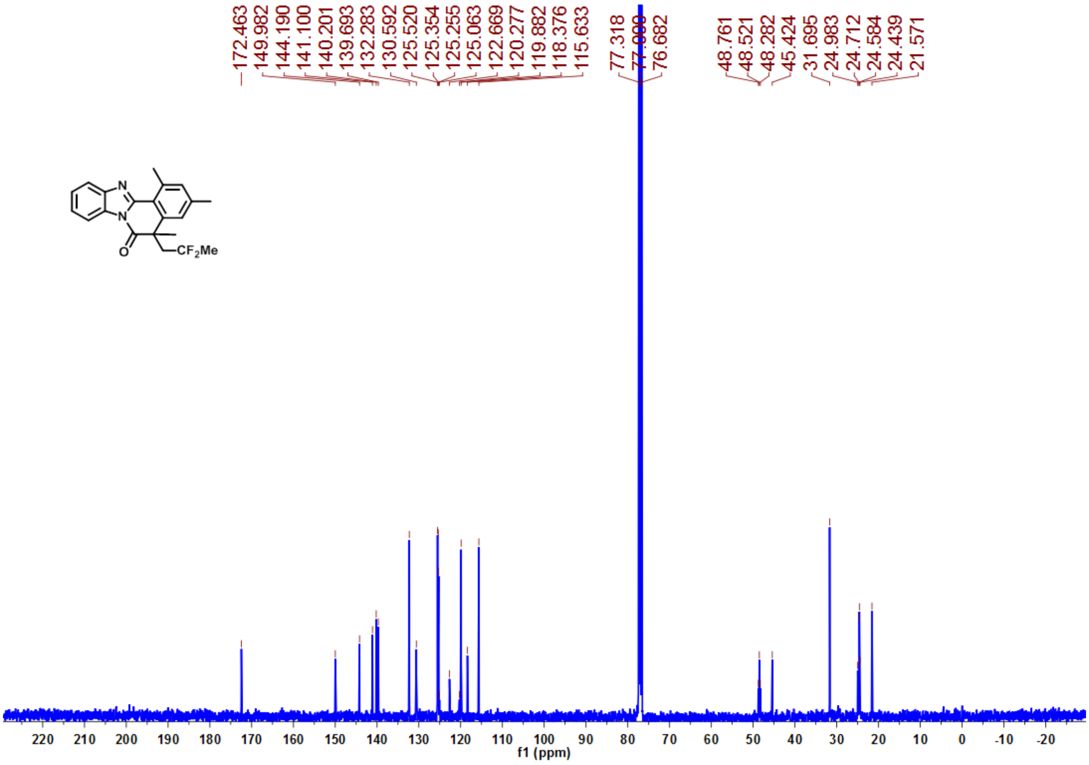

3o-^{19}F NMR (471 MHz, $CDCl_3$)

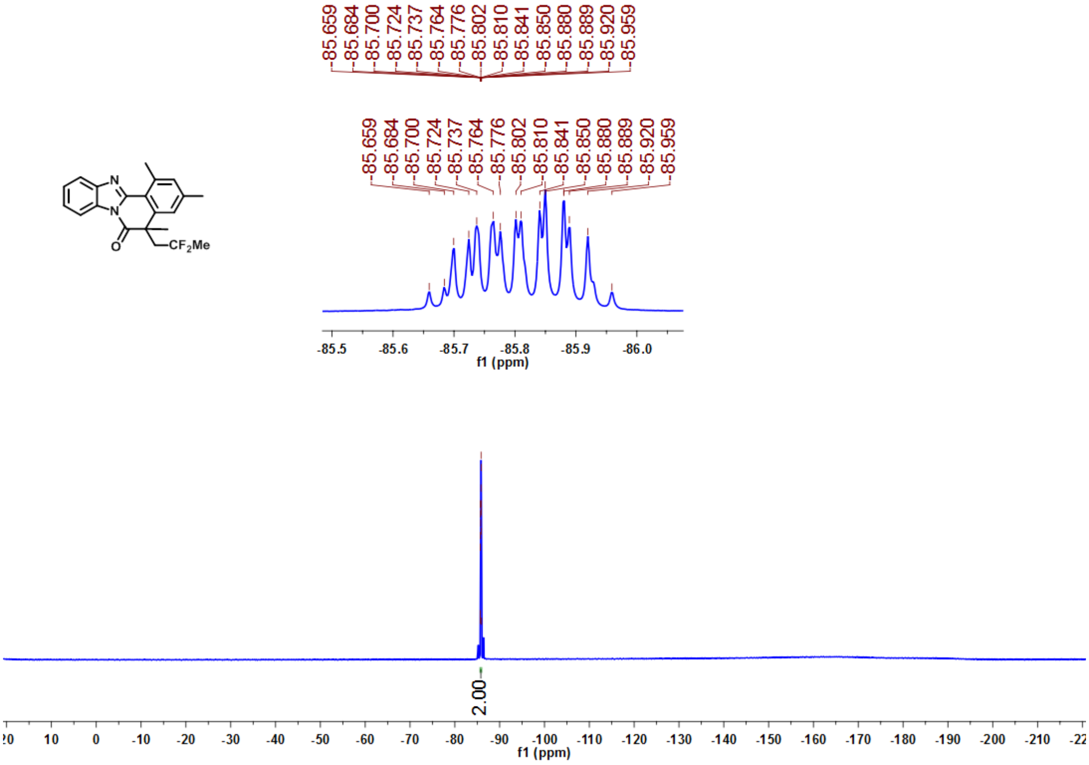

3p-^{1}H NMR (500 MHz, $CDCl_3$)

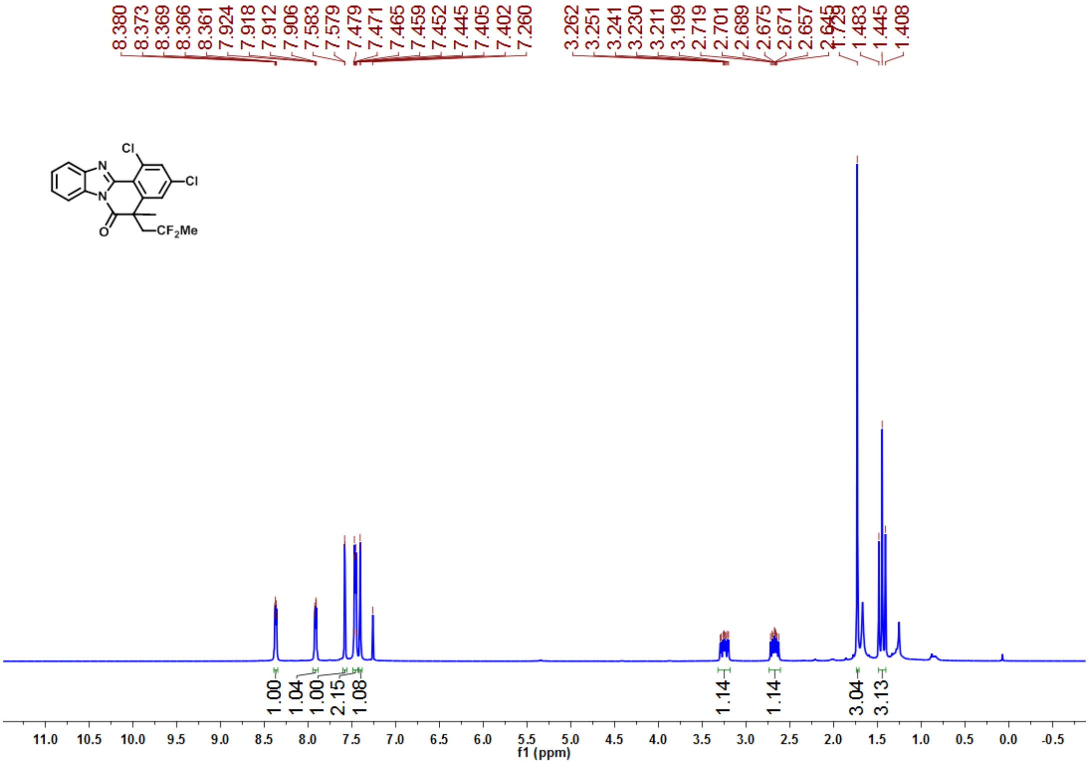

3p-$^{13}C\{^{1}H\}$ NMR (100 MHz, $CDCl_3$)

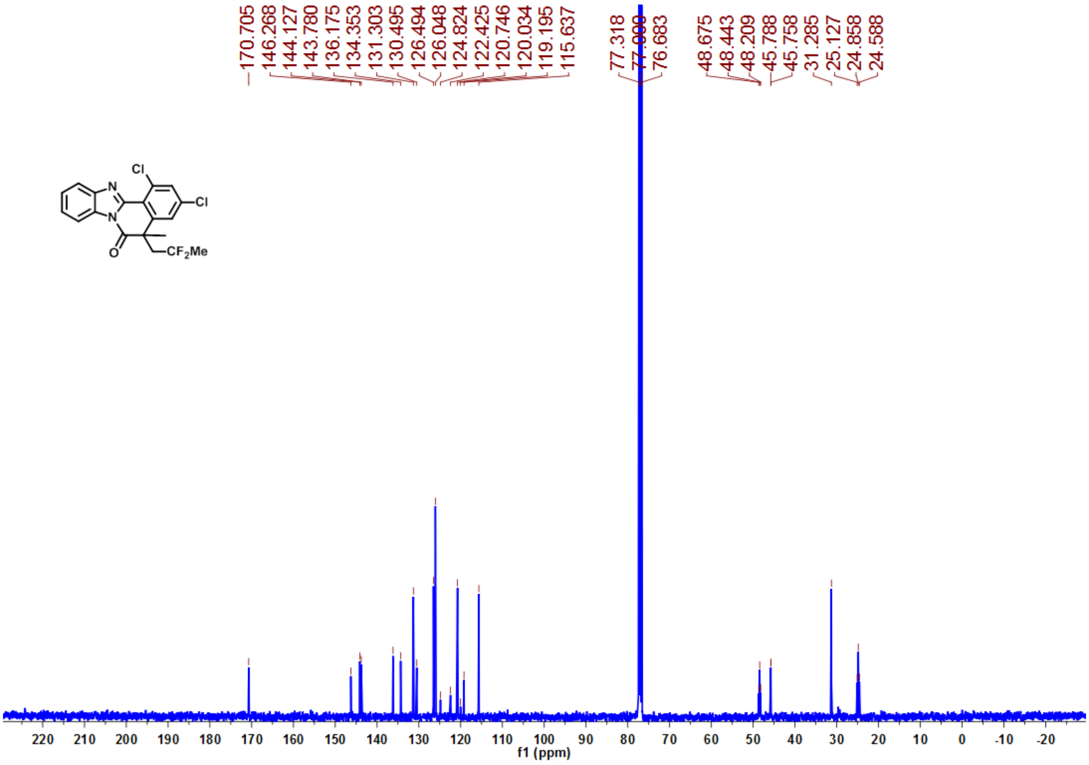

3p-^{19}F NMR (471 MHz, $CDCl_3$)

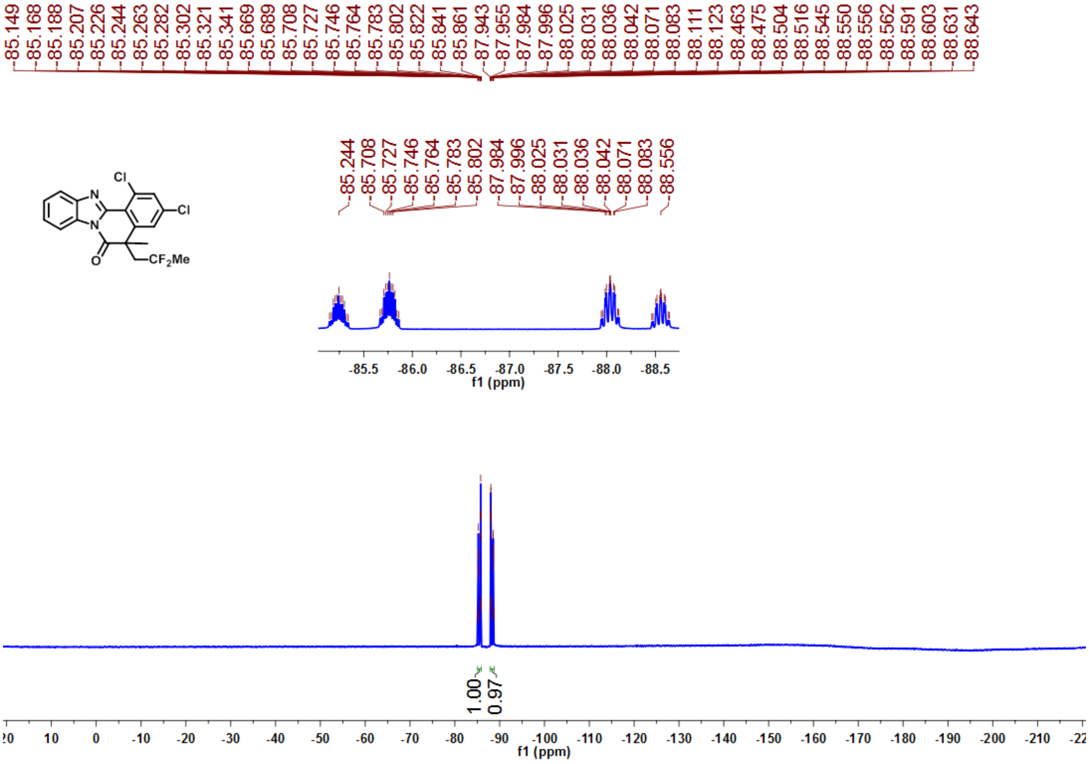

3q-^{1}H NMR (400 MHz, $CDCl_3$)

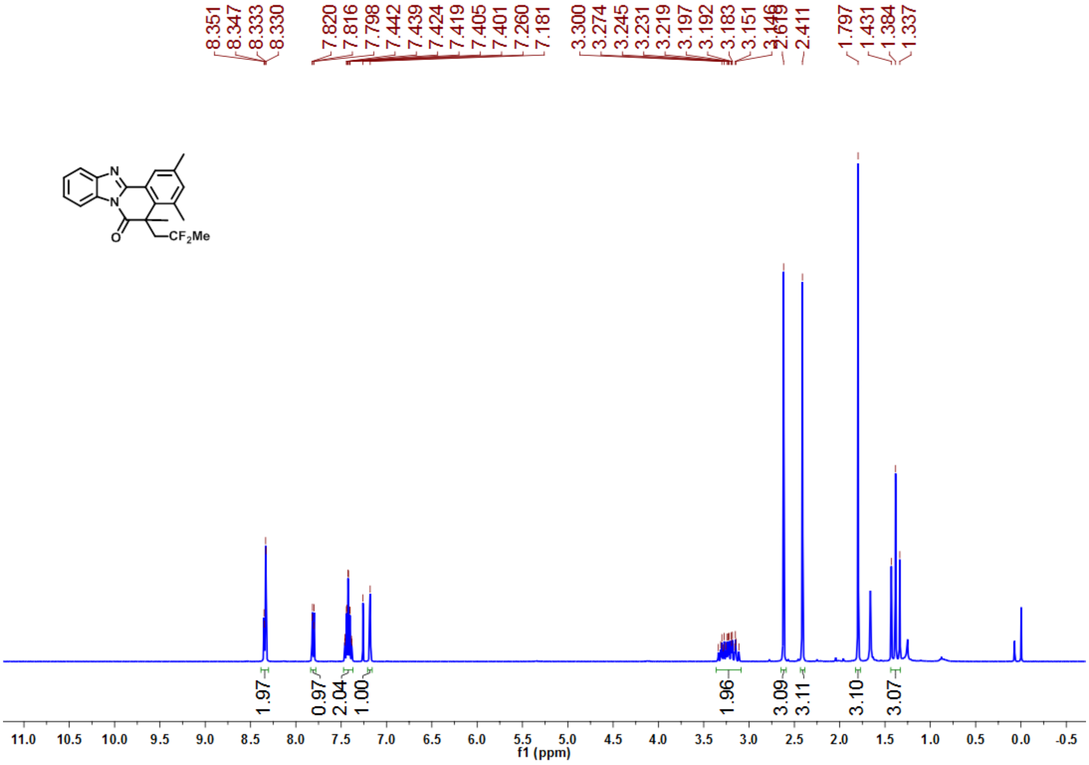

3q-^{13}C{^{1}H} NMR (100 MHz, $CDCl_3$)

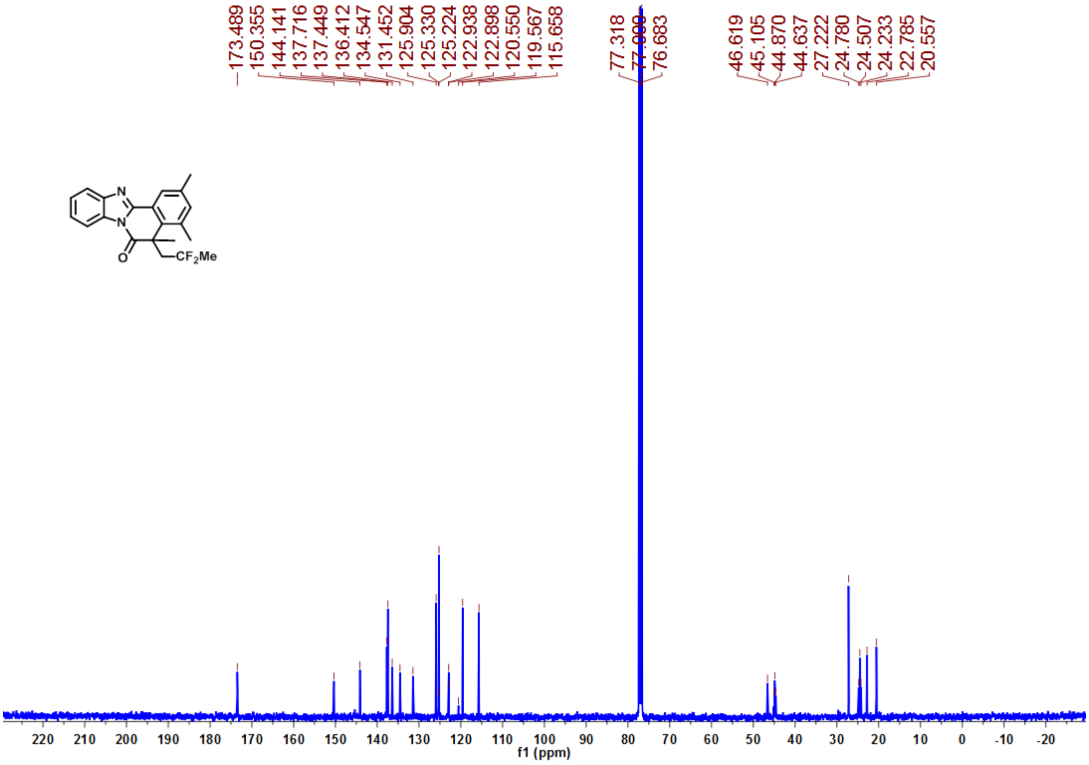

3q-^{19}F NMR (471 MHz, $CDCl_3$)

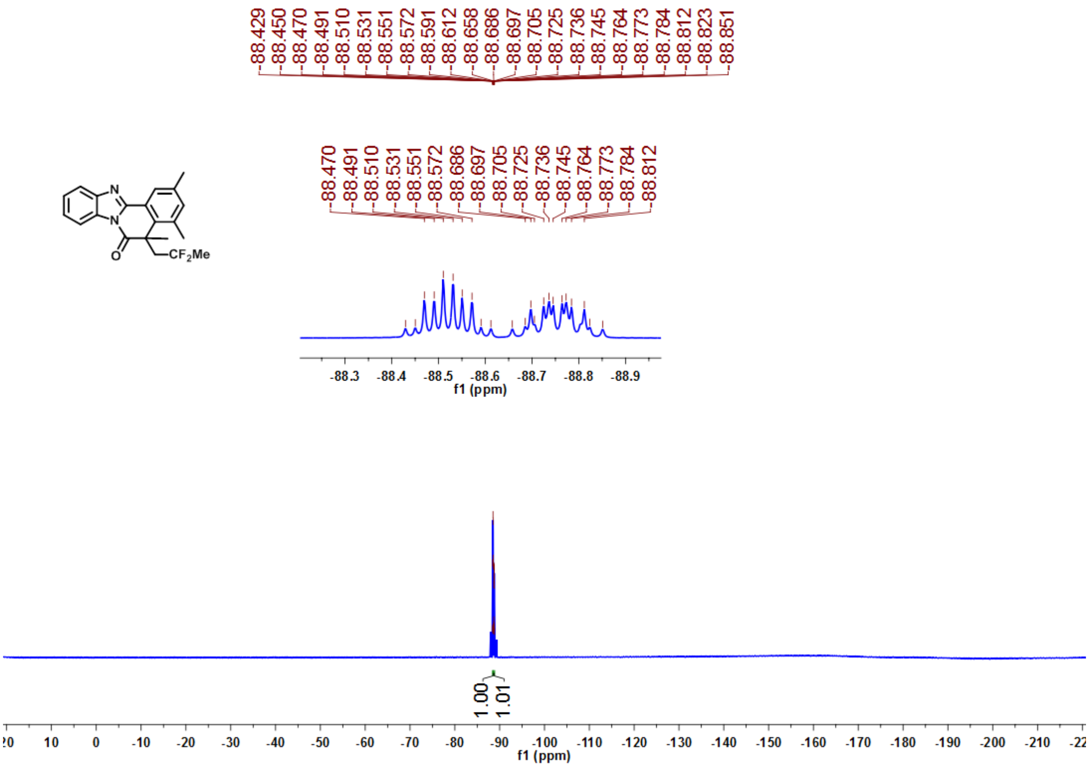

3r-^{1}H NMR (500 MHz, $CDCl_3$)

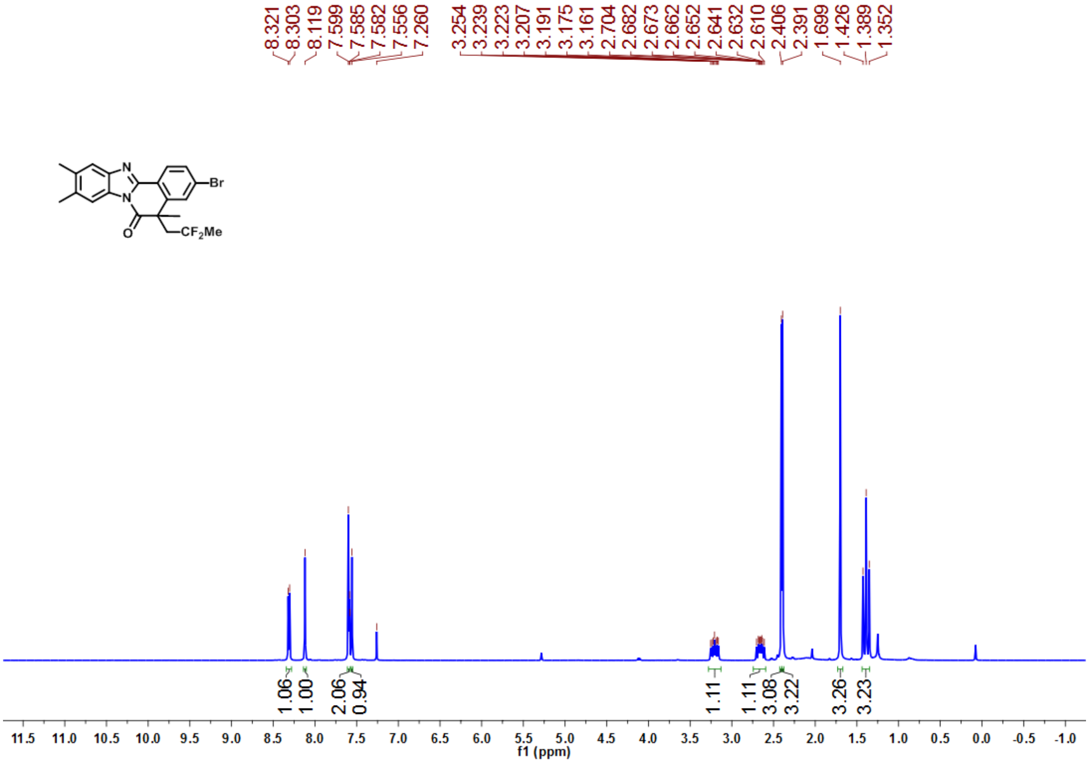

3r-$^{13}C\{^{1}H\}$ NMR (125 MHz, $CDCl_3$)

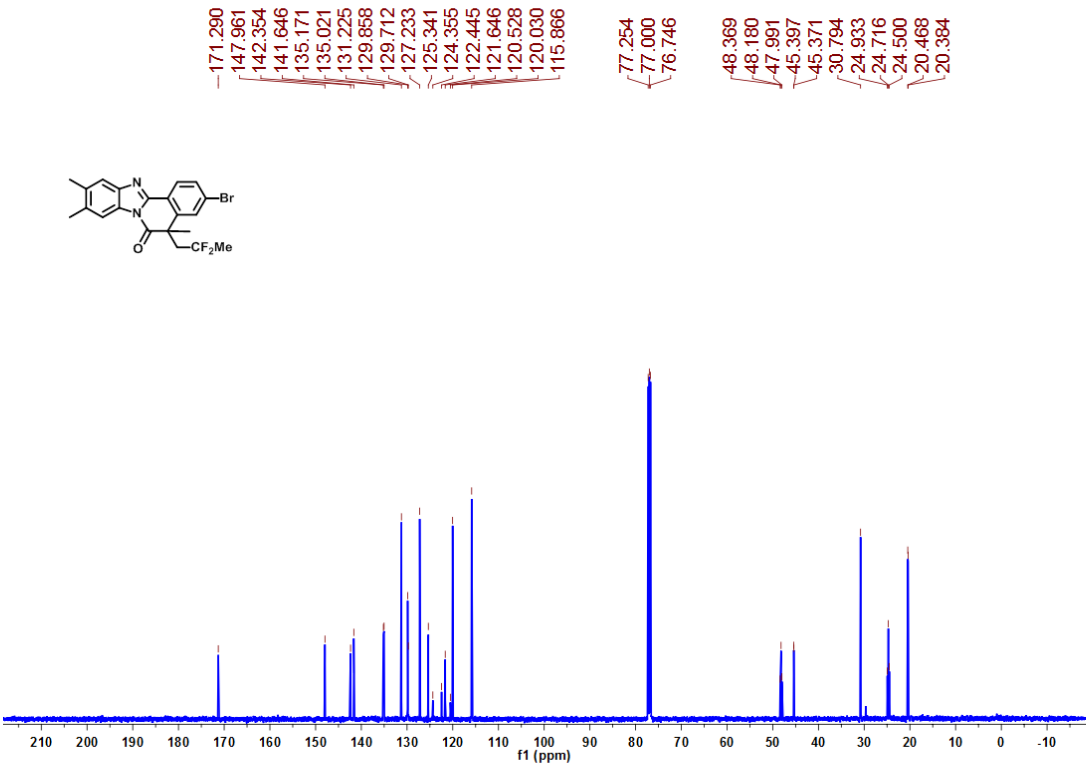

3r-^{19}F NMR (471 MHz, $CDCl_3$)

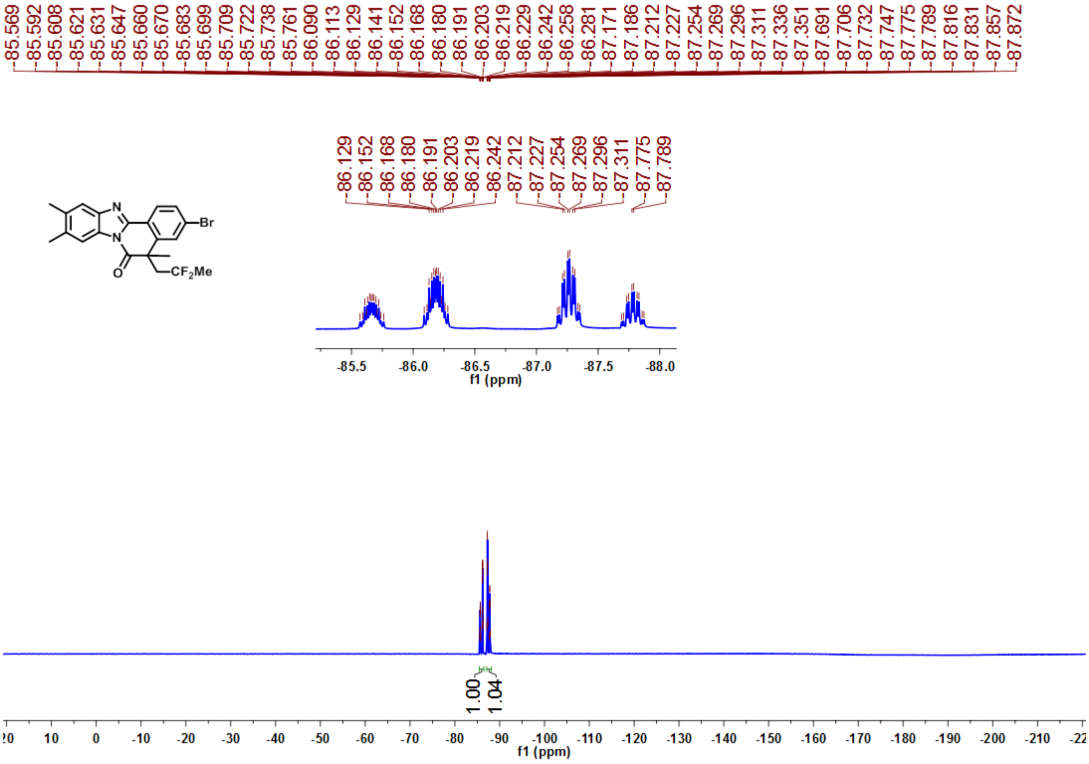

3s-^{1}H NMR (400 MHz, $CDCl_3$)

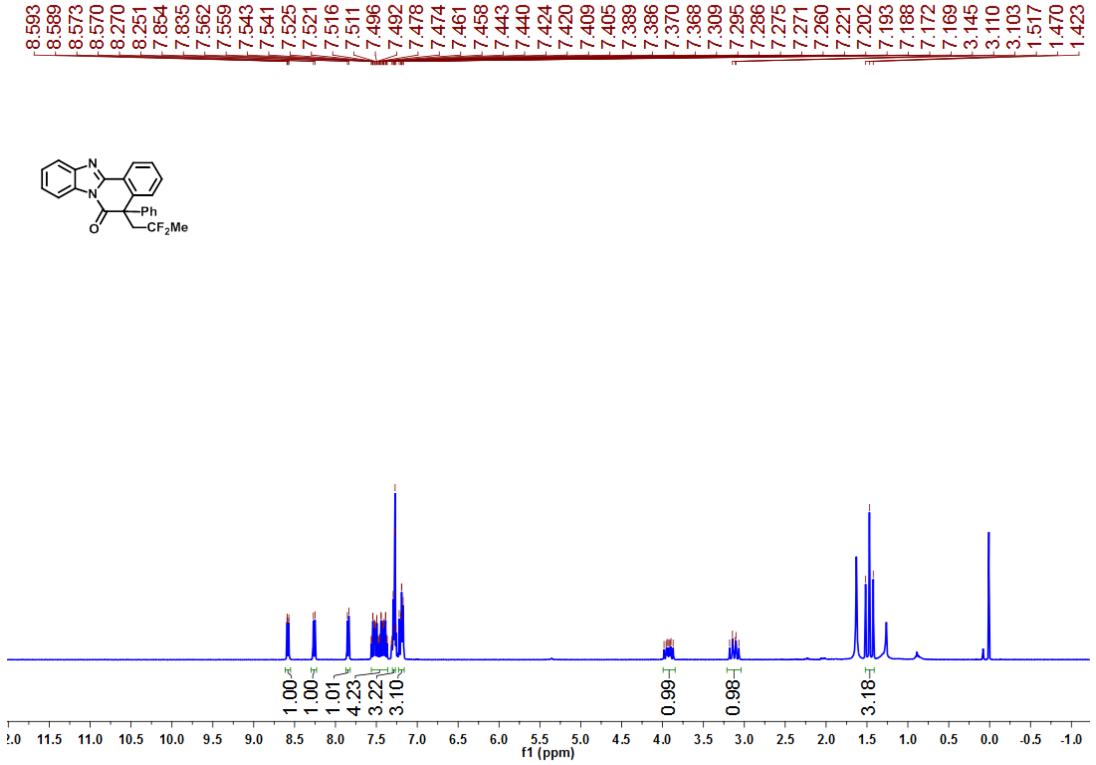

3s-^{13}C{^{1}H} NMR (125 MHz, $CDCl_3$)

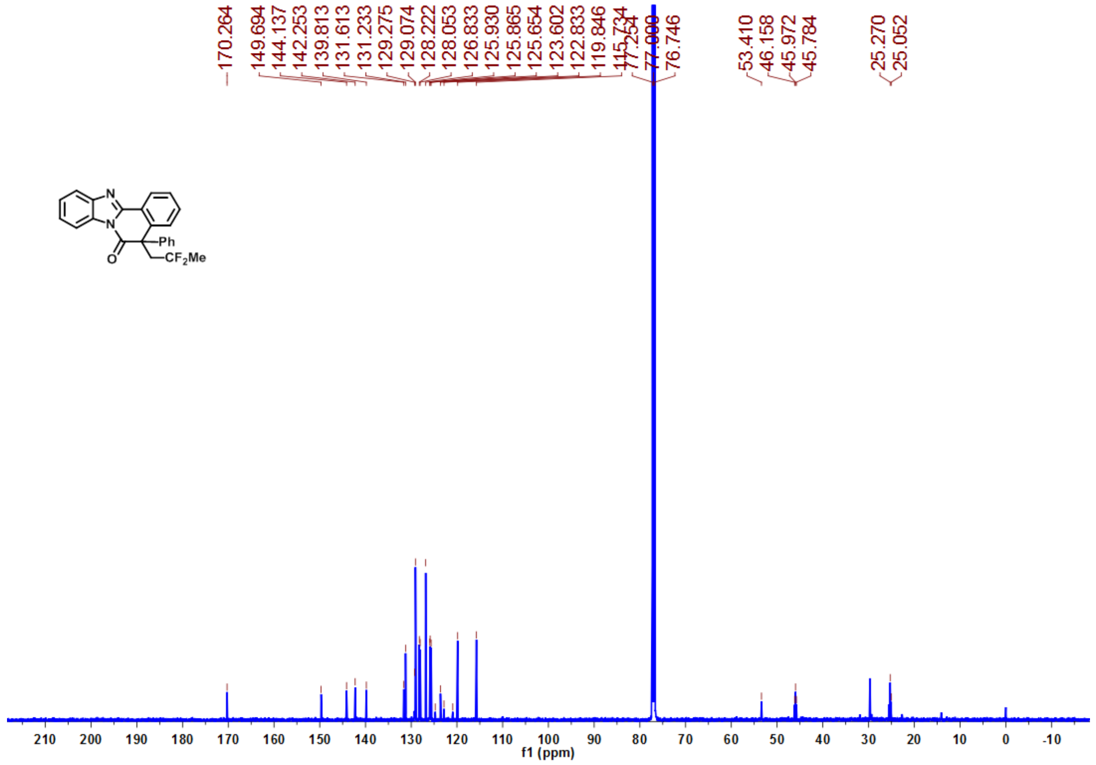

3s-^{19}F NMR (471 MHz, $CDCl_3$)

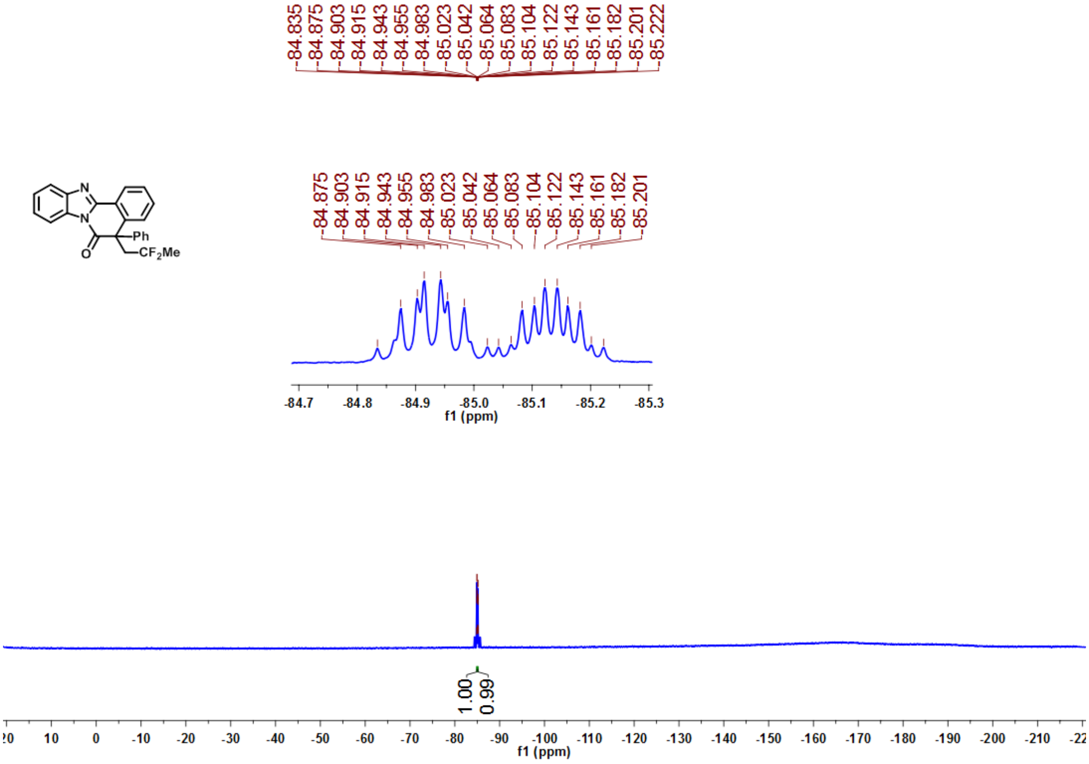

3t-^{1}H NMR (500 MHz, $CDCl_3$)

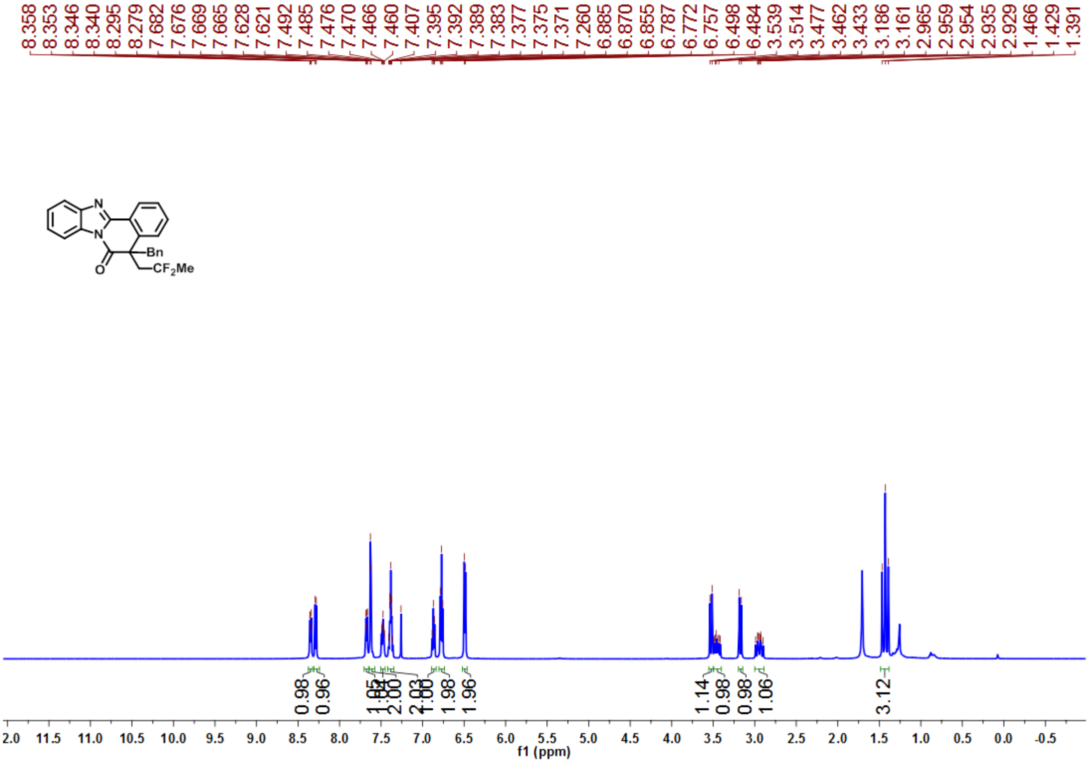

3t-$^{13}C\{^1H\}$ NMR (100 MHz, $CDCl_3$)

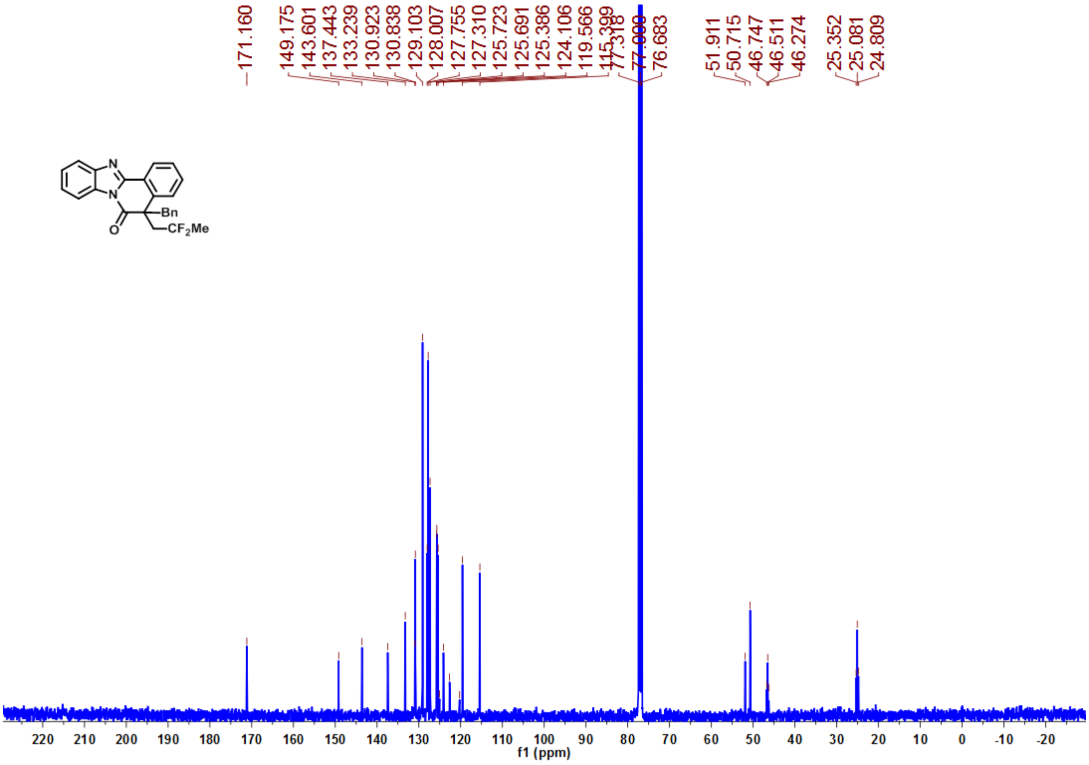

3t-^{19}F NMR (471 MHz, $CDCl_3$)

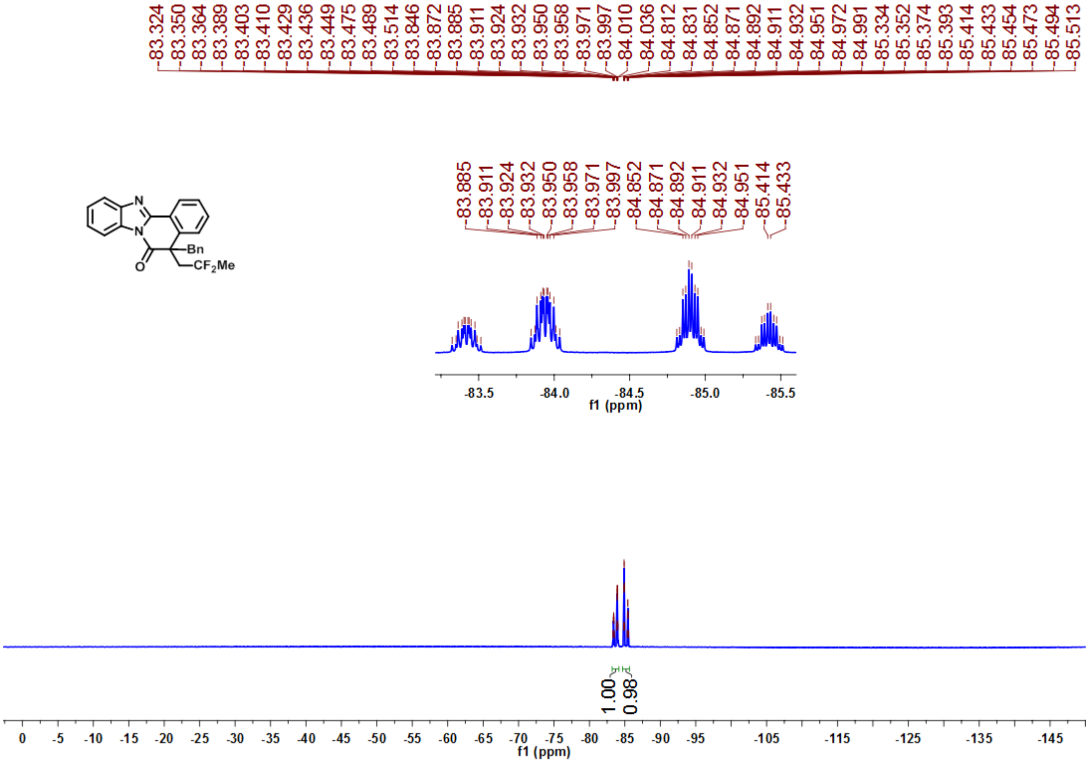

3u-^{1}H NMR (500 MHz, $CDCl_3$)

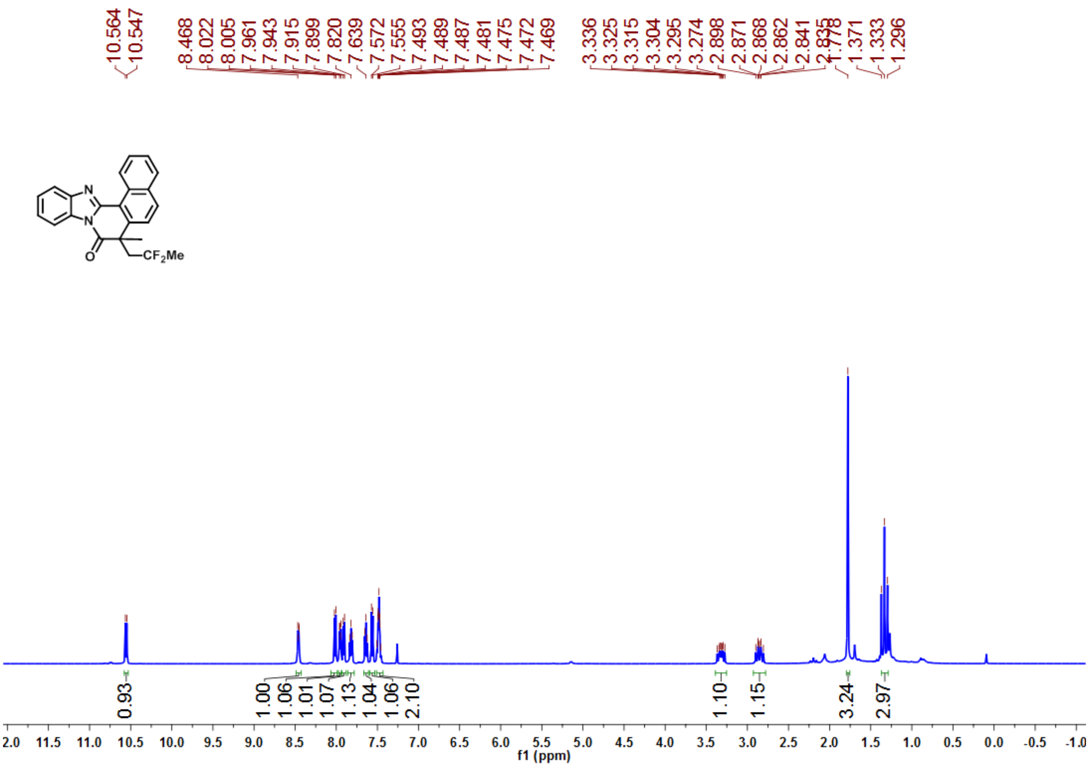

3u-^{13}C{^{1}H} NMR (100 MHz, $CDCl_3$)

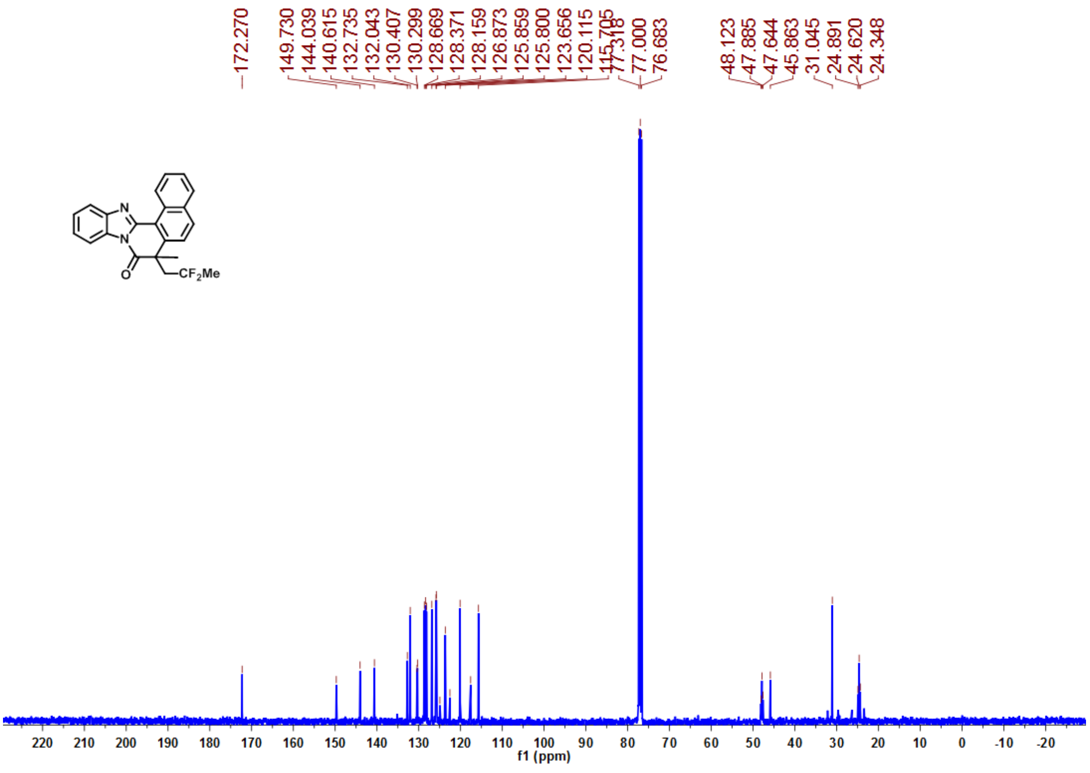

3u-^{19}F NMR (471 MHz, $CDCl_3$)

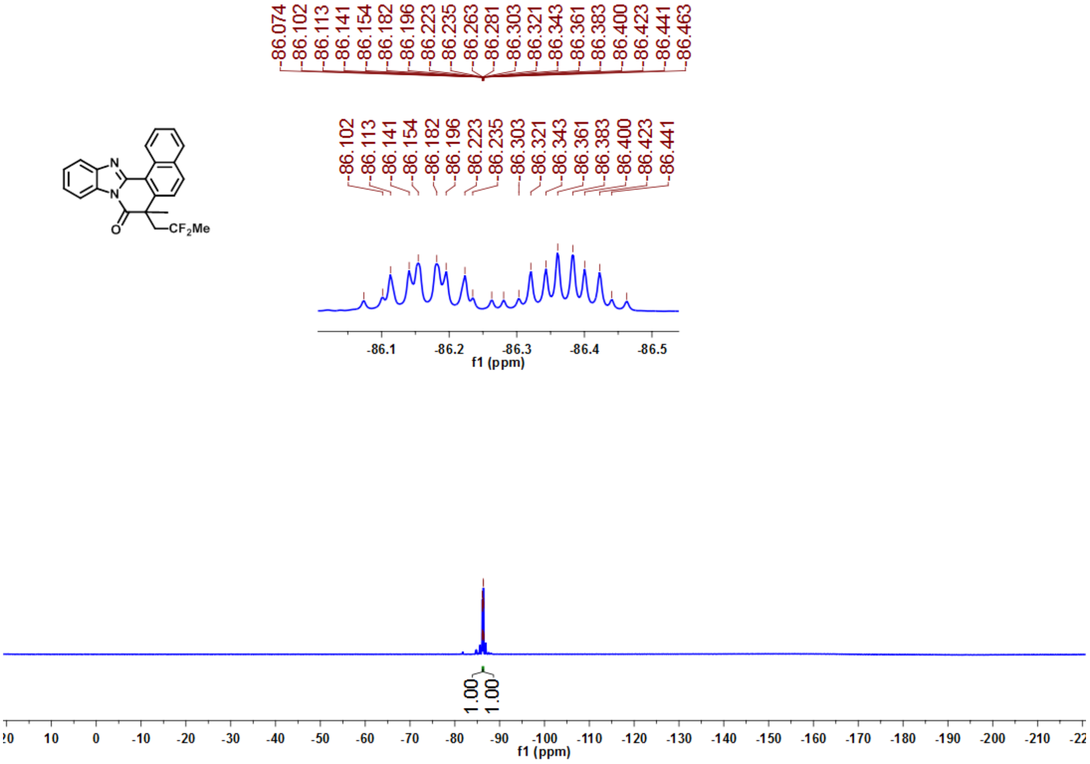

3v-^{1}H NMR (400 MHz, $CDCl_3$)

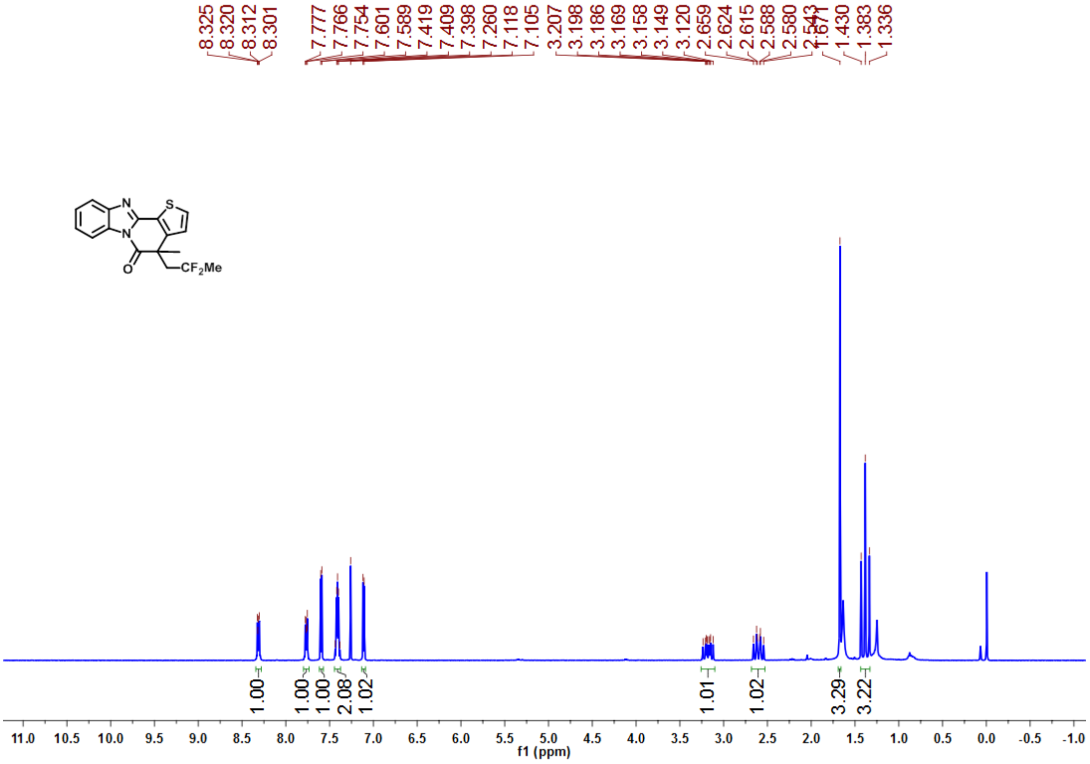

3v-$^{13}C\{^{1}H\}$ NMR (125 MHz, $CDCl_3$)

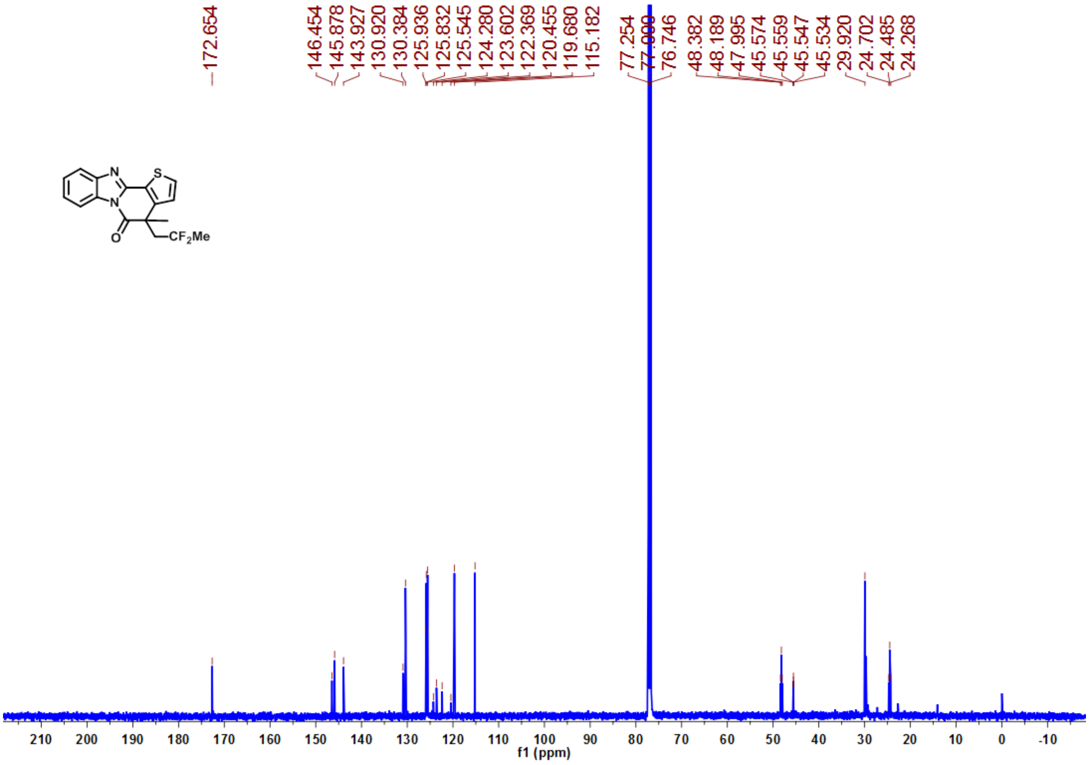

3v-^{19}F NMR (471 MHz, $CDCl_3$)

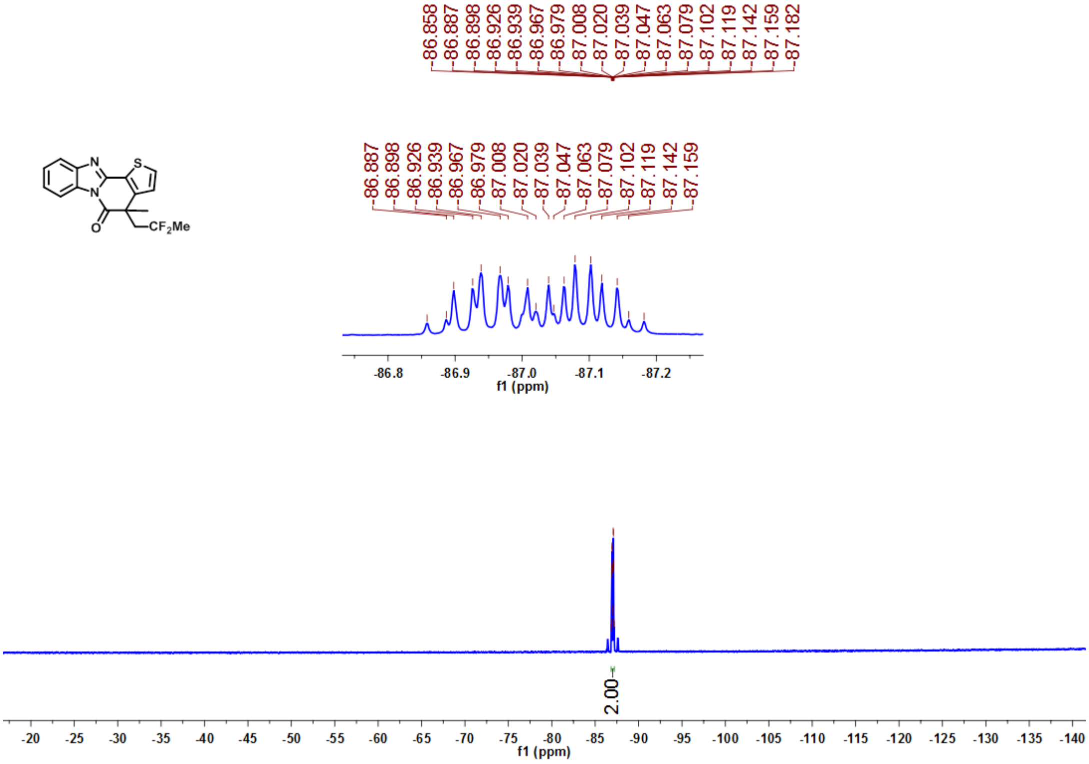

5a-^{1}H NMR (500 MHz, $CDCl_3$)

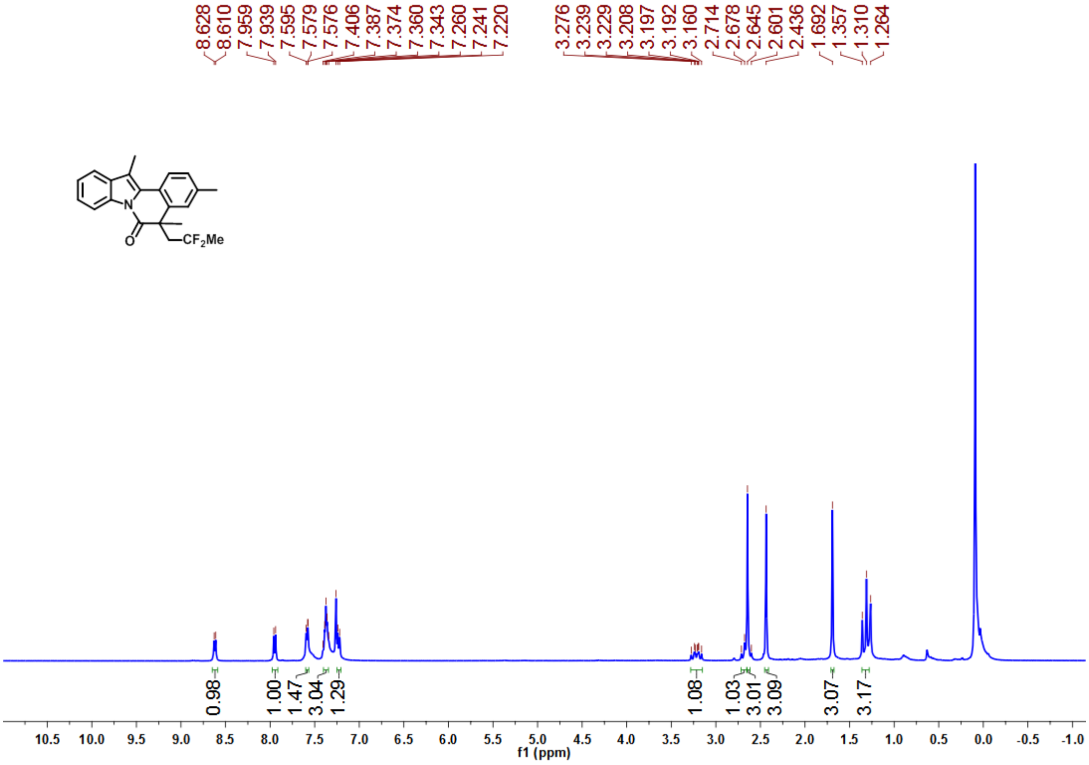

5a-$^{13}C\{^{1}H\}$ NMR (125 MHz, $CDCl_3$)

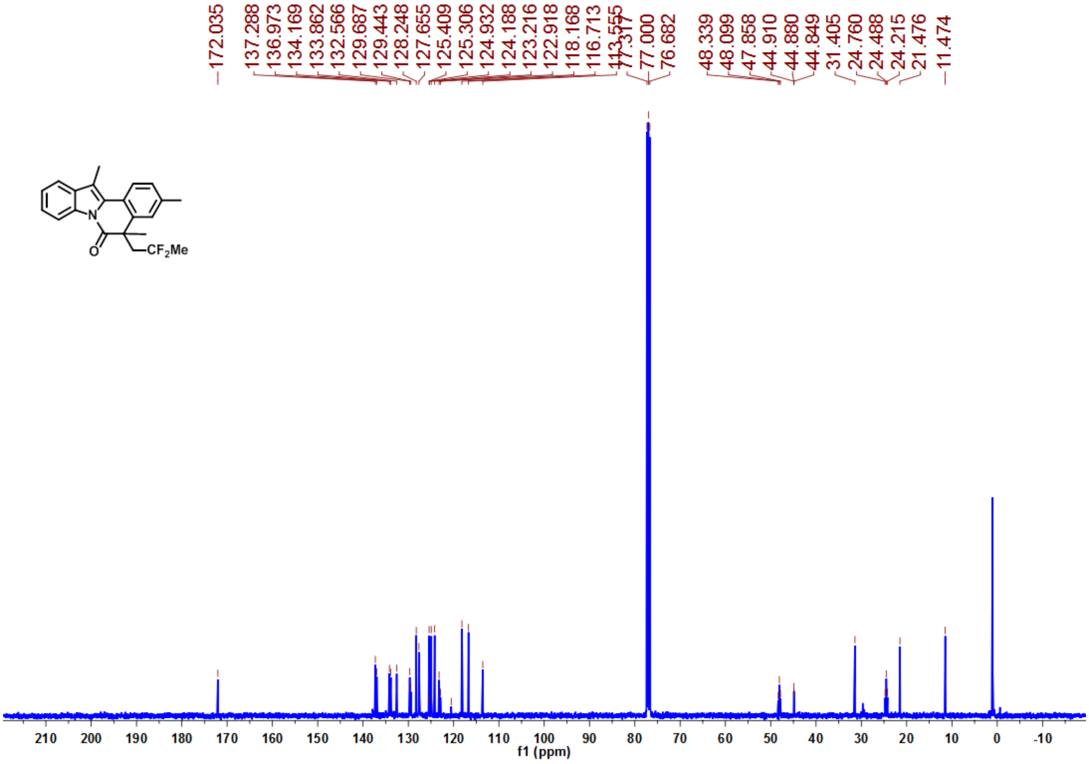

5a-^{19}F NMR (471 MHz, $CDCl_3$)

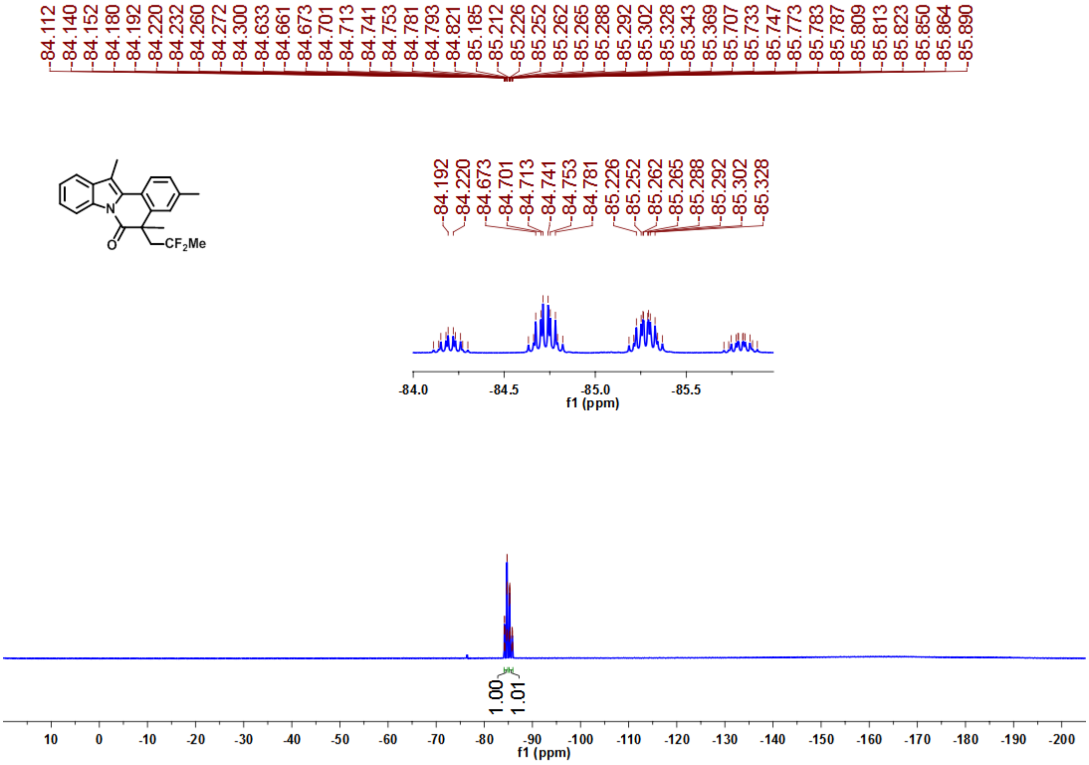

5b-^{1}H NMR (500 MHz, $CDCl_3$)

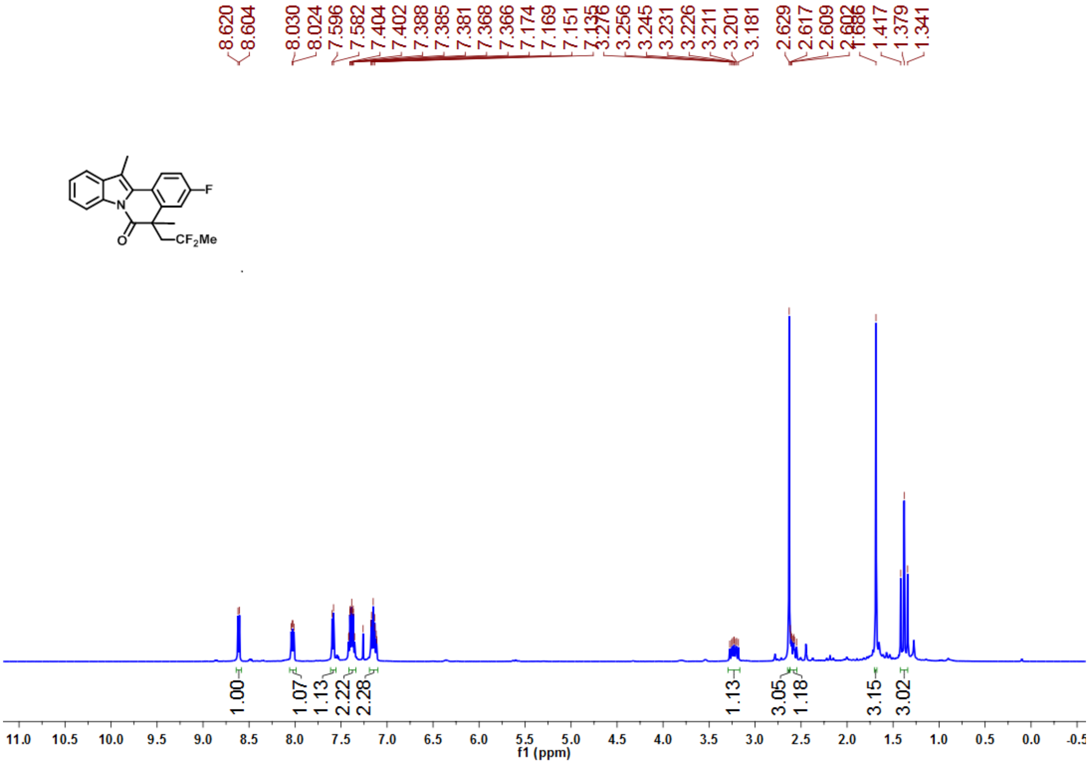

5b-$^{13}C\{^{1}H\}$ NMR (125 MHz, $CDCl_3$)

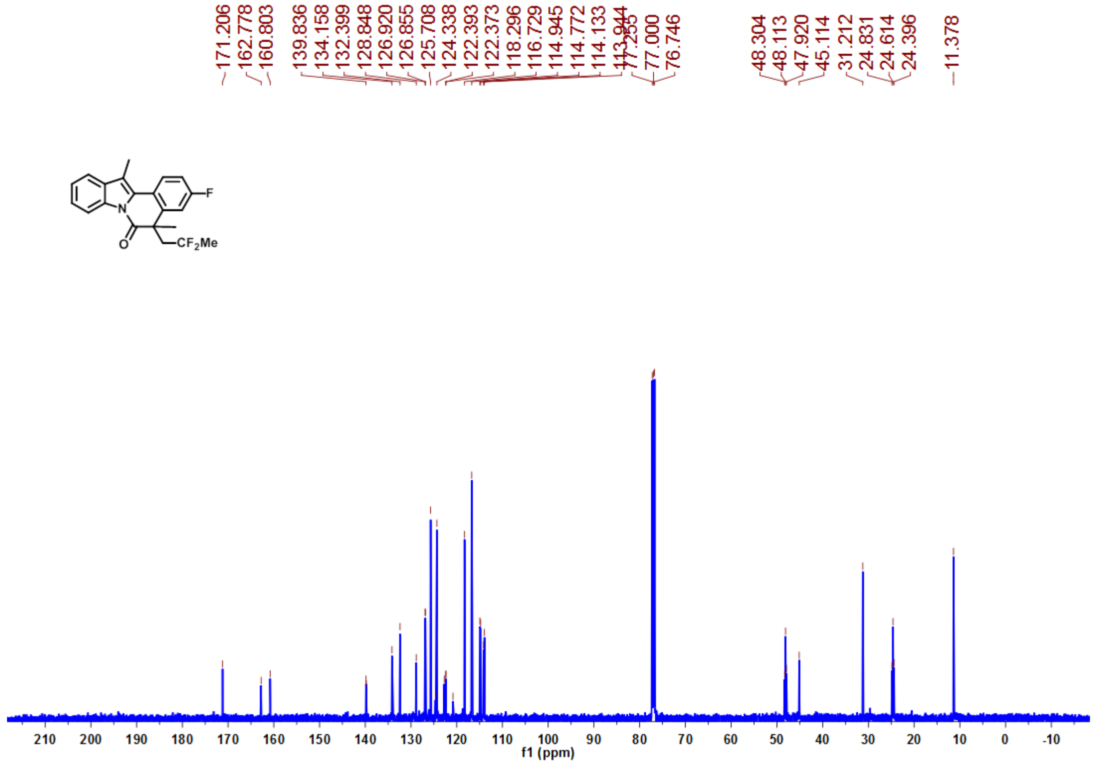

5b-^{19}F NMR (471 MHz, $CDCl_3$)

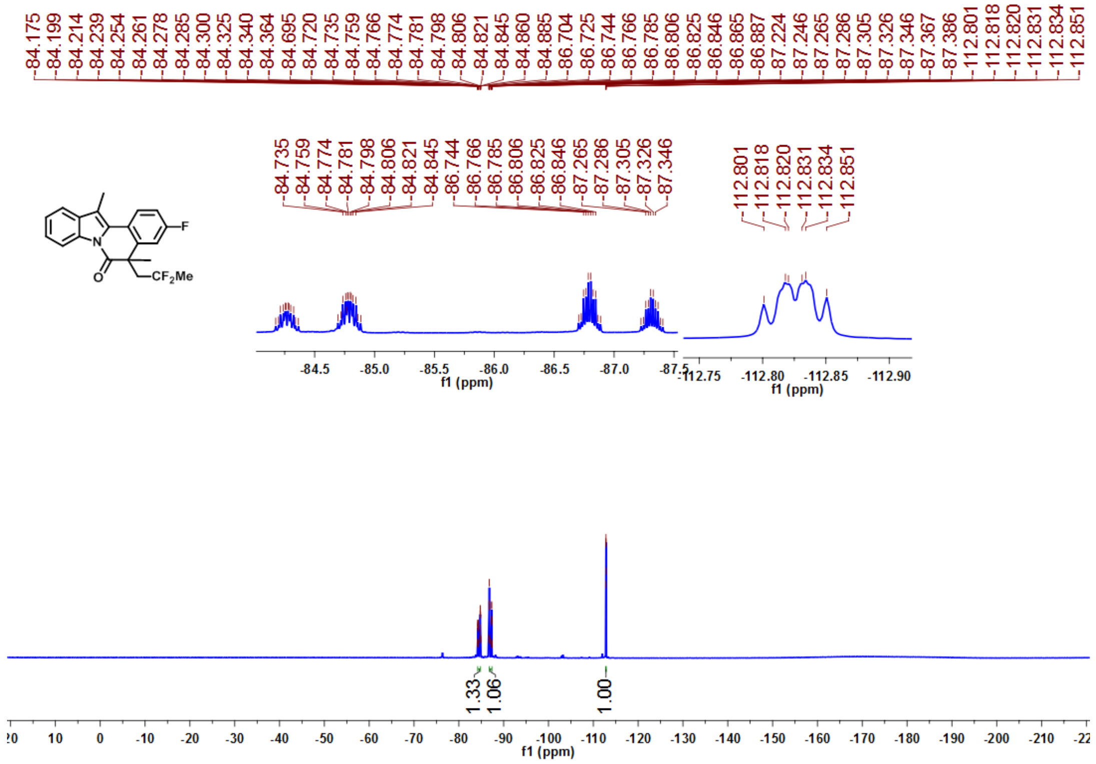

5c-^{1}H NMR (400 MHz, $CDCl_3$)

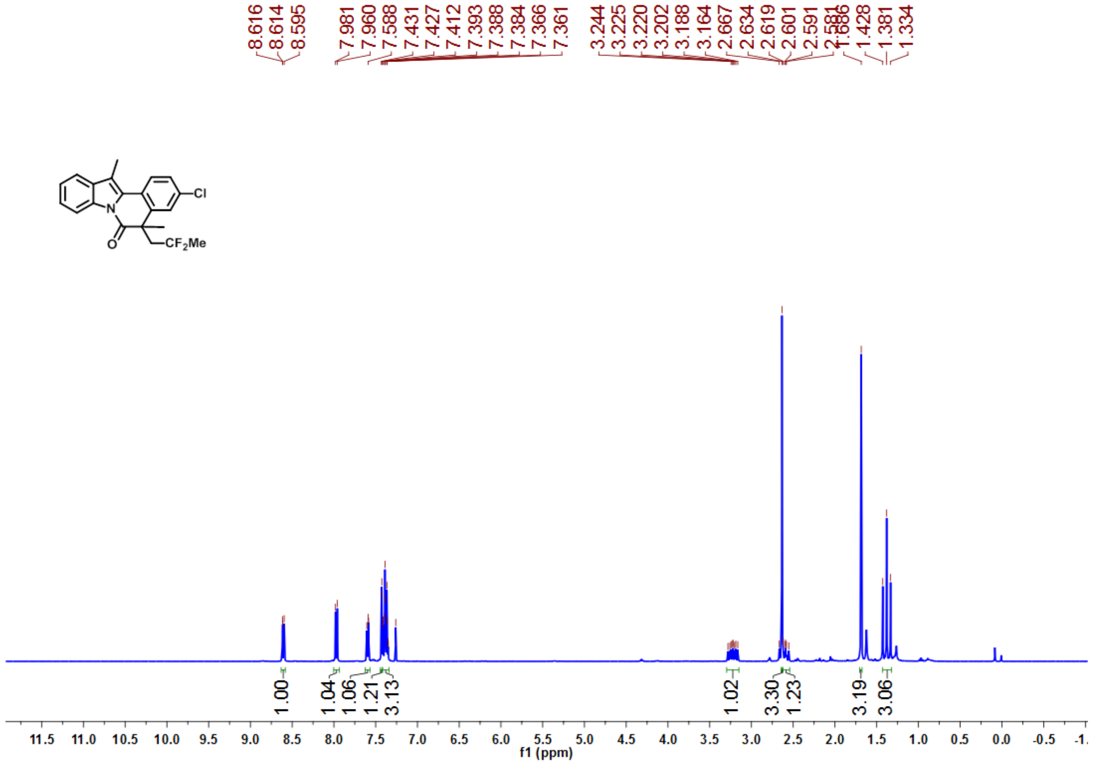

5c-$^{13}C\{^{1}H\}$ NMR (100 MHz, $CDCl_3$)

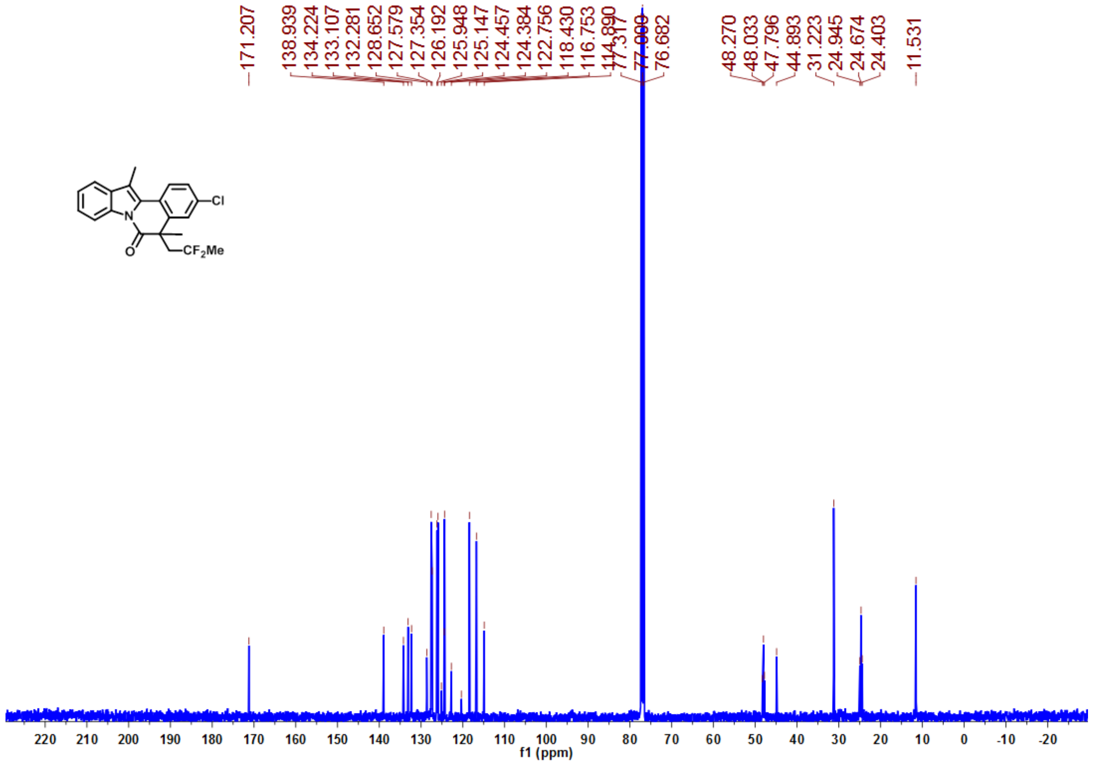

5c-^{19}F NMR (471 MHz, $CDCl_3$)

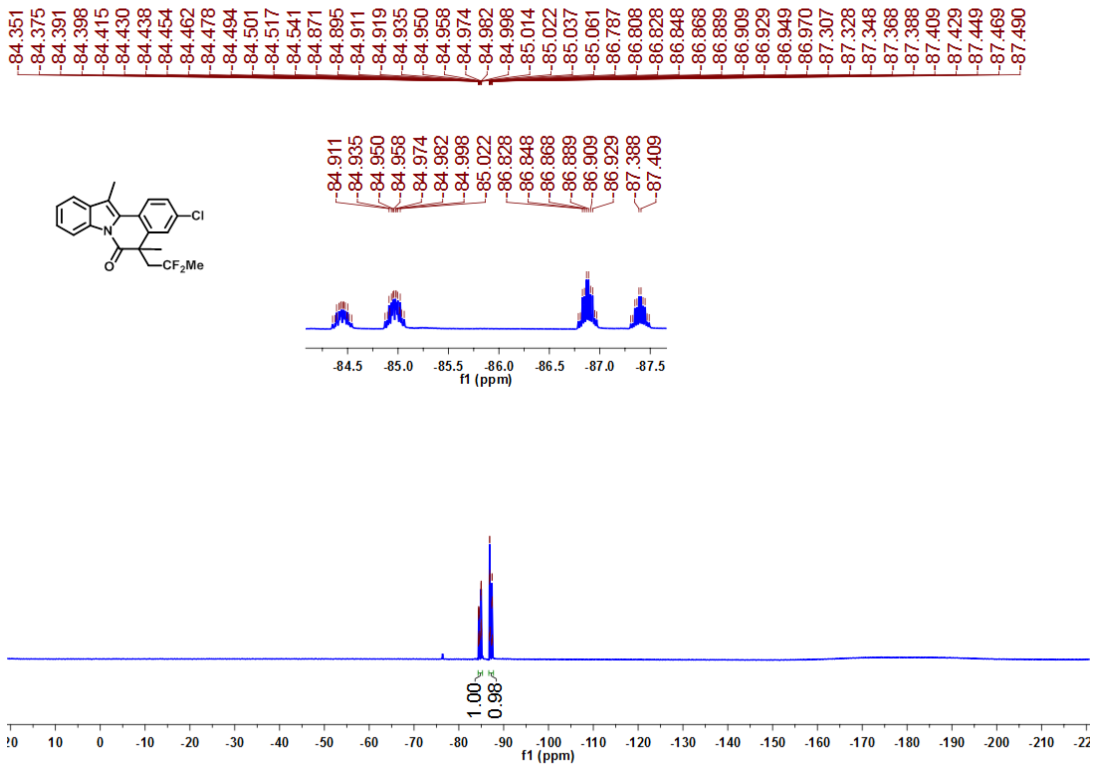

5d-^{1}H NMR (500 MHz, $CDCl_3$)

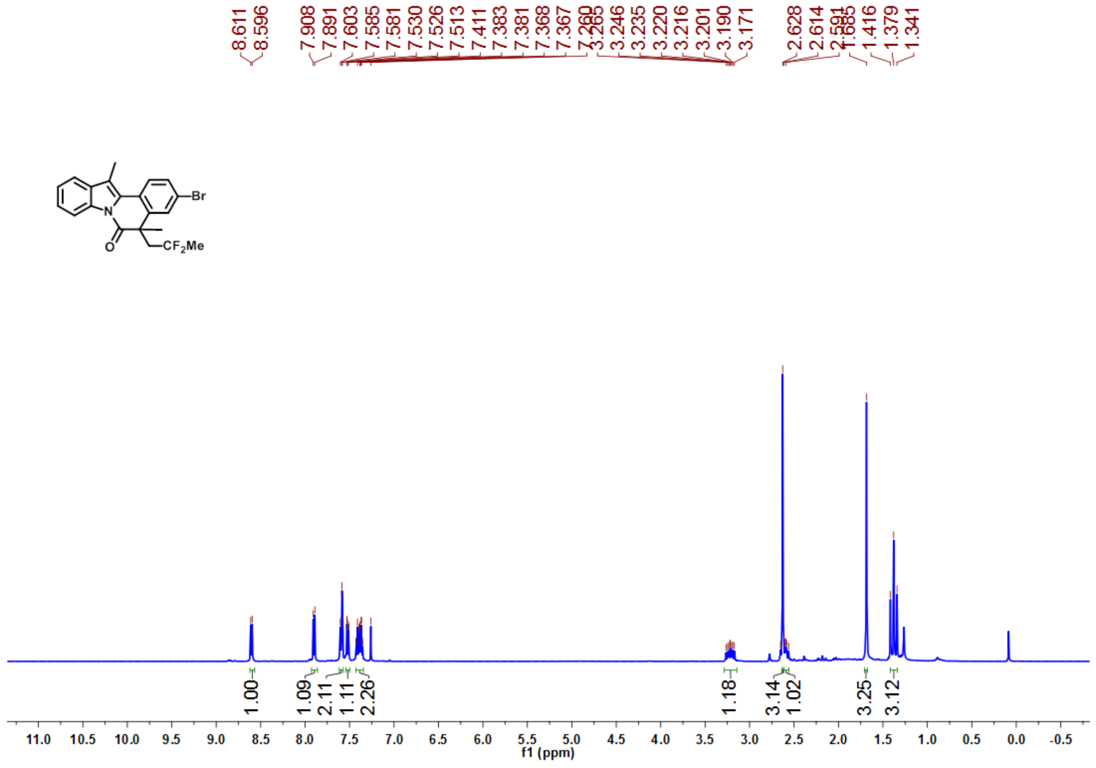

5d-$^{13}C\{^{1}H\}$ NMR (125 MHz, $CDCl_3$)

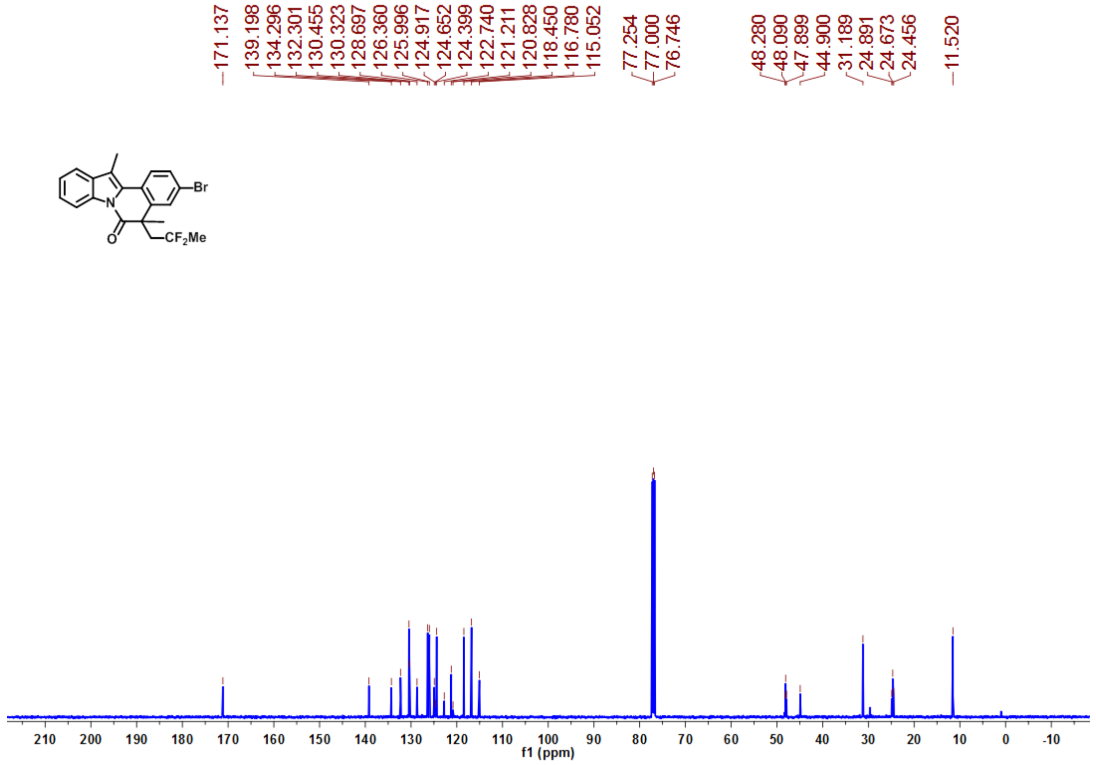

5d-^{19}F NMR (471 MHz, $CDCl_3$)

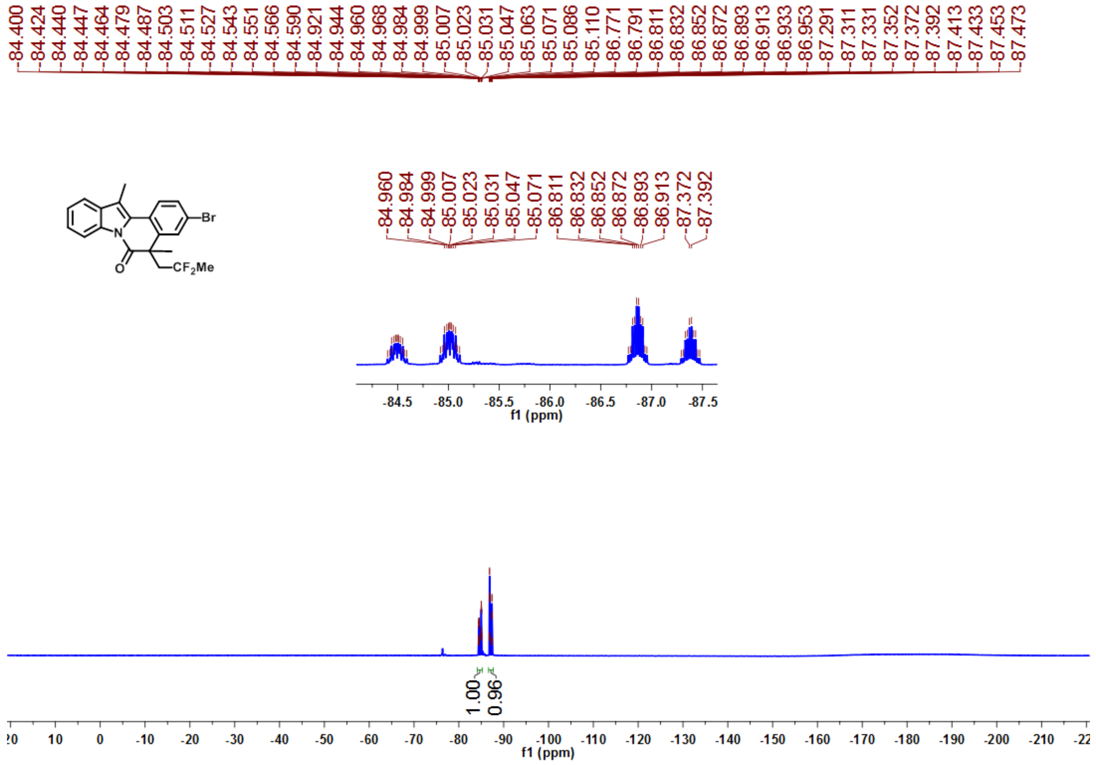

5e-^{1}H NMR (500 MHz, $CDCl_3$)

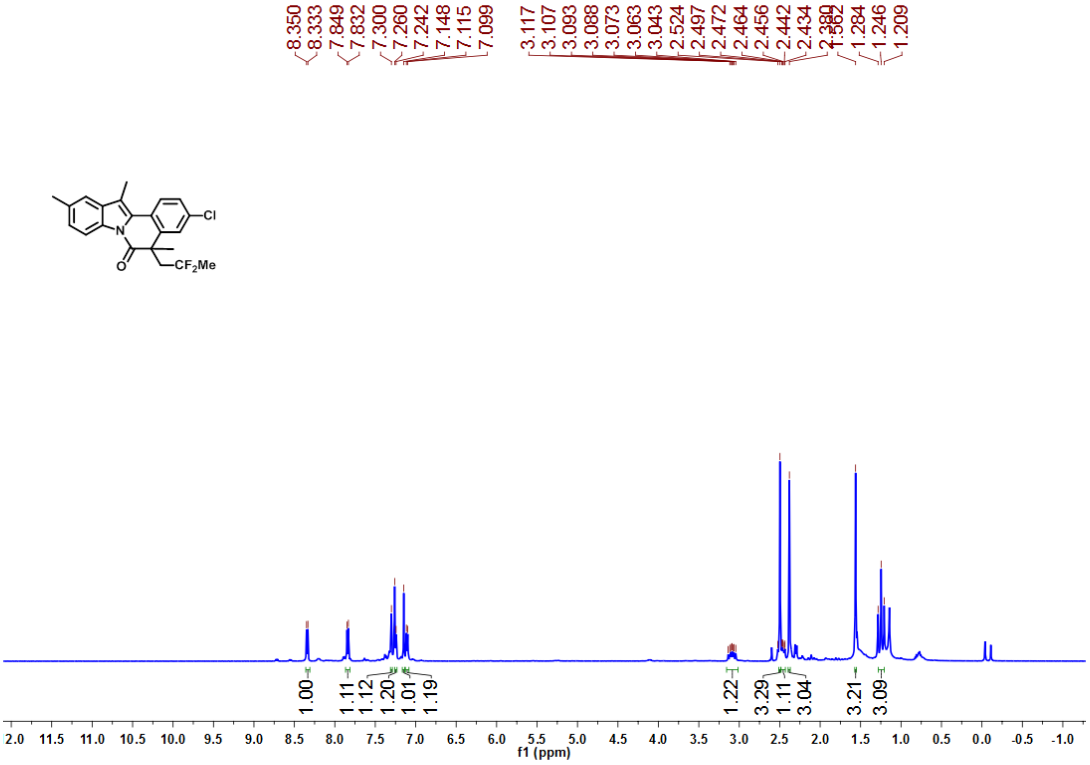

5e-^{13}C{^{1}H} NMR (125 MHz, $CDCl_3$)

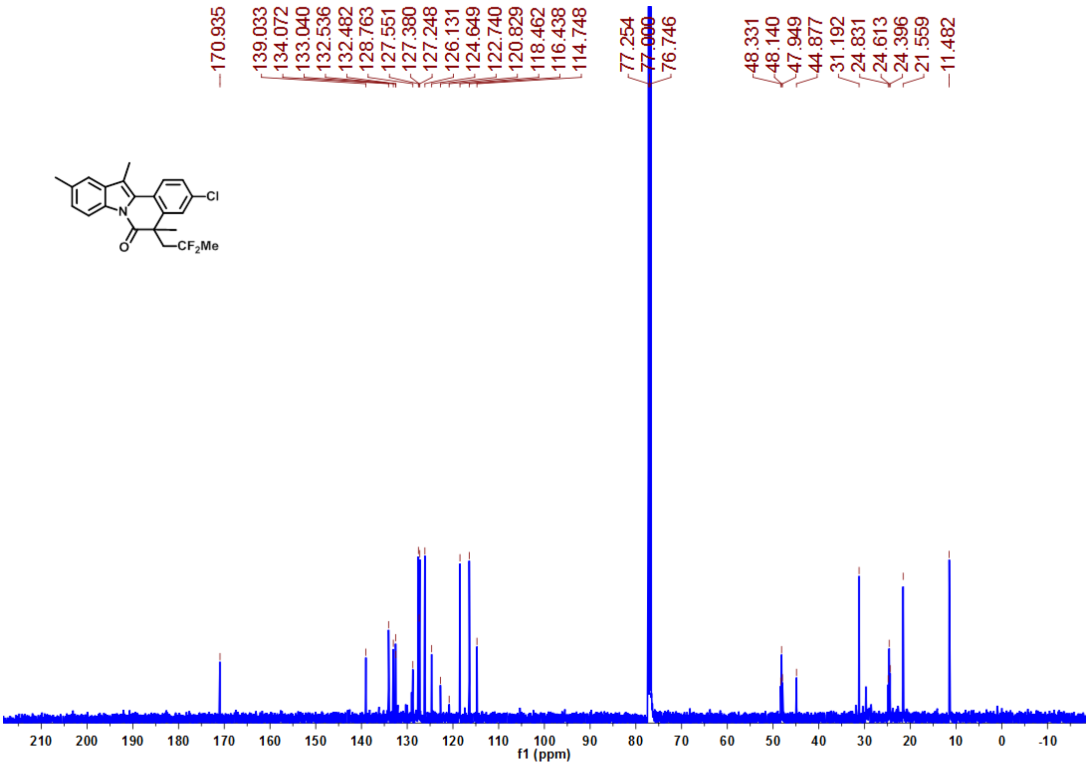

5e-^{19}F NMR (471 MHz, $CDCl_3$)

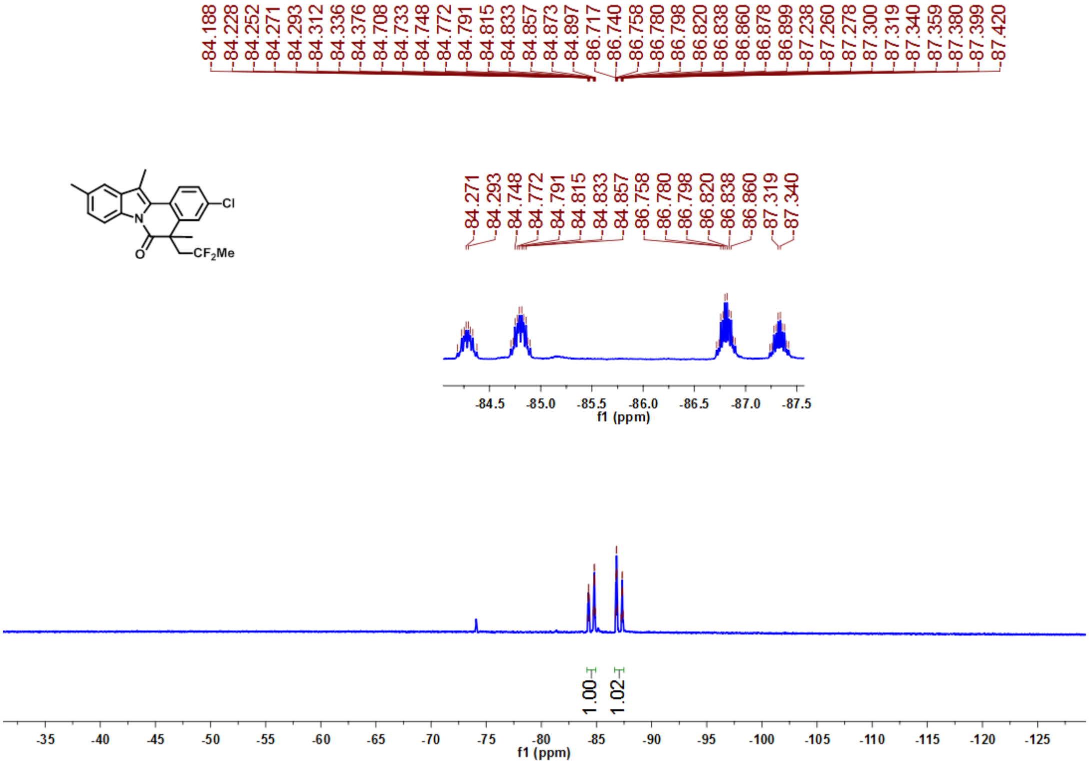

5f-^{1}H NMR (500 MHz, $CDCl_3$)

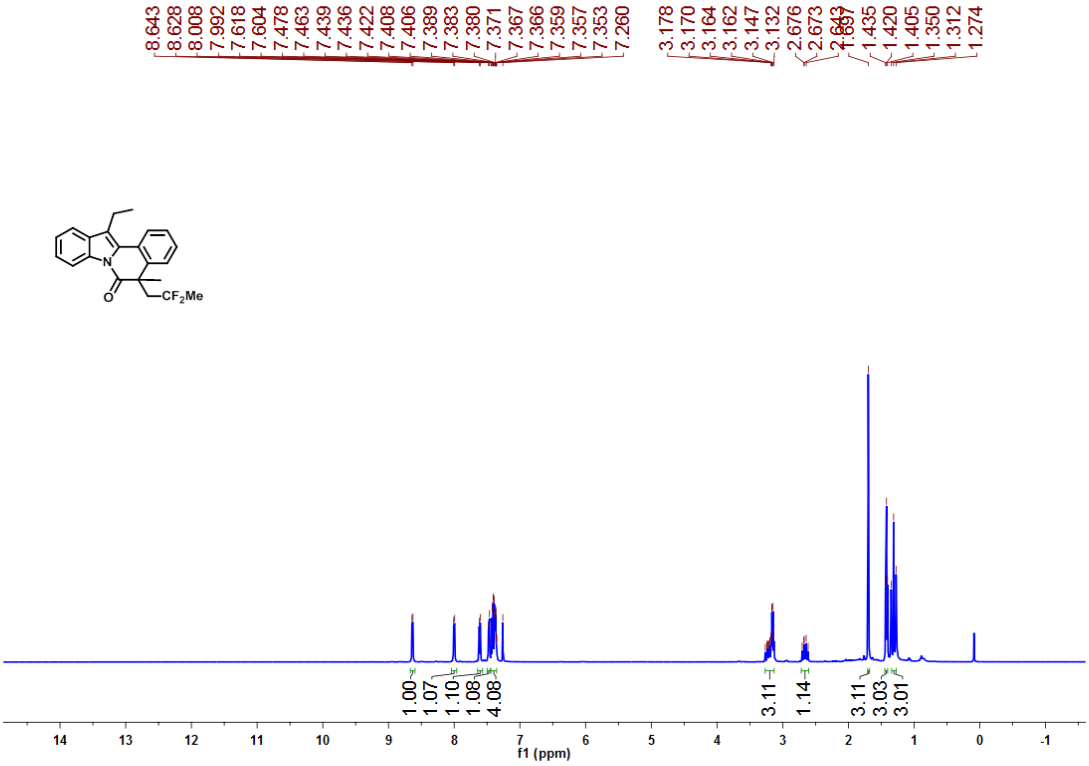

5f-$^{13}C\{^1H\}$ NMR (125 MHz, $CDCl_3$)

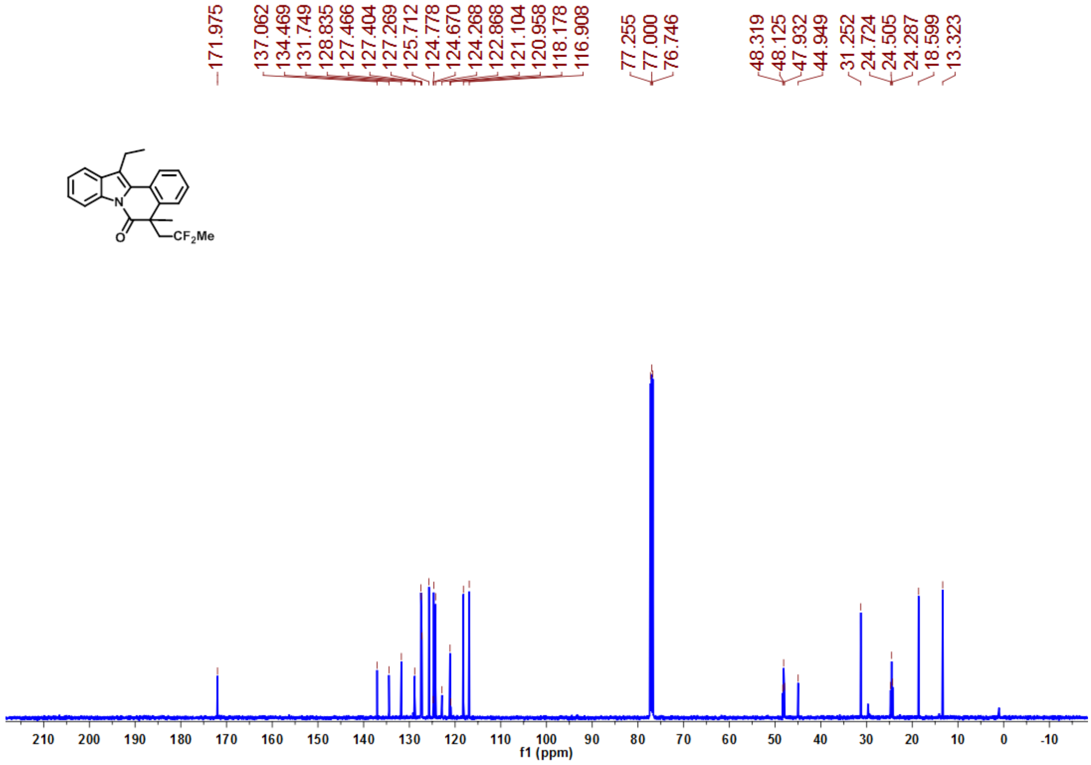

5f-^{19}F NMR (471 MHz, $CDCl_3$)

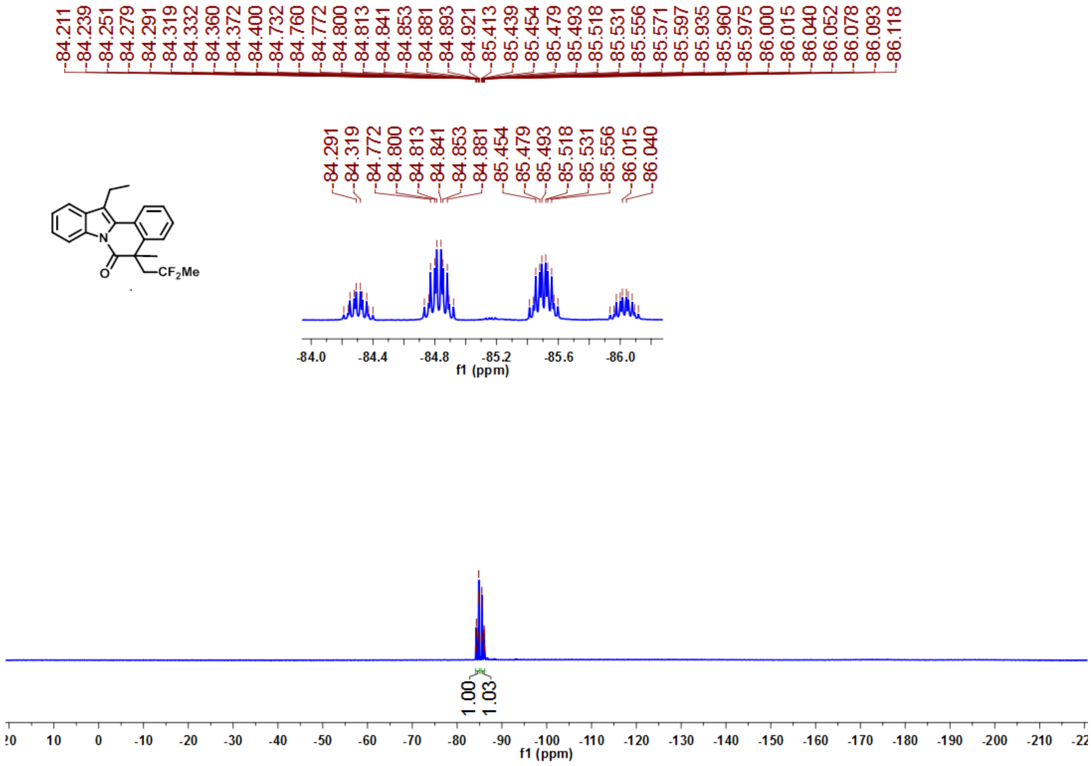

5g-^{1}H NMR (500 MHz, $CDCl_3$)

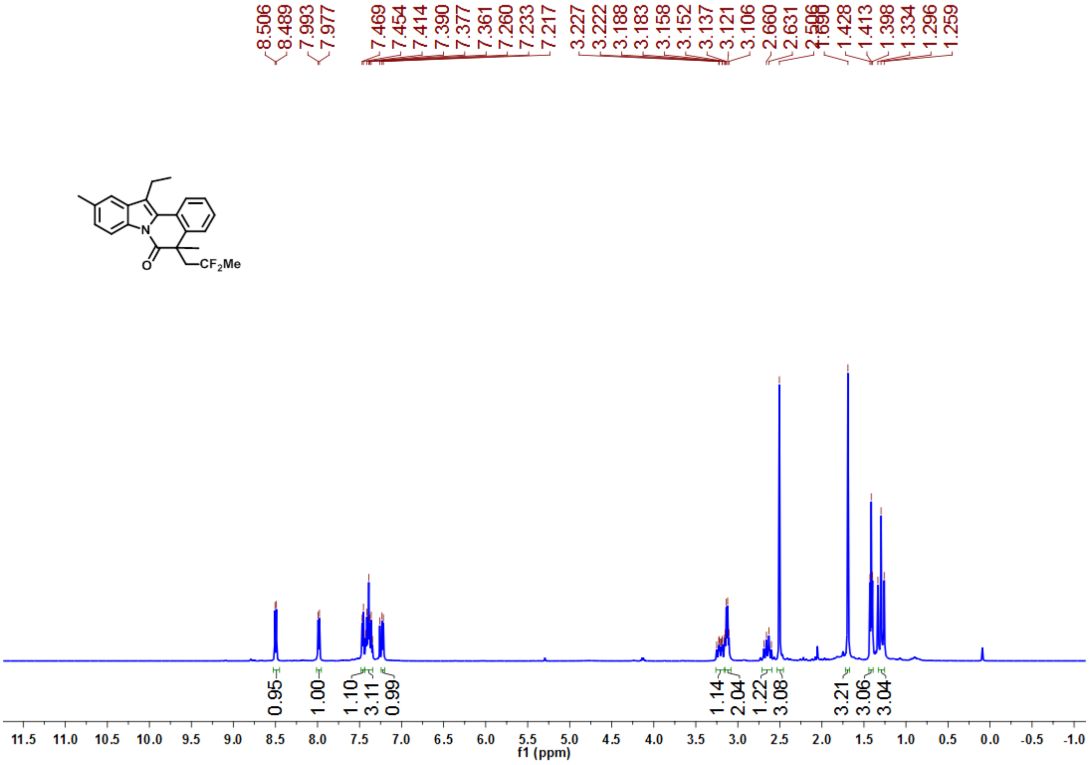

5g-^{13}C{^{1}H} NMR (125 MHz, $CDCl_3$)

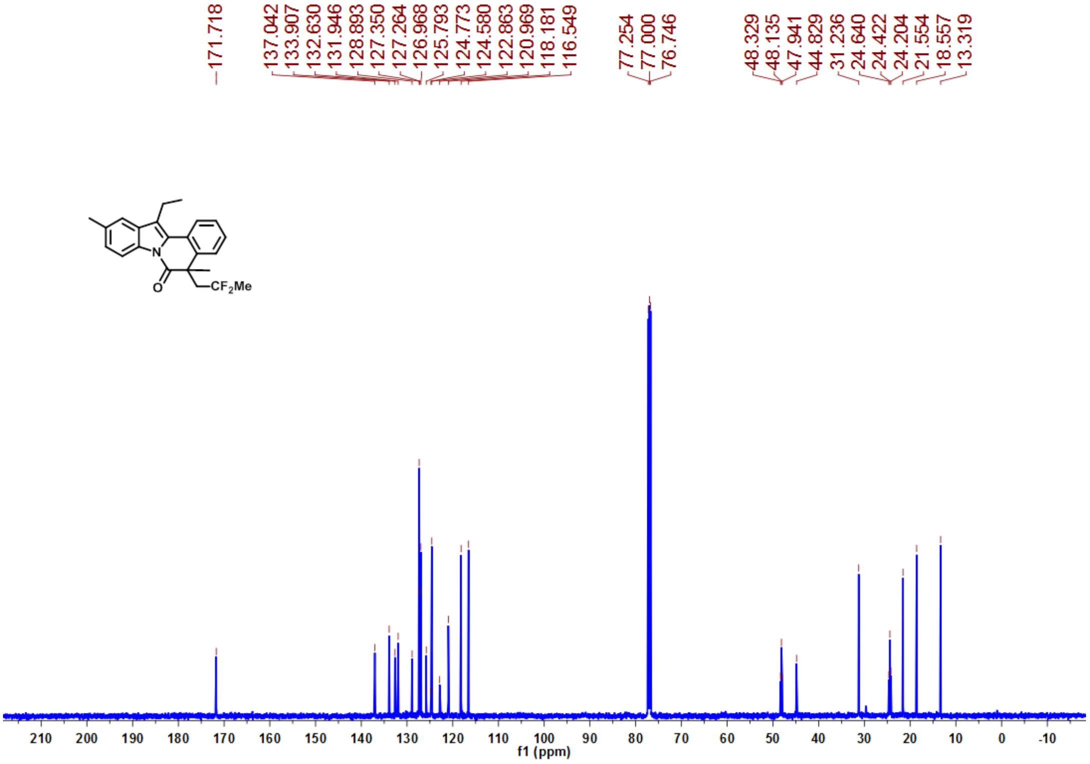

5g-^{19}F NMR (471 MHz, $CDCl_3$)

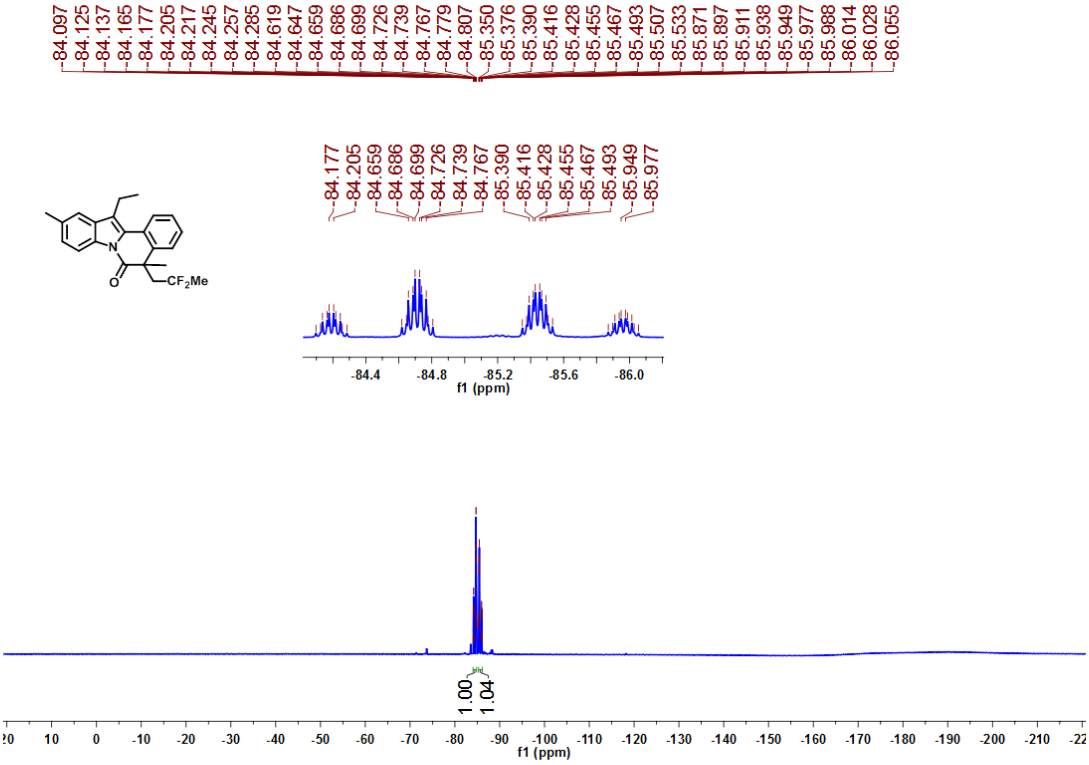

5h-^{1}H NMR (500 MHz, $CDCl_3$)

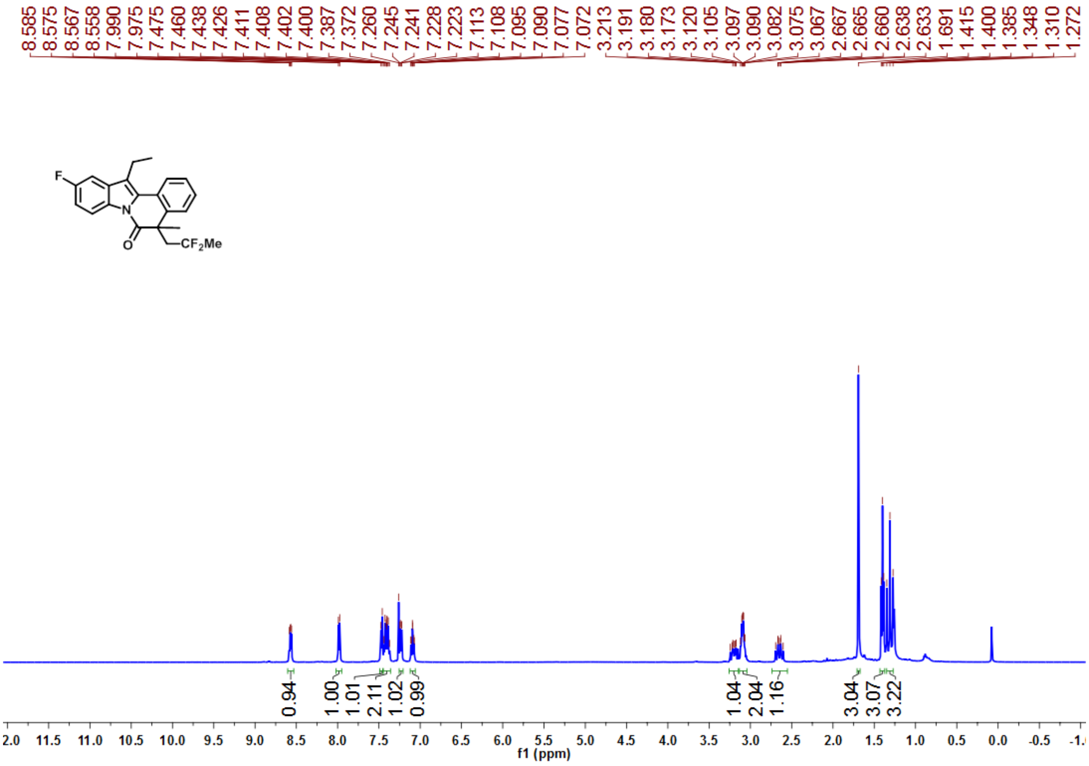

5h-$^{13}C\{^1H\}$ NMR (125 MHz, $CDCl_3$)

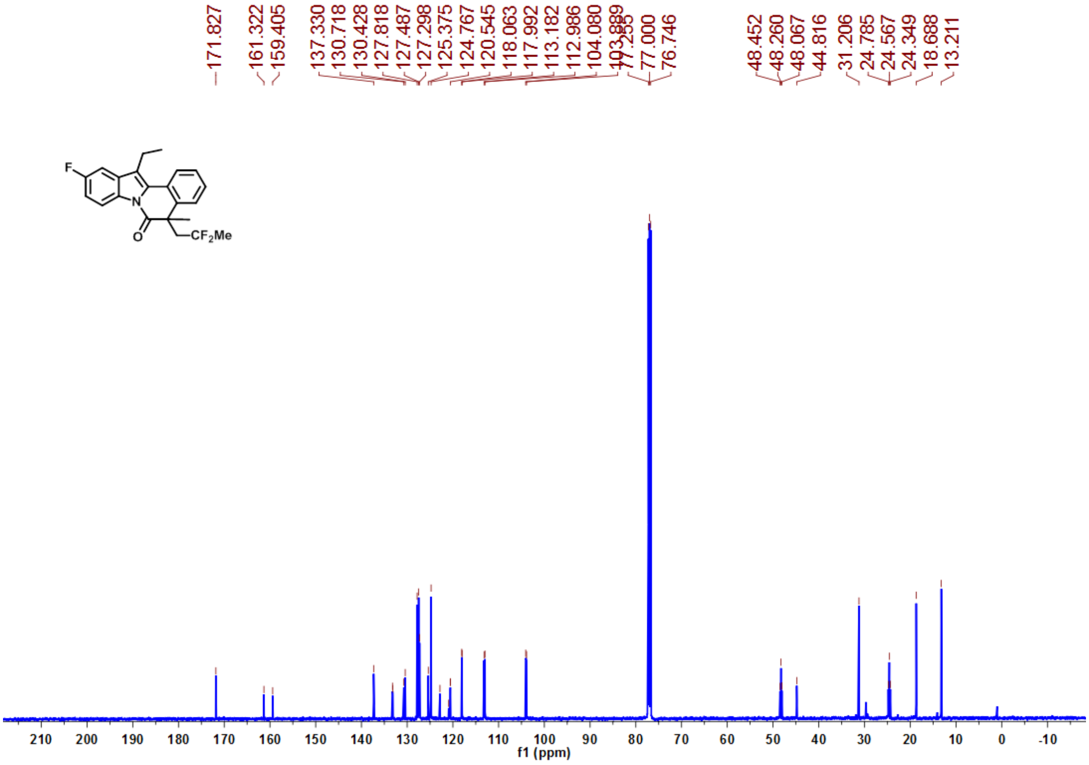

5h-^{19}F NMR (471 MHz, $CDCl_3$)

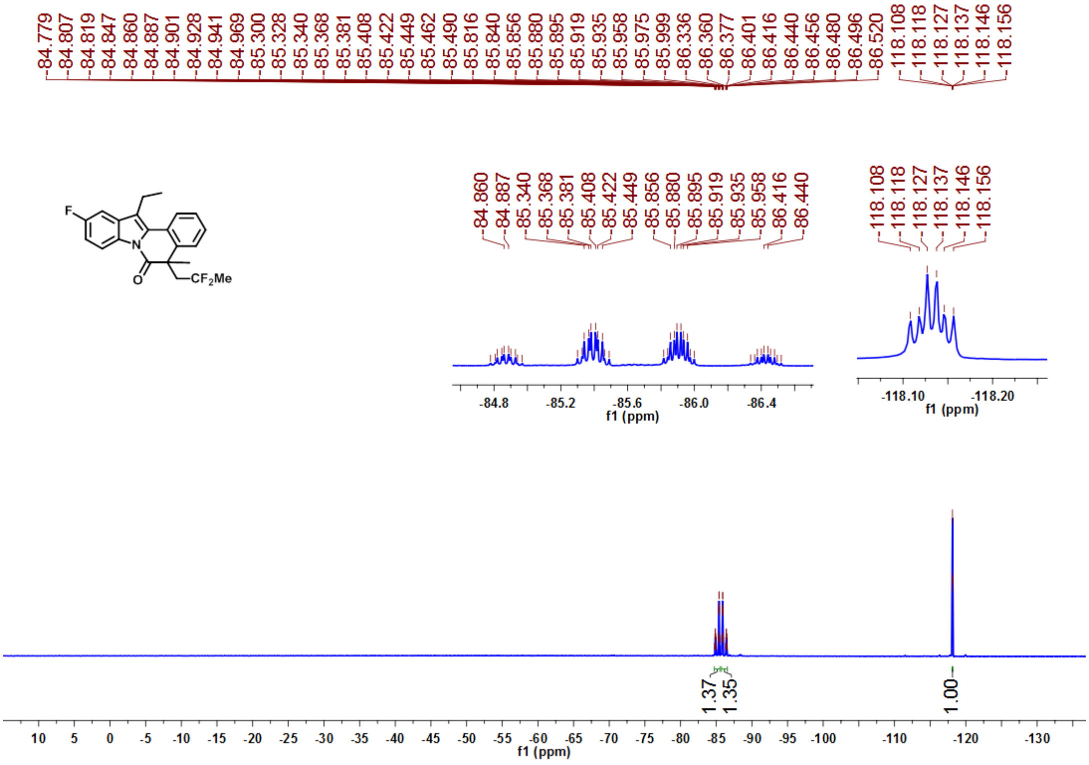

5i-^{1}H NMR (500 MHz, $CDCl_3$)

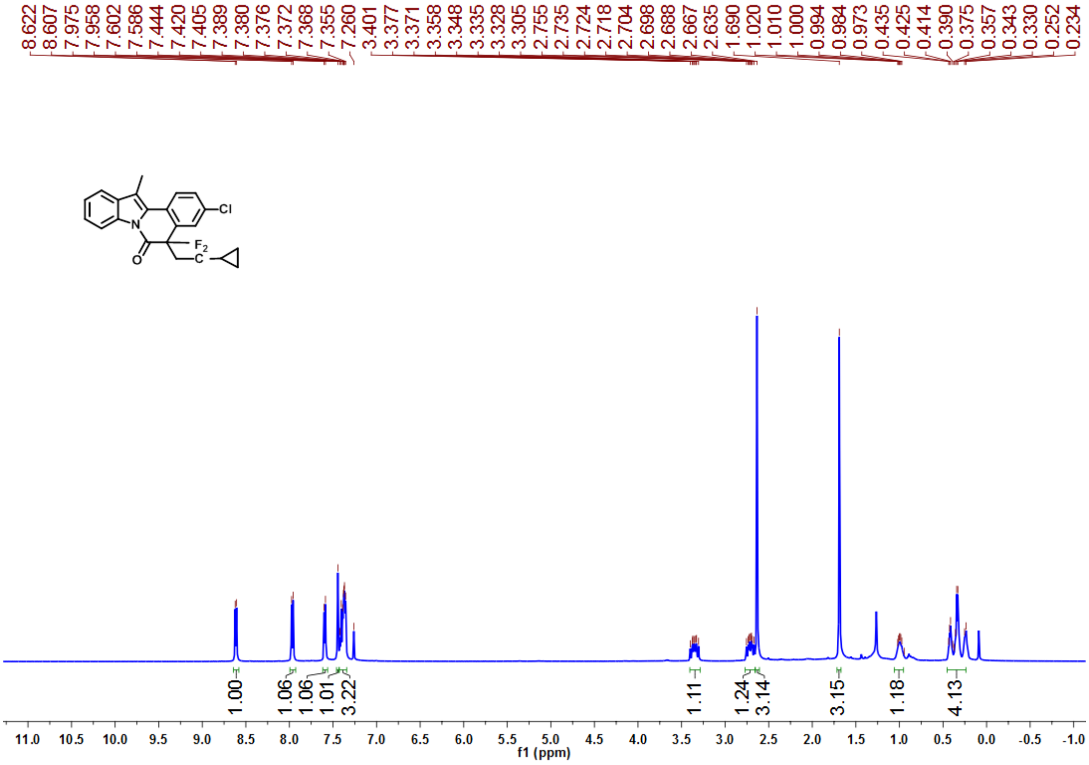

5i-^{13}C{^{1}H} NMR (125 MHz, $CDCl_3$)

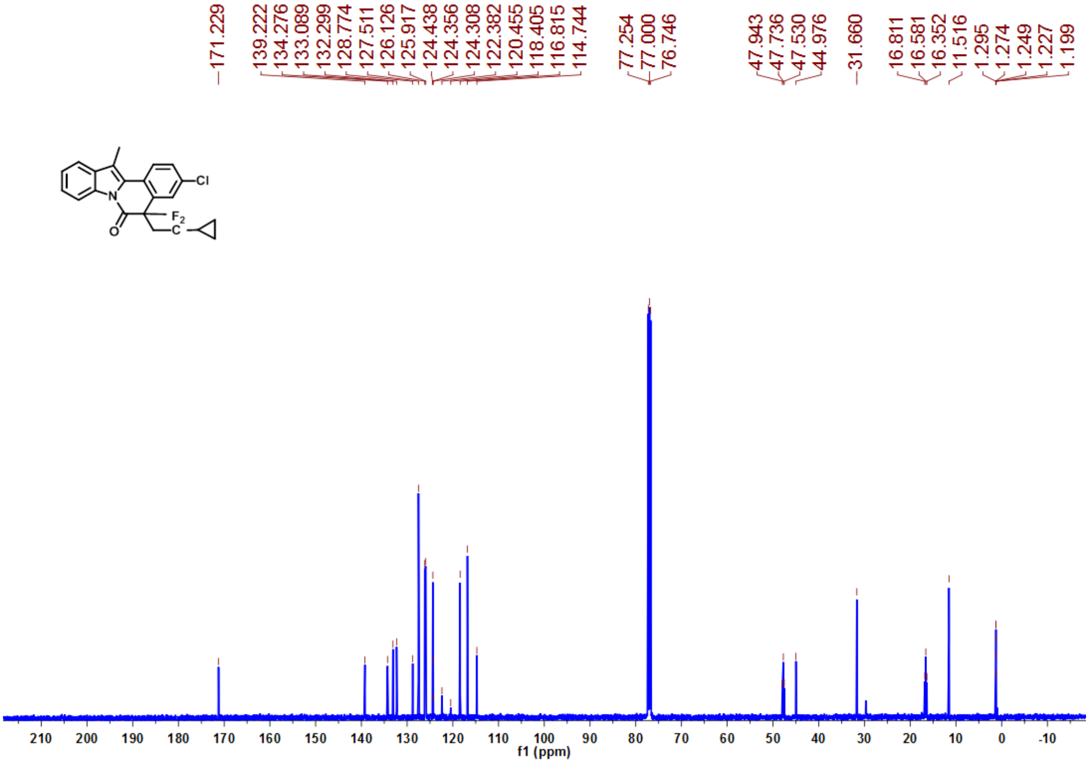

5i-^{19}F NMR (471 MHz, $CDCl_3$)

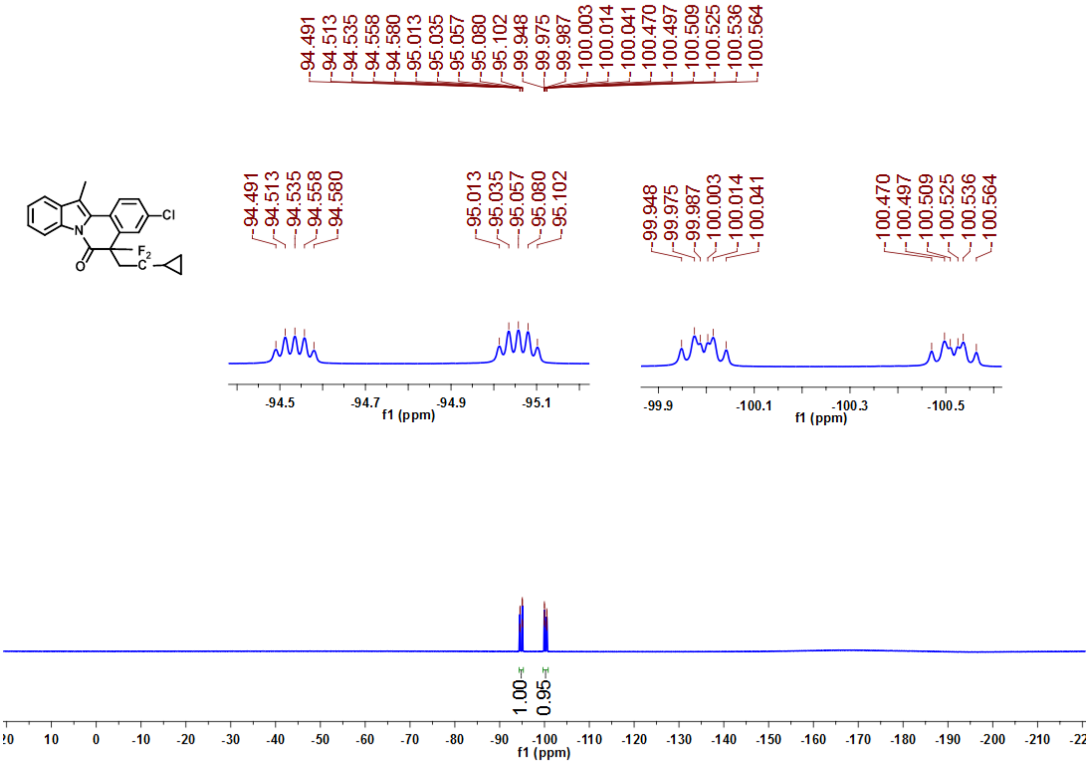

Supplement: Supplementary file 1 [file molecules-29-00973-s001.zip › molecules-2845669-supplementary.pdf]
